# Haptic Exploratory Behavior During Object Discrimination: A Novel Automatic Annotation Method

**DOI:** 10.1371/journal.pone.0117017

**Published:** 2015-02-06

**Authors:** Sander E. M. Jansen, Wouter M. Bergmann Tiest, Astrid M. L. Kappers

**Affiliations:** MOVE Research Institute, VU University Amsterdam, the Netherlands; University of California, Merced, UNITED STATES

## Abstract

In order to acquire information concerning the geometry and material of handheld objects, people tend to execute stereotypical hand movement patterns called haptic Exploratory Procedures (EPs). Manual annotation of haptic exploration trials with these EPs is a laborious task that is affected by subjectivity, attentional lapses, and viewing angle limitations. In this paper we propose an automatic EP annotation method based on position and orientation data from motion tracking sensors placed on both hands and inside a stimulus. A set of kinematic variables is computed from these data and compared to sets of predefined criteria for each of four EPs. Whenever all criteria for a specific EP are met, it is assumed that that particular hand movement pattern was performed. This method is applied to data from an experiment where blindfolded participants haptically discriminated between objects differing in hardness, roughness, volume, and weight. In order to validate the method, its output is compared to manual annotation based on video recordings of the same trials. Although mean pairwise agreement is less between human-automatic pairs than between human-human pairs (55.7% vs 74.5%), the proposed method performs much better than random annotation (2.4%). Furthermore, each EP is linked to a specific object property for which it is optimal (e.g., Lateral Motion for roughness). We found that the percentage of trials where the expected EP was found does not differ between manual and automatic annotation. For now, this method cannot yet completely replace a manual annotation procedure. However, it could be used as a starting point that can be supplemented by manual annotation.

## Introduction

In contrast to most other senses, haptic perception requires physical contact between the body and an object of inquiry. Both cutaneous and proprioceptive sensory information can be used to explore handheld objects and evaluate their properties. Overall, we can get a rough estimate of most of its properties, and thereby identify an object, just by grasping and lifting it. However, sometimes it is required to have a more precise estimate concerning a specific property. For example, in order to insert a needle into specific tissue, a physician needs to judge its compliance in order to prevent damage to underlying tissue. In another example, when selecting a certain key from a key ring in a pocket, local differences in shape need to be considered in order to identify the correct key.

The human hand contains sensory receptors in the skin, muscles, and tendons [1, 2]. Relative motion between the skin and the surface of an object stimulates all these receptors differently. Certain hand movements stimulate receptors in such a way that yield predictable responses. Estimation of a particular object property then follows from the integration of these responses over time taking into account the relative movement over this interval. For example, when hefting an object in the hand there is little relative motion between the skin and the object surface. However, mechanoreceptors in the skin will react to changes in pressure resulting from the hefting. When this signal is combined with proprioceptive information from muscle spindles, its interpretation yields an estimate of object weight.

In 1987, Lederman and Klatzky [3] proposed a set of such stereotyped hand movements called Exploratory Procedures (EPs) that are optimal for retrieving specific object information. For example, *Lateral Motion* is the optimal EP for the assessment of roughness while *Enclosure* can be used to estimate volume and global shape. Such a taxonomy of hand movements is very useful when investigating haptic exploratory strategies between different groups of people (e.g., children, adults, visually impaired). In addition, these movement strategies could be implemented as distinct motor commands to drive artificial hands used to explore objects and materials in remote and dangerous environments. Currently, the annotation of haptic exploration episodes is done by observing video recordings of object handling and manually annotating it with the EPs [4]. This is a laborious task that involves substantial subjectivity on the part of the observer. In addition, it is prone to missing important behavior due to limitations in visibility as well as observers’ attention. An automatic annotation method could greatly improve the efficiency, accuracy, and consistency of these analyses.

Automatic behavior classification systems have been developed both for human and rodent behavior analysis. Applications for such systems include video surveillance of crowds [5] and drug testing on rats [6]. In such systems, computer vision is employed to identify behavioral patterns such as crowding, fighting, eating and sleeping. In addition, increasing interest goes out to gesture recognition in general and sign language in particular. Several sign recognition systems have been developed based on: adaptive fuzzy expert systems [7], hidden Markov models [8], and neural networks [9]. In addition to the contribution to the behavioral annotation domain, this paper could also be beneficial to the aforementioned fields by focussing on a key movement parameters to study (complex) behavior. This could lead to a better understanding of the motivation behind certain movements.

Even though both types of movements involve the hands, there are fundamental differences between gestures and exploratory procedures. The former involves well-defined hand postures, while the latter requires an object whose properties limit the hand movements performed on it. Furthermore, gestures are executed to convey information, while exploratory hand movements are performed to extract information. Finally, there exists a ground truth for gesturing which does not exist for exploratory hand movements. In gesturing, one specific word or sentence is communicated which allows for evaluation against this ground truth and direct comparison between different recognition systems.

Currently, no method exists to annotate episodes of free haptic object exploration with EPs (or any other taxonomy for that matter). However, there has been some work on classification of haptic exploration in 2D. For a search task on a haptic display, Van Polanen [10] and colleagues used two criteria to classify hand movements into three movement types. Moreover, in a recent paper [11] we found that a subset of EPs could be identified by analyzing a few hand dynamics and contact force parameters. However, there were some limitations to that approach. First, movements were performed on raised surface stimuli that did not permit free exploration. Second, this method was limited to classifying entire exploration trials into single EPs. With the current study we aim to develop an automatic annotation method capable of handling bimanual exploration of 3D objects. Moreover, this method allows for annotation of a trial with multiple intervals of multiple EPs.

The remainder of the paper is organized in three parts. In part I, we describe the data gathering procedure, which consisted of a discrimination task concerning several haptic object properties. Part II describes the manual and automatic annotation procedures which are applied to the data from part I. In part III, the results of the two methods are compared in order to validate the proposed automatic annotation method. This is followed by a general discussion section.

## Part I: Data Gathering

This part describes the discrimination experiment conducted to gather hand movement data and video recordings on which the manual and automatic annotation procedures are based.

### Participants

Five paid participants (one male) gave written informed consent and took part in the data gathering experiment. Their mean age was 22 years (SD = 2). All of them were strongly right-handed according to Coren’s test [12]. All followed the same experimental procedure. The study was approved by the Ethics Committee of the Faculty of Human Movement Sciences of VU University Amsterdam.

### Materials and Methods

**Fig 1:**
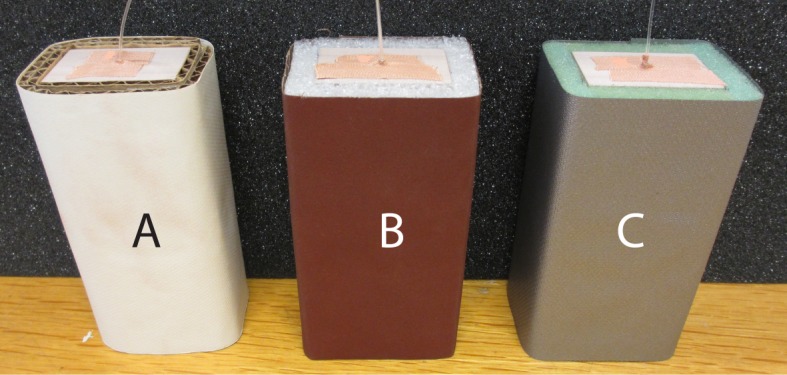
Stimuli used during haptic discrimination. Because of different mid and outer layers, all differed in roughness, hardness, volume, and weight. Stimulus B contains an actual sensor, while stimuli A and C contain dummies.

*Stimuli*—Three stimuli were used during the data gathering procedure. All consisted of a cuboid shaped core of Ebaboard (Ebalta Kunststoff GmbH) (30 × 40 × 120 mm) which was covered on four faces by a compressible mid layer and wrapped in a textured outer layer. The thickness and compliance of the mid layer combined with the variation in roughness from the outer layer resulted in stimuli that differed from each other in roughness, hardness, volume, and weight. The exact values for the different properties are irrelevant in this approach. We are only interested in the behavior that is displayed as a function of the required property. Stimulus A has a mid layer consisting of two layers of corrugated cardboard, an outer layer of structured paper, a volume of 357 cm^3^, and it weighs 141 g. Stimulus B has a mid layer of large cell PE foam, an outer layer of sandpaper, a volume of 382 cm^3^, and it weighs 131 g. Stimulus C has a mid layer of small cell PE foam, an outer layer of duct tape, a volume of 390 cm^3^, and it weighs 133 g. In addition, a narrow tunnel (∅ 2.5 mm) was drilled from the top of each stimulus to accommodate placement of an actual sensor (stimulus B) or dummy (stimuli A & C) in its center. See Fig. 1 for a photo of the stimuli.

**Fig 2:**
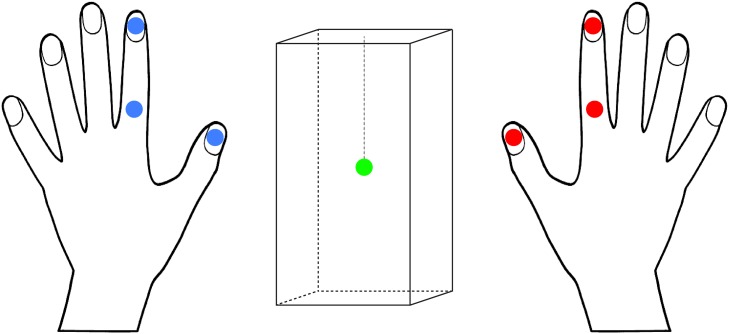
Sensor placement. In total, seven 6 DoF electromagnetic sensors were used during the data gathering procedure. Three on each hand and one inside the stimulus.

*Motion Tracking*—During haptic exploration of each stimulus, the position and orientation of the index fingers and thumbs on both hands were registered with a sampling frequency of 300 Hz. In addition, a sensor was placed at the center of stimulus B. For each index finger, sensors were placed at the nail and center of the proximal phalanx. In addition, sensors were positioned on both thumbnails. Fig. 2 depicts the positions of these electromagnetic tracking sensors (3D Guidance TrakSTAR).

### Design and Procedures

At the start of the experimental session participants gave informed consent after which the sensors were attached to their hands and they were blindfolded. Each trial was organized as follows: index fingers of both hands were placed at starting positions (marked tangibly on the table) approximately 20 cm on both sides of a fixed object position. The first stimulus was then placed at the object position. After the experimenter verbally stated which of the four properties (roughness, hardness, volume, or weight) was to be explored, participants used their left hand to lift the stimulus and started exploring the required property (bimanually if they preferred). When they felt that the information was acquired satisfactorily, the object was put down and they moved both hands back to the starting positions. The experimenter then placed the second stimulus on the table and they were allowed to explore that. The task was to decide whether the two stimuli differed on the required property. Participants verbally stated “equal” or “different” which concluded the trial. For every trial one of the objects was stimulus B (containing an actual sensor), while the other could be each of the three stimuli. However, participants were led to believe that many combinations of the properties existed (in the form of many stimuli) and that any of these could be presented to them. This was done to ensure that they explored each stimulus extensively to acquire information on the required property instead of identifying it and comparing the stimuli from memory. Verbal reports afterwards confirmed that this manipulation had worked. Moreover, data were only gathered during exploration of stimulus B because it contained a real sensor. In total, 24 trials were performed: 4 (properties) × 3 (pair combinations) × 2 (presentation order). However, only trials where stimulus B was presented first were used for analysis. The reason for this is that the first stimulus is explored more extensively because of its role as a reference in the comparison. In contrast, exploration of the second stimulus was often very brief.

## Part II: Annotation

This part describes both the manual and automatic annotation procedures. The former utilizes video recordings of the exploration sessions while the latter is based on sensor data.

### Manual EP Annotation

In order to validate the automatic annotation method, it will be compared to the output of a manual annotation procedure. To that end, video recordings were made during data gathering which were then annotated by three independent observers.

#### Participants

Three paid participants (1 male) took part in the manual annotation procedure after giving written informed consent. Their mean age was 21 years (SD = 0.6). All followed the same experimental protocol and none of them participated in the discrimination experiment described in Part I. The study was approved by the Ethics Committee of the Faculty of Human Movement Sciences of VU University Amsterdam.

#### Materials and Methods

**Fig 3:**
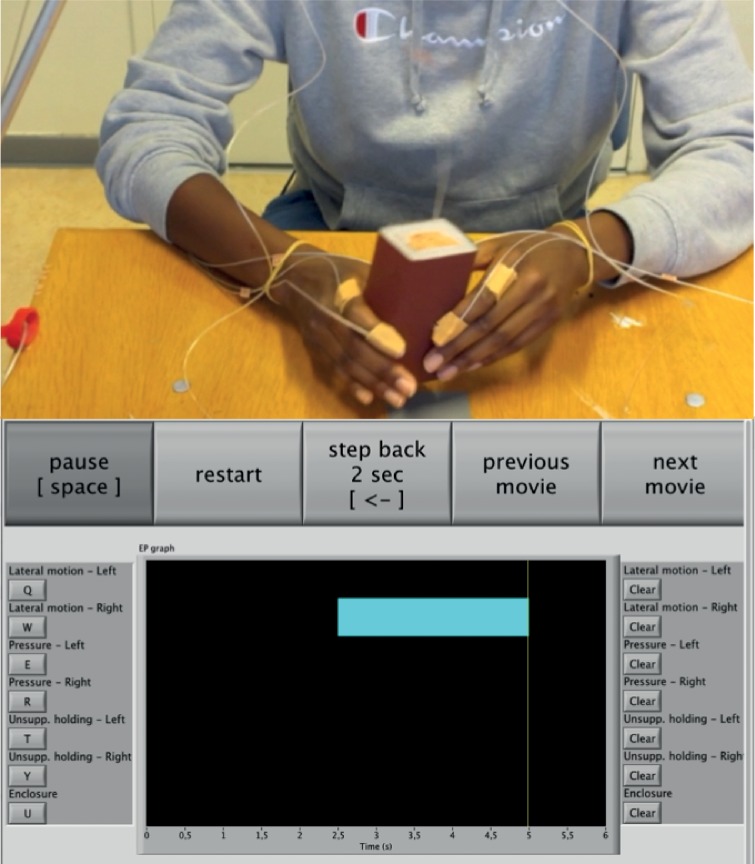
Manual annotation setup. The top monitor shows the recorded video in fullscreen while the lower monitor enables annotation and playback options.

Custom annotation software was created using LabVIEW (National Instruments, version 2011). A vertical dual monitor setup allowed participants to view the video while annotating the EPs on a timeline depicted on a separate monitor. Each of the seven behaviors (unimanual LM, PR, UH, and bimanual EN) was assigned a keyboard shortcut to hold down whenever that particular behavior was observed. Participants were able to control (frame by frame) playback using a control knob (Powermate by Griffin Technologies). Whenever participants felt they made a mistake they could adjust the annotation by decreasing or increasing the length of an interval. Alternatively, a specific EP could be cleared altogether in order to start over for that trial. Video clips were recorded with a resolution of 640 × 480 at 30 Hz. Fig. 3 shows screenshots of both monitors during a typical annotation trial.

#### Design and Procedures

A total of 60 video clips were used in this experiment: 5 people × 4 object properties × 3 trials. One trial was excluded from analysis because its video was accidentally replaced by the video of a different trial (which was then erroneously annotated). Therefore 59 trials were analyzed. All three participants viewed and annotated all clips in a randomized order. Prior to the start of the experiment a few practice clips were annotated together with the experimenter. This was done to ensure that the participants correctly understood the procedure and adhered to the EP definitions as given to them in written form. These were: *Lateral Motion* (LM): “The skin is passed laterally across a surface, producing shear force”. *Pressure* (PR): “Force is exerted on the object against a resisting force”.*Unsupported Holding* (UH): “The object is held while the hand is not externally supported”. *Enclosure* (EN): “The fingers are molded closely to the object surface”.

Each trial consisted of the following steps that could be repeated as often as preferred by the participant:

1. The entire clip was viewed in real time
2. For each observed EP, the clip was viewed again using the frame-by-frame and fast forward playback options to annotate the observed intervals of that EP.
3. The fully annotated clip was reviewed in real time. Only when the participant was fully satisfied with the annotation did he/she proceed to the next trial.

### Automatic EP Annotation

The current method enables the annotation of four of the main EPs described by Lederman and Klatzky [3]. These were chosen based on their ability to optimally extract the object properties that participants were required to assess in Part I: *Lateral Motion* for roughness, *Pressure* for hardness, *Unsupported Holding* for weight, and *Enclosure* for volume. After filtering the positional data (low-pass second-order Butterworth filter with a cutoff frequency of 6 Hz), a set of variables is computed from the position and orientation data. These variables were chosen such that they enable discrimination between the EPs.

The remainder of this section is used to describe the EPs and variables. First, for each of the four EPs, we state its description as given by Klatkzy and Reed [13], followed by our implementation of this and the criteria chosen for this specific setup. Then, we explain how the variables used to describe these EPs are computed from the motion tracking data. At each time step (3.33 ms), the variables for both hands are compared to the criteria and each hand might be annotated with one of the four EPs or left blank if none can be matched. Note that the *Enclosure* EP requires both hands to match the criteria while the three others can be annotated unimanually. The values belonging to the qualifications ‘low’, ‘high’, ‘small’, and ‘close’ are specific for the current setup. This method is implemented in Matlab (The Mathworks, version 2013b) and is subdivided into several parts which are executed consecutively.

#### Implementation of EPs

*Lateral Motion* (LM): One hand holds the stimulus while the other rubs against the surface.

- At least one of the fingernails should be close to the surface (< 20 mm) and have a high relative speed compared to the stimulus (> 0.10 m/s).
- The thumb on the opposite hand should have a low relative speed compared to the stimulus (< 0.05 m/s). Although the index finger is often used to grasp the stimulus (and therefore positioned close to the surface), it is not required as also other fingers might be used. However, for a grasp, the thumb is required.

*Pressure* (PR): The instrumented stimulus (stimulus B) consists of a compliant mid layer. Therefore, the thumb as well as one of the fingers will deform it when applying pressure.

- The size of the minimal convex hull that encloses the thumb and the stimulus is less than when the stimulus is initially lifted from the table.
- As the result of a pinching motion, there is a decrease in this hull size prior to exceeding the above mentioned threshold. This criterion is added to prevent annotation due to an outwards pointing thumb (and subsequent overestimated hull size) when picking up the stimulus.

*Unsupported Holding* (UH): We assume that unsupported holding will be performed unimanually. Due to the size and weight of the stimulus it is expected that participants perform this EP unimanually. Pilot recordings confirmed this.

- The thumb is close (< 20 mm) to the stimulus surface.
- Relative speed between the sensors on both the thumb and index finger (of one hand) and the stimulus is low (< 0.05 m/s).
- For the opposite hand, the virtual midpoint between the thumb and index finger lies outside the stimulus volume.
- The sensor on the thumb of the opposite hand is not close (> 20 mm) to the stimulus surface.

*Enclosure* (EN): The hand configuration is stable for a moment without deforming the object. Furthermore, the stimuli used in this study can only be enclosed using both hands. Therefore, this EP cannot be annotated unimanually.

- Relative speed between all four fingernails and the stimulus is low (< 0.05 m/s).
- For both hands, the virtual midpoint between the thumb and index finger lies within the stimulus volume.
- For both hands, the rate of change of the index finger angle is low (< 80 deg/s).

#### Computation of Variables

*Relative Speed*—For all four sensors placed on fingernails (both index fingers and thumbs), the relative speed compared to the stimulus is calculated. This is defined as the rate of change of the Euclidean distance between the sensor on the nail and the sensor at the center of the stimulus.

*Convex Hull Size*—For both hands, we compute the size of the smallest 3D convex hull that encloses the entire stimulus and the thumbnail sensor. In addition, the rate of change of this variable is computed.

*Stimulus Elevation*—This is defined as the *z*-coordinate of the sensor placed inside stimulus B. In addition, the rate of change of this variable is computed.

*Distance to Stimulus Surface*—Each of the surface planes is represented as a grid of 45 points. For all four sensors placed on fingernails, the Euclidean distance to the closest point on the stimulus surface was calculated.

*Virtual Midpoint within Stimulus*—For both hands, a virtual point was computed that lies midway between the sensors placed at index finger and thumb nails. Then, for each time step it is checked whether these points lie within the stimulus volume.

*Index Finger Angle*—For both index fingers this is calculated as the angle between the orientation of the sensor on the nail and the sensor on the proximal phalanx of the index finger. In addition, the rate of change of this variable is computed.

#### Post Processing

For each time step, the computed variables are checked against the set of criteria for each EP. Whenever all criteria for an EP are met, it is assumed that the particular behavior is displayed and annotation takes place accordingly. After this initial annotation, a post-processing phase is executed. First, short time intervals (less than 50 ms) that lie between two intervals of a certain EP, will be filled with that same annotation. Then, each annotated interval is checked against a predefined minimum duration (17 to 250 ms depending on EP). Everything shorter is deleted. Finally, there is a check whether incompatible EPs are annotated simultaneously (e.g., lateral motion and pressure with the same hand). If this is the case, then one of them is deleted according to the following dominance ranking from high to low: LM—UH—PR—EN. This order is based on the likelihood that an EP is annotated combined with the complexity of the movement. For instance, *Pressure* will cancel *Enclosure* when annotated simultaneously because the bimanual execution of the former could be described as the latter plus a deformation of the object. The post-processing routine is executed twice to ensure clean up of short intervals resulting from the simultaneity resolve.

## Part III: Results

This part describes how the automatic annotation method compares to the manual annotation. Furthermore, we investigate the relationship between the required object property and the expected EP.

### Comparison between Manual and Automatic Annotation

The output of both manual and automatic annotation procedures is compared in two ways, a general and a more specific approach. First, for each rater and each trial we establish the main and secondary EP based on the total duration of each EP for that particular trial. Often, at least two EPs were annotated during a trial, which is why we chose to look at the two with the longest duration. If only one or no EPs are annotated, the result is one or two blank outputs, respectively. We then evaluate the overlap in output between the automatic rater and the human raters. In 74.6% of all trials, either the main or secondary EP according to the automatic rater corresponds to the main or secondary EP according to at least one of the human raters. In comparison, for random annotation this is 53.6% (averaged over 10 runs of random annotations for all trials).

**Fig 4:**
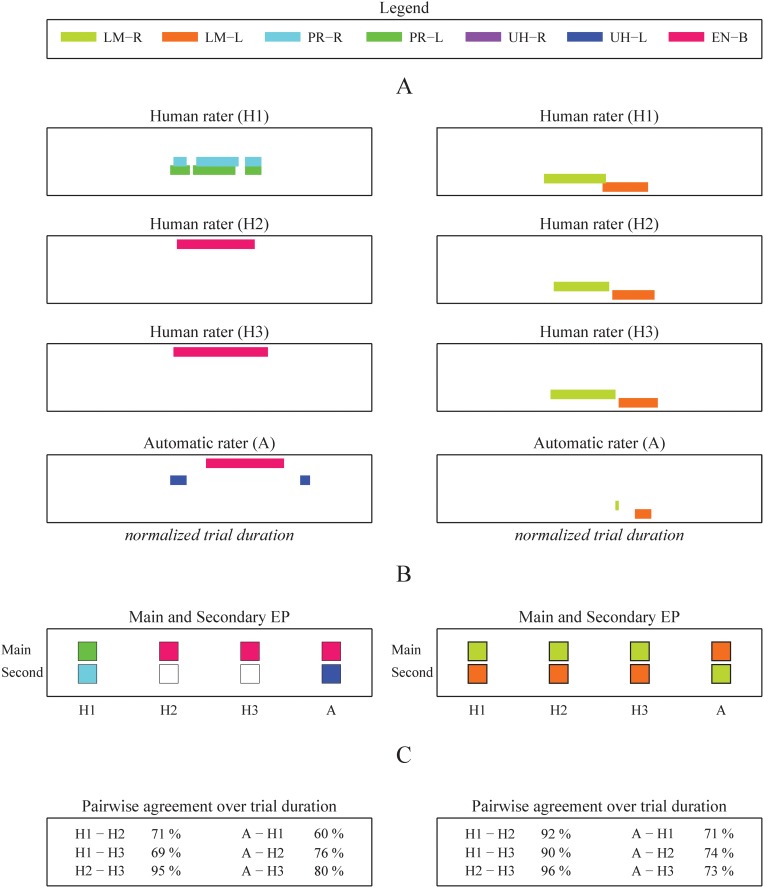
Annotation of two example exploration trials. Panel A: the left and right columns show output from all raters for a volume and roughness trial respectively. The Possible EPs are: *Pressure* (PR), *Lateral Motion* (LM), *Unsupported Holding* (UH), and *Enclosure* (EN). The width of the box represents the normalized trial duration and each annotated interval is color coded according to the legend. Panel B shows the main and secondary EP based on total duration annotated by each rater. The same legend is used to indicate the EP and hand. Panel C displays the pairwise agreement as a percentage of trial duration.

More specifically, we can compare the annotation outputs by evaluating the agreement over time. The output for this method is the percentage of time that two raters agree about the presence of all EPs. Here, the exact timing of the annotated EP plays a role because the evaluation of the agreement takes place at each time step. For each trial, six percentages of agreement are computed (one for each possible pair based on three human and one automatic rater). See Fig. 4 for the output of two trials accompanied by visual representations of the comparisons.

As a first analysis, we want to investigate if the EP agreement depends on the pair. Therefore, we performed a simple one-way ANOVA on the agreement data with *pair* (6 levels) as the only factor. The result shows that there is an effect of *pair* on mean agreement, *F*(3.2, 186) = 44; *p* < 0.001. Post-hoc analysis reveals that the three human-human pairs have higher mean agreement percentages (76, 75, 73) than the human-automatic pairs (56, 56, 55), *p* < 0.001 for all.

**Fig 5:**
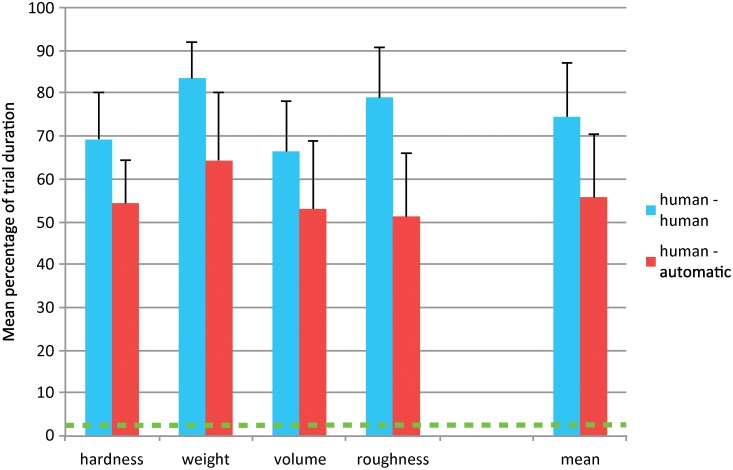
Mean percentage of trial duration that a pair of raters is in agreement over the annotation of all EPs. Data are shown for each property separately and as the mean over all trials. The green dashed line indicates mean agreement between human and random annotation. Error bars represent standard deviation.

The average of the three pairs constitutes a mean agreement percentage for each pair type. For the agreement data, A 4 × 2 mixed factorial ANOVA was performed with *object property* (4 levels) as the between-group variable and *pair type* (human-human vs. human-automatic) as the repeated measures variable. Overall, there was an effect of *object property* on the annotation agreement, *F*(3, 55) = 5.4; *p* < 0.01. Post-hoc analysis revealed that agreement was higher for weight trials compared to hardness or volume trials. In addition, there was a main effect of *pair type*, indicating higher agreement between a pair of human raters (74.5%) compared to a human-automatic pair (55.7%), *F*(1, 55) = 97; *p* < 0.001. This means that on average, the automatic annotation agrees with manual annotation about the presence of all EPs for 55.7% of the trial duration (ranging between 51.2% and 64.2% for the different object properties). In comparison, the agreement between manual annotation and random annotation (one random EP for the entire duration of the trial) is 2.4%. See Fig. 5 for a graphical representation of these results.

### Object Properties & Expected EPs

In this paper we focus on the proposed automatic annotation method by comparing its output to manual annotation. Nevertheless, we would also like to investigate for the current setup the relationship between object properties and EPs. Therefore, we analyze how often the expected EP (which is optimal for a specific property) is found as the main or secondary EP by the raters. The expected links are: *Lateral Motion* for roughness, *Pressure* for hardness, *Unsupported Holding* for weight, and *Enclosure* for volume. The dependent variable is the percentage of trials where the expected EP was found as either the main or secondary EP by the raters.

**Fig 6:**
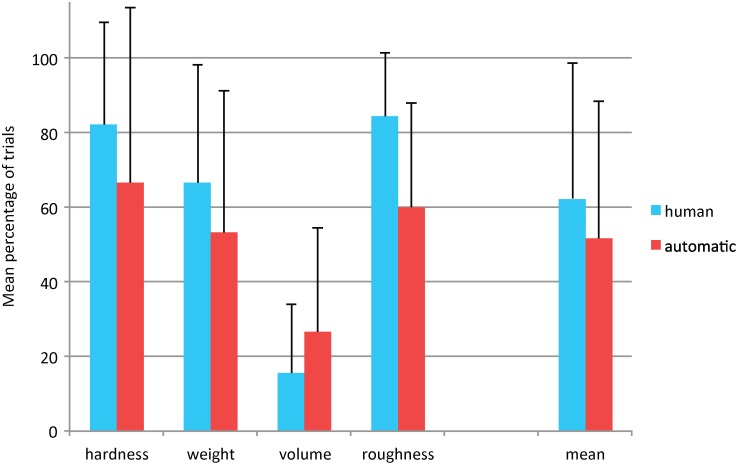
Percentage of trials for which the expected EP was found as the main or secondary EP. The expected links are: *Lateral Motion* for roughness, *Pressure* for hardness, *Unsupported Holding* for weight, and *Enclosure* for volume. This percentage is given as a function of object property and rater type. Error bars represent standard deviation.

A 4 × 2 mixed factorial ANOVA was performed with *object property* (4 levels) as the between-group variable and *rater type* (human vs. automatic) as the repeated measures variable. There is a significant main effect for *property*, *F*(3, 16) = 6.6; *p* < 0.01; in trials where volume was the required property, the percentage of trials where the expected EP (i.e., Enclosure) was found as the main or secondary EP was smaller than in trials where hardness (*p* < 0.01) and roughness (*p* = 0.01) were being assessed. The percentage of trials did not differ between raters, nor was there a significant interaction between *rater type* and *property*. See Fig. 6 for a graphical representation of these results.

## General Discussion

We have proposed a new method for automatic annotation of haptic exploration with a subset of the Exploratory Procedures (EPs) proposed by Lederman and Klatzky [3]. The method is based on the assumption that a set of key parameters can describe these EPs. For example, *Lateral Motion* is characterized by high relative motion between the skin of one hand and the surface, while the other hand holds the object. Several distance and speed parameters can describe this behavior. The pattern of movement (i.e., up-down or circular) and the amount of force applied to the surface are irrelevant in this case. Other EPs included in this method are *Pressure*, *Unsupported Holding*, and *Enclosure*. At each time step during exploration, the values for all parameters are checked against sets of criteria defined for each EP. If all criteria for a particular EP are met, it is assumed that this type of movement is performed.

The proposed automatic annotation method is applied to data gathered from a discrimination experiment where participants were required to determine if two objects differed on a particular object property (e.g., hardness, roughness). In order to validate the method, it is compared to manual annotation of the same exploration episodes. Three independent observers annotated video recordings of all trials using the same set of possible EPs. Results show that in 74.6% of all trials there was overlap between the main and secondary EP (based on duration) as indicated by the automatic and manual annotations. In addition, we investigated the percentage of time that a pair of observers is in agreement about the presence of all EPs. The mean agreement between the automatic annotation and a human observer was 55.7% (compared to 74.5% for a human pair and 2.4% for random annotation). The difference in agreement between the two types of rater pairs may be explained by the fact that the automatic rater is more sensitive than the human observers. Often, it annotates small patches of *Lateral Motion* and *Pressure* which are not seen by people. This does not mean that the behavior did not take place. It just shows that people have learned to ignore (perhaps insignificant) details when presented with some other clearly visible behavior. Furthermore, it seems that agreement was higher during annotation of weight trials compared to hardness and volume. Presumably, this is due to the unambiguous execution of the unsupported holding EP. This is the optimal procedure for weight perception and seems to be easily detected by both types of observers.

Related to this, we examined how often the expected EP for each trial (based on the proposed links by Lederman and Klatzky [3]) was found as a function of rater type and object property. The results indicate that the automatic annotation did not differ significantly from the manual annotation with respect to this expected EP. However, for the volume trials, the expected EP (i.e., *Enclosure*) was found less often compared to the other properties. One explanation for this is the fact that our stimuli are block-shaped and therefore their volume could be determined by estimating the three orthogonal dimensions with pinch grasps instead of enclosing it with two hands. It should be noted here that the variance of this measure was very high, meaning that the participants used different EPs when exploring a particular object property.

The current approach contains several limitations. First, only four EPs can be annotated at this moment. This was done to limit the amount of time required for both the acquisition of the discrimination data and the implementation of the different EPs. Second, the method assumes that objects deform under small amounts of pressure, which is not true for all handheld objects. Therefore, a useful addition to the method would be to add multiple small force-sensitive resistors to the surface of the object. This enables registration of the normal force produced on a surface in addition to the movement of fingers. Ideally, this would lead to instrumented objects that do not require any motion registration on the hands to recognize stereotypical exploratory behavior.

Interestingly, for some trials both the human observers and the automatic annotation method did not recognize any of the possible EPs. From an evaluation point of view, this is a good result because the annotation outputs correspond. However, this indicates that people sometimes perform hand movements that simply do not fall into one of the four categories defined as EPs. This could be due to the limited set of EPs involved in this study. However, this seems unlikely since these were the expected EPs based on the required object properties. This is an interesting observation that justifies further investigation. Perhaps the original taxonomy could benefit from extending the definitions or adding new EPs. However, we first need to gather more support for this by investigating other exploratory tasks as well as stimulus sets.

In order to improve this method, we need to know why certain behavior observed by human raters was not recognized by our system and vice versa. One explanation for this could be that the criteria used in the current implementation are constant over the participants from which the data are gathered even though their behavior is not identical. What is a relatively fast movement for one person, might be slow for another. The addition of a calibration phase would be very useful in this regard. By analyzing stereotypical movements, these criteria could be determined automatically for each participant.

Overall, it can be concluded that the proposed method shows some promising results. However, it is not yet on par with manual annotation. Note that in this case, manual annotation is not the golden standard. On average, a pair of human annotators agree for roughly 75% (ranging from 66–83%) of the duration of a trial, which indicates the subjectivity of manual annotation. This is one of the main reasons why an automatic system is preferable; one observer might see something which another does not. An automatic system has the benefits of being consistent, efficient, and transparent in its annotation. For now, it seems that the proposed method could be used as a starting point for human observers that they can edit to their liking. In that way they are encouraged to think about reasons for adding, deleting, or altering EP intervals.

## Supporting Information

S1 Annotation Output (PDF)

S2 Annotation Output (PDF)

S3 Annotation Output (PDF)

S4 Annotation Output (PDF)

S5 Annotation Output (PDF)

S1 Data Example (MAT)

S1 Matlab Script (M)
