# Supplementary material for: Haptic Exploratory Behavior During Object Discrimination: A Novel Automatic Annotation Method
Source: PLoS One. 2015 Feb 6;10(2):e0117017. doi: 10.1371/journal.pone.0117017 (PMC4319767; doi:10.1371/journal.pone.0117017)
Supplement: S1 Annotation Output — (PDF) [file pone.0117017.s001.pdf]

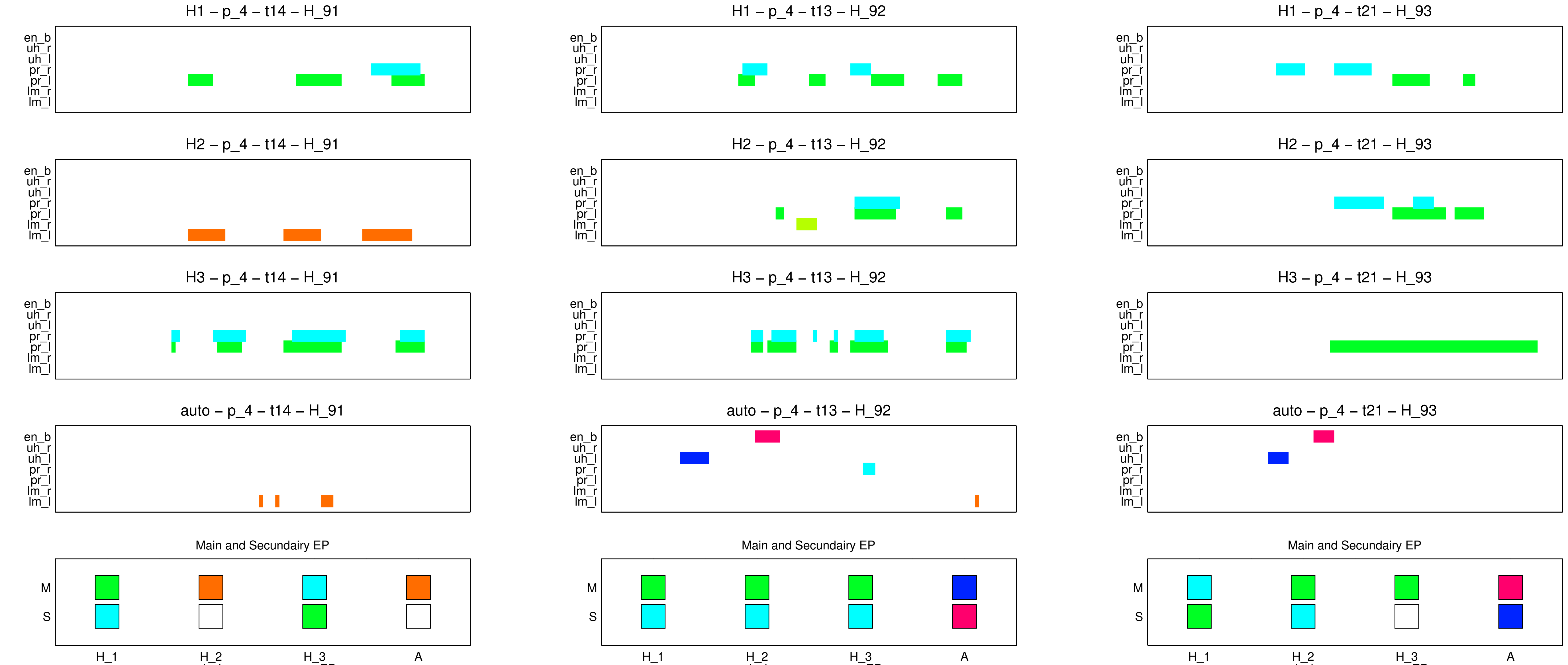

pairwise agreement per EP

	L–l	L–r	P–l	P–r	U–l	U–r	E–b		all
H_1–H_2	67	100	72	87	100	100	100		59
H_1–H_3	100	100	80	66	100	100	100		58
H_2–H_3	67	100	68	67	100	100	100		50
A–H_1	92	100	72	87	100	100	100		63
A–H_2	61	100	100	100	100	100	100		61
A–H_3	92	100	68	67	100	100	100		60

pairwise agreement per EP

	L–l	L–r	P–l	P–r	U–l	U–r	E–b		all
H_1–H_2	100	94	79	85	100	100	100		66
H_1–H_3	100	100	67	75	100	100	100		58
H_2–H_3	100	94	84	74	100	100	100		68
A–H_1	98	100	74	89	92	100	93		55
A–H_2	98	94	81	92	92	100	93		59
A–H_3	98	100	69	74	92	100	93		56

pairwise agreement per EP

	L–l	L–r	P–l	P–r	U–l	U–r	E–b		all
H_1–H_2	100	100	92	83	100	100	100		76
H_1–H_3	100	100	63	82	100	100	100		55
H_2–H_3	100	100	71	81	100	100	100		65
A–H_1	100	100	86	82	94	100	94		61
A–H_2	100	100	78	81	94	100	94		54
A–H_3	100	100	49	100	94	100	94		39

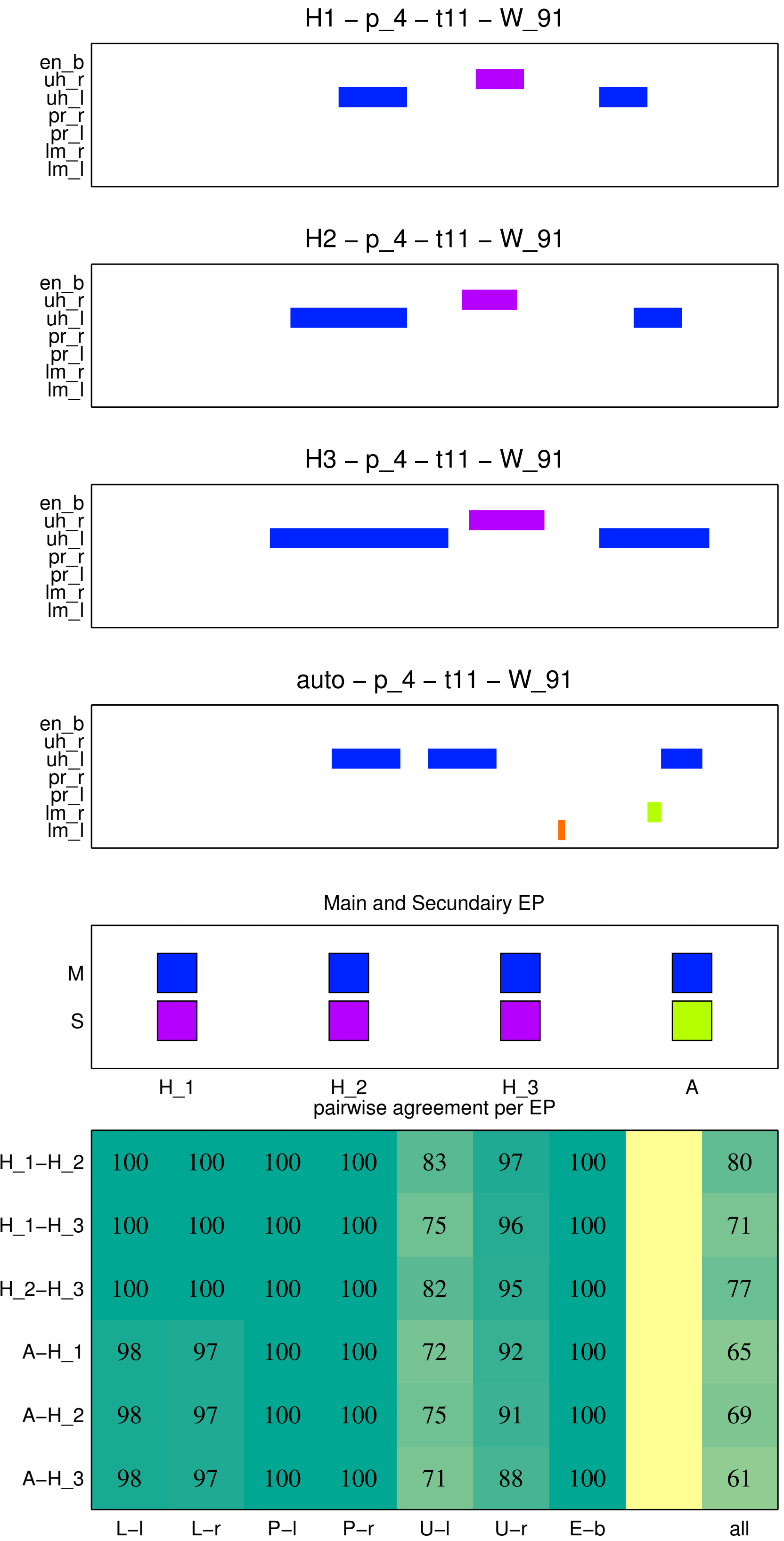
H1 – p_4 – t11 – W_91
H2 – p_4 – t11 – W_91
H3 – p_4 – t11 – W_91
auto – p_4 – t11 – W_91
en_b
uh_r
uh_l
pr_r
pr_l
lm_r
lm_l
Main and Secundairy EP
M
S
H_1
H_2
H_3
A
pairwise agreement per EP

	L–l	L–r	P–l	P–r	U–l	U–r	E–b		all
H_1–H_2	100	100	100	100	83	97	100		80
H_1–H_3	100	100	100	100	75	96	100		71
H_2–H_3	100	100	100	100	82	95	100		77
A–H_1	98	97	100	100	72	92	100		65
A–H_2	98	97	100	100	75	91	100		69
A–H_3	98	97	100	100	71	88	100		61

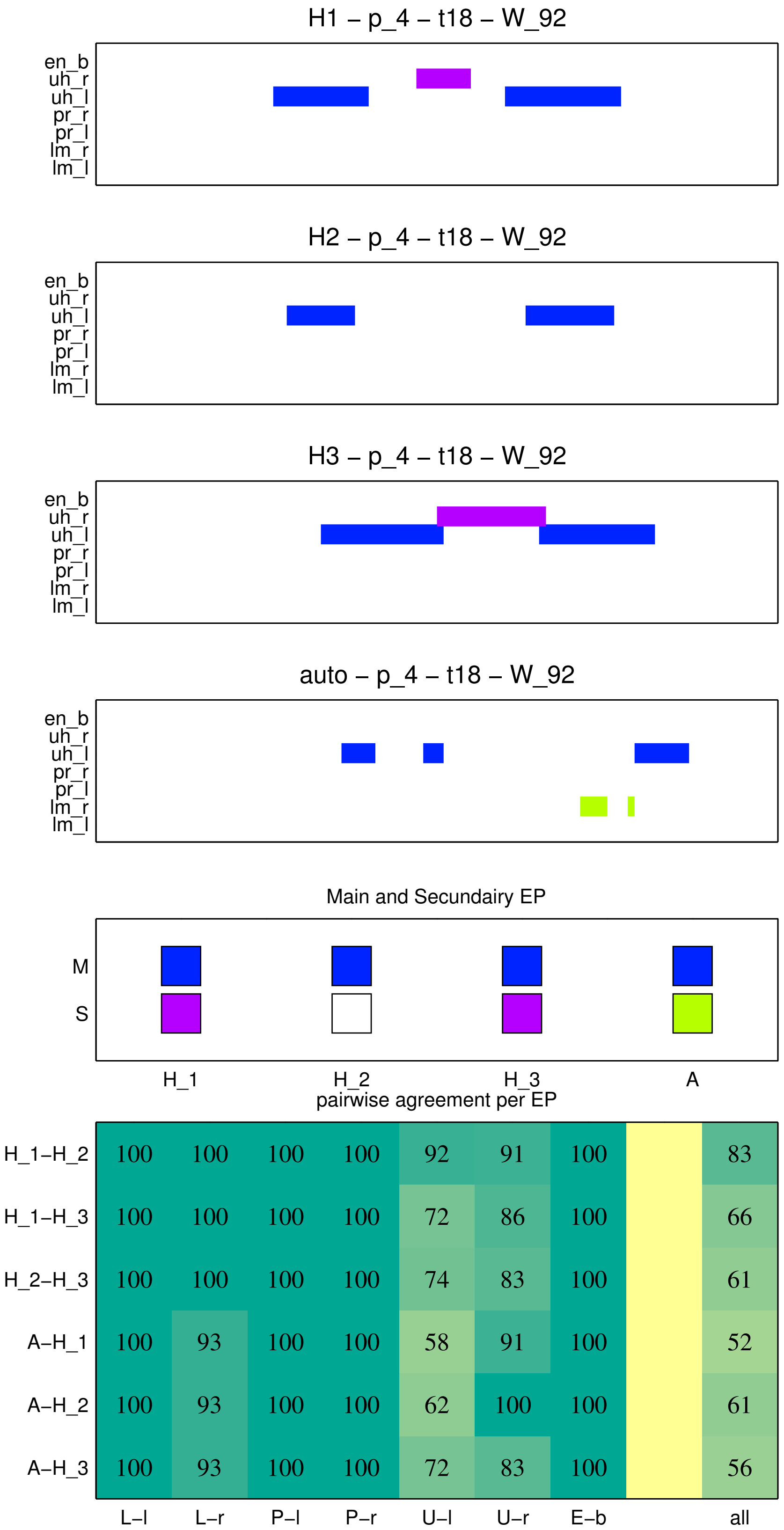
H1 – p_4 – t18 – W_92
H2 – p_4 – t18 – W_92
H3 – p_4 – t18 – W_92
auto – p_4 – t18 – W_92
en_b
uh_r
uh_l
pr_r
pr_l
lm_r
lm_l
Main and Secundairy EP
M
S
H_1
H_2
H_3
A
pairwise agreement per EP

	L–l	L–r	P–l	P–r	U–l	U–r	E–b		all
H_1–H_2	100	100	100	100	92	91	100		83
H_1–H_3	100	100	100	100	72	86	100		66
H_2–H_3	100	100	100	100	74	83	100		61
A–H_1	100	93	100	100	58	91	100		52
A–H_2	100	93	100	100	62	100	100		61
A–H_3	100	93	100	100	72	83	100		56

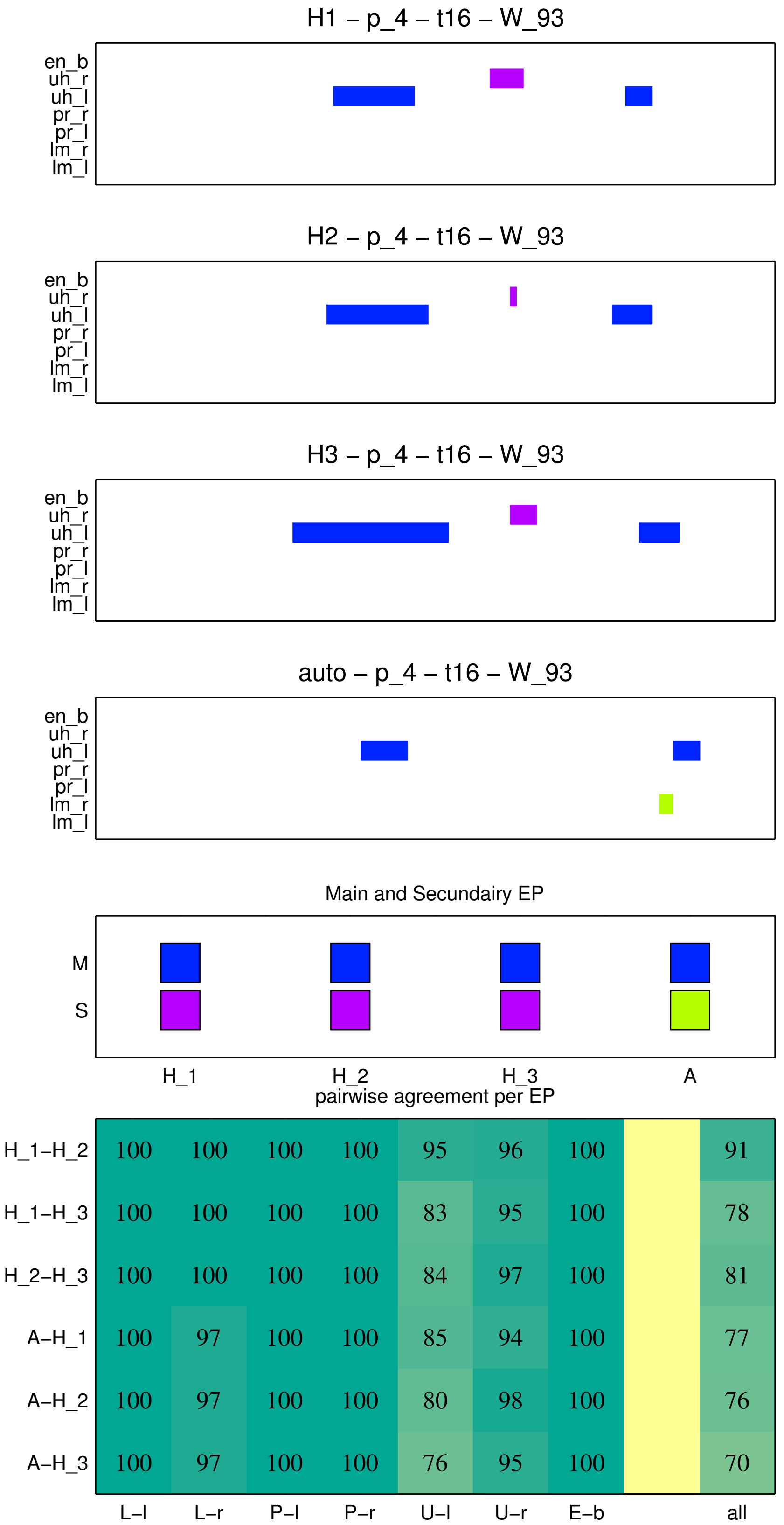
H1 – p_4 – t16 – W_93
H2 – p_4 – t16 – W_93
H3 – p_4 – t16 – W_93
auto – p_4 – t16 – W_93
en_b
uh_r
uh_l
pr_r
pr_l
lm_r
lm_l
Main and Secundairy EP
M
S
H_1
H_2
H_3
A
pairwise agreement per EP

	L–l	L–r	P–l	P–r	U–l	U–r	E–b		all
H_1–H_2	100	100	100	100	95	96	100		91
H_1–H_3	100	100	100	100	83	95	100		78
H_2–H_3	100	100	100	100	84	97	100		81
A–H_1	100	97	100	100	85	94	100		77
A–H_2	100	97	100	100	80	98	100		76
A–H_3	100	97	100	100	76	95	100		70

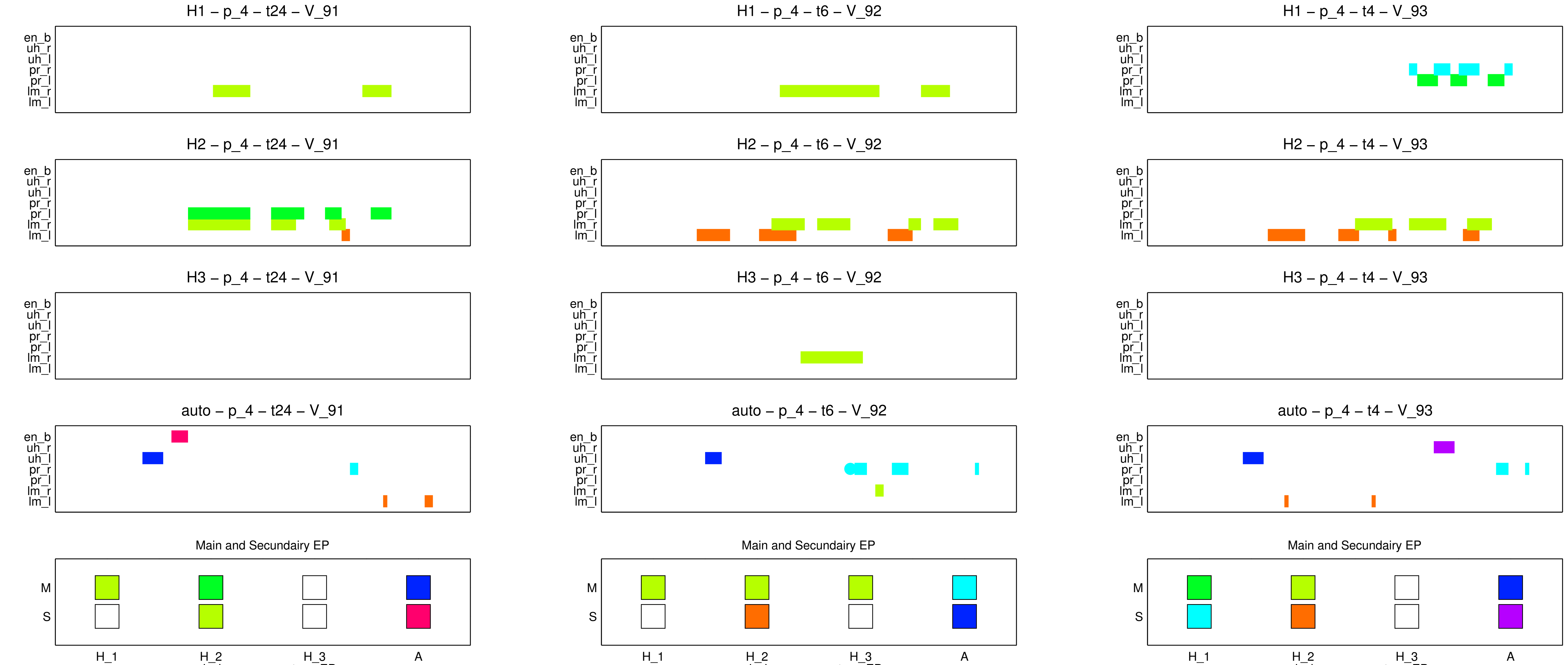

pairwise agreement per EP

	L–l	L–r	P–l	P–r	U–l	U–r	E–b		all
H_1–H_2	97	74	64	100	100	100	100		60
H_1–H_3	100	82	100	100	100	100	100		82
H_2–H_3	97	72	64	100	100	100	100		62
A–H_1	95	82	100	97	94	100	95		65
A–H_2	92	72	64	97	94	100	95		47
A–H_3	95	100	100	97	94	100	95		81

pairwise agreement per EP

	L–l	L–r	P–l	P–r	U–l	U–r	E–b		all
H_1–H_2	74	82	100	100	100	100	100		60
H_1–H_3	100	83	100	100	100	100	100		83
H_2–H_3	74	77	100	100	100	100	100		60
A–H_1	100	68	100	88	95	100	100		56
A–H_2	74	68	100	88	95	100	100		45
A–H_3	100	81	100	88	95	100	100		68

pairwise agreement per EP

	L–l	L–r	P–l	P–r	U–l	U–r	E–b		all
H_1–H_2	76	73	84	83	100	100	100		49
H_1–H_3	100	100	84	83	100	100	100		75
H_2–H_3	76	73	100	100	100	100	100		57
A–H_1	96	100	84	81	94	94	100		64
A–H_2	76	73	100	94	94	94	100		45
A–H_3	96	100	100	94	94	94	100		78

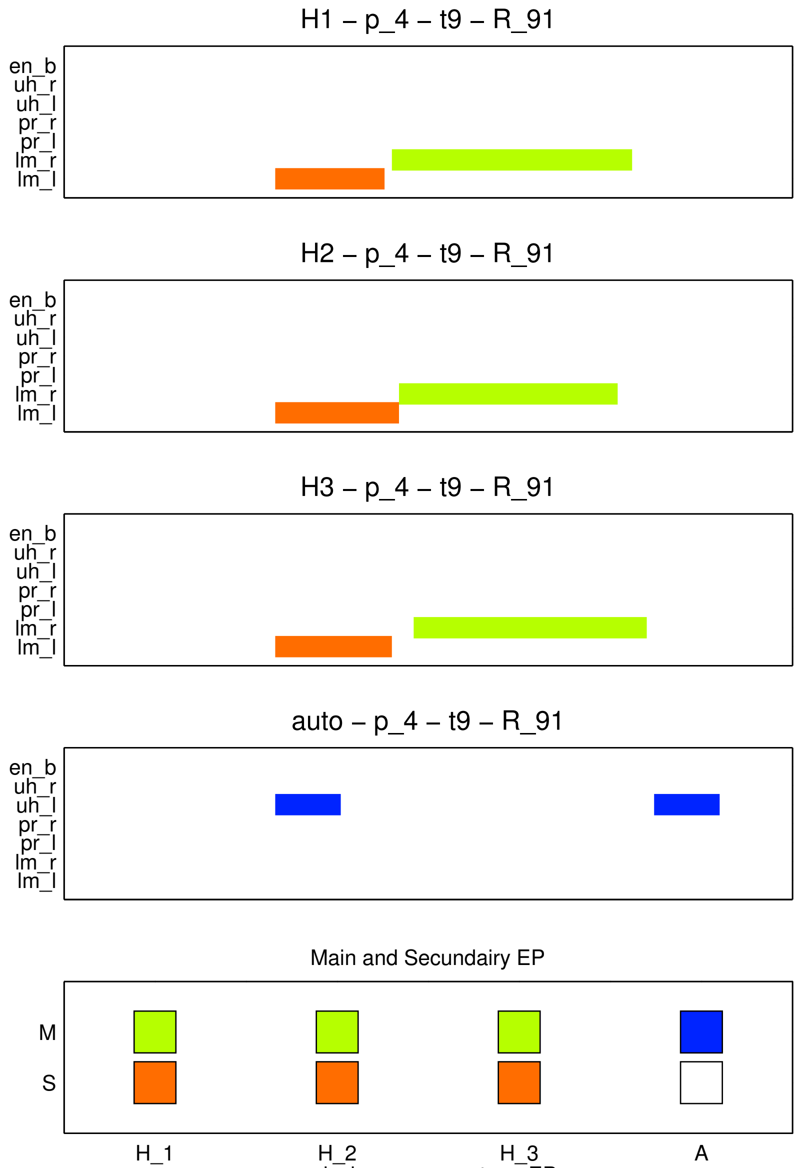

pairwise agreement per EP

	L–l	L–r	P–l	P–r	U–l	U–r	E–b		all
H_1–H_2	98	97	100	100	100	100	100		96
H_1–H_3	99	95	100	100	100	100	100		95
H_2–H_3	99	94	100	100	100	100	100		94
A–H_1	84	66	100	100	80	100	100		40
A–H_2	82	69	100	100	80	100	100		42
A–H_3	83	67	100	100	80	100	100		40

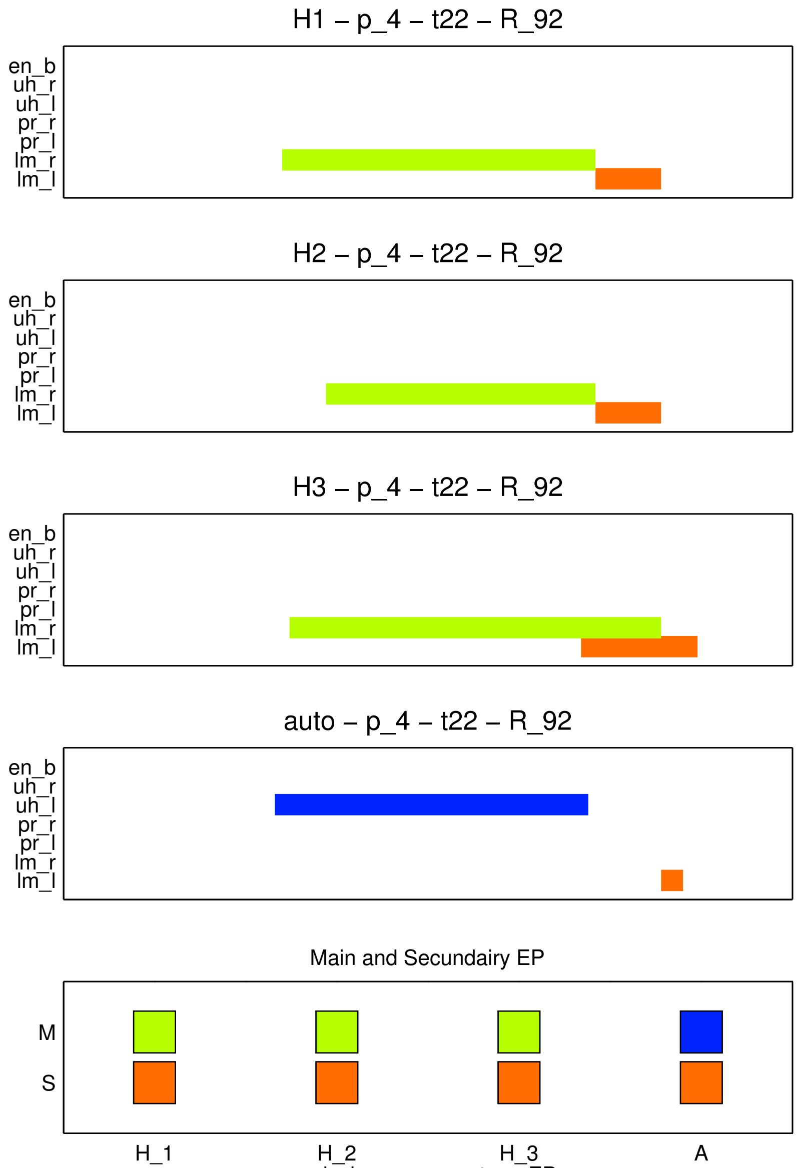

pairwise agreement per EP

	L–l	L–r	P–l	P–r	U–l	U–r	E–b		all
H_1–H_2	100	94	100	100	100	100	100		94
H_1–H_3	93	90	100	100	100	100	100		83
H_2–H_3	93	86	100	100	100	100	100		79
A–H_1	88	56	100	100	56	100	100		44
A–H_2	88	62	100	100	56	100	100		44
A–H_3	87	48	100	100	56	100	100		44

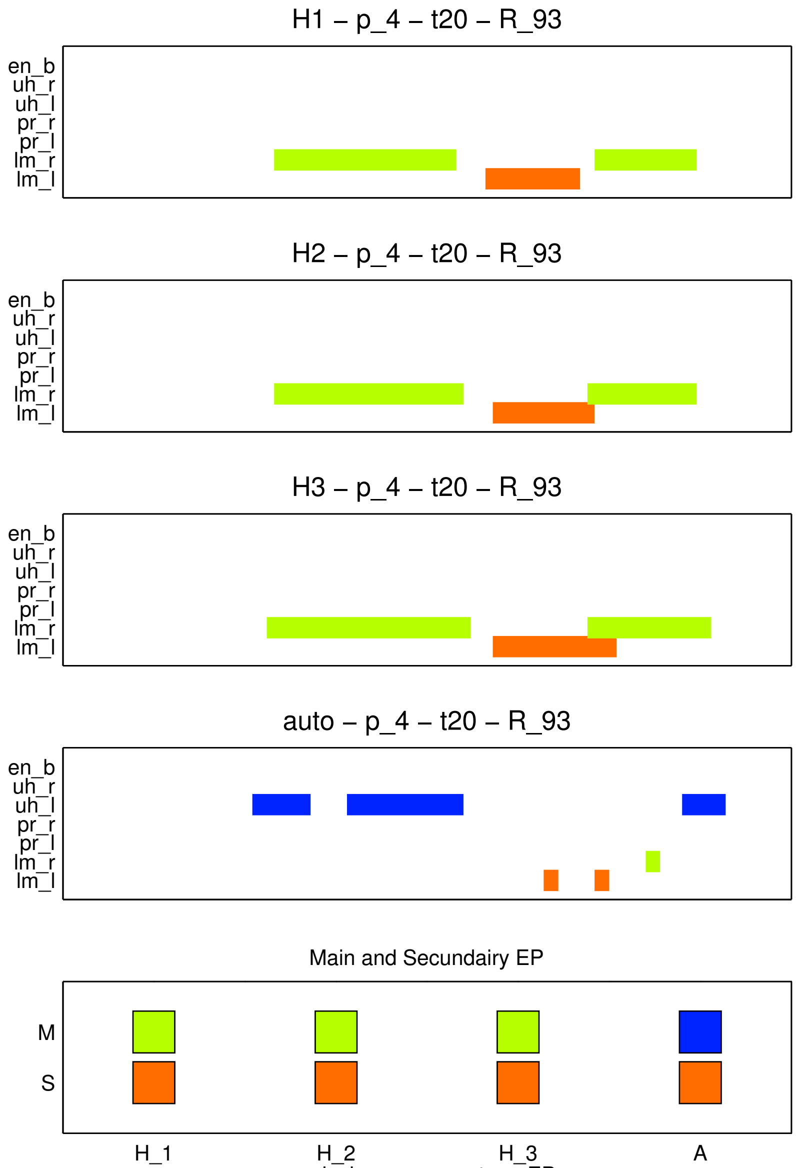

pairwise agreement per EP

	L–l	L–r	P–l	P–r	U–l	U–r	E–b		all
H_1–H_2	97	98	100	100	100	100	100		96
H_1–H_3	94	94	100	100	100	100	100		89
H_2–H_3	97	96	100	100	100	100	100		93
A–H_1	86	62	100	100	67	100	100		43
A–H_2	87	60	100	100	67	100	100		43
A–H_3	88	56	100	100	67	100	100		42

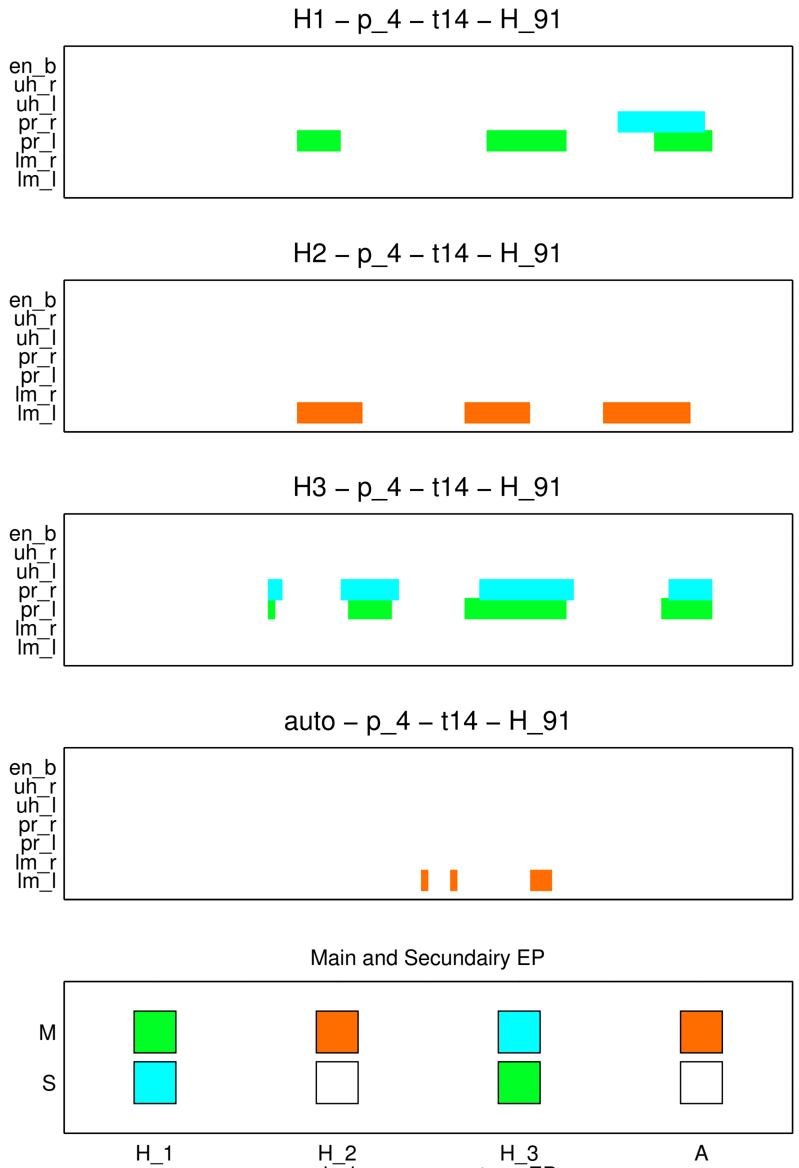

pairwise agreement per EP

	L–l	L–r	P–l	P–r	U–l	U–r	E–b		all
H_1–H_2	67	100	72	87	100	100	100		59
H_1–H_3	100	100	80	66	100	100	100		58
H_2–H_3	67	100	68	67	100	100	100		50
A–H_1	92	100	72	87	100	100	100		63
A–H_2	61	100	100	100	100	100	100		61
A–H_3	92	100	68	67	100	100	100		60

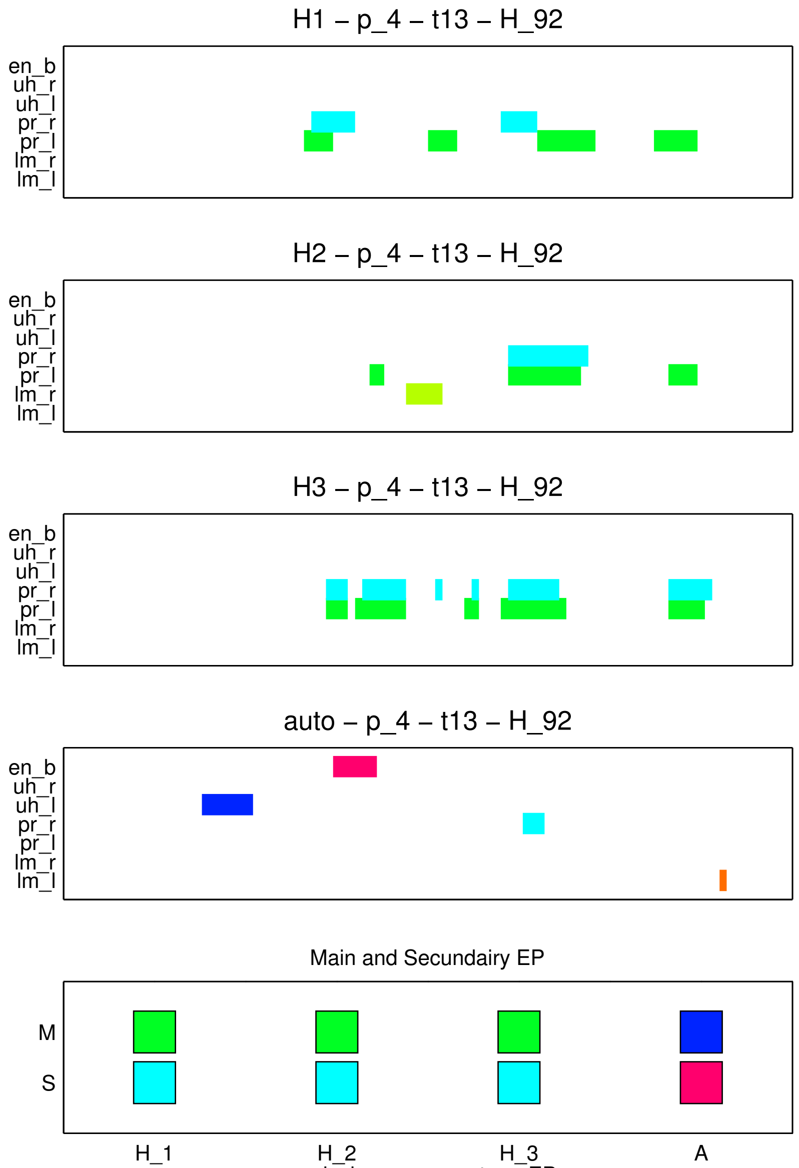

pairwise agreement per EP

	L–l	L–r	P–l	P–r	U–l	U–r	E–b		all
H_1–H_2	100	94	79	85	100	100	100		66
H_1–H_3	100	100	67	75	100	100	100		58
H_2–H_3	100	94	84	74	100	100	100		68
A–H_1	98	100	74	89	92	100	93		55
A–H_2	98	94	81	92	92	100	93		59
A–H_3	98	100	69	74	92	100	93		56

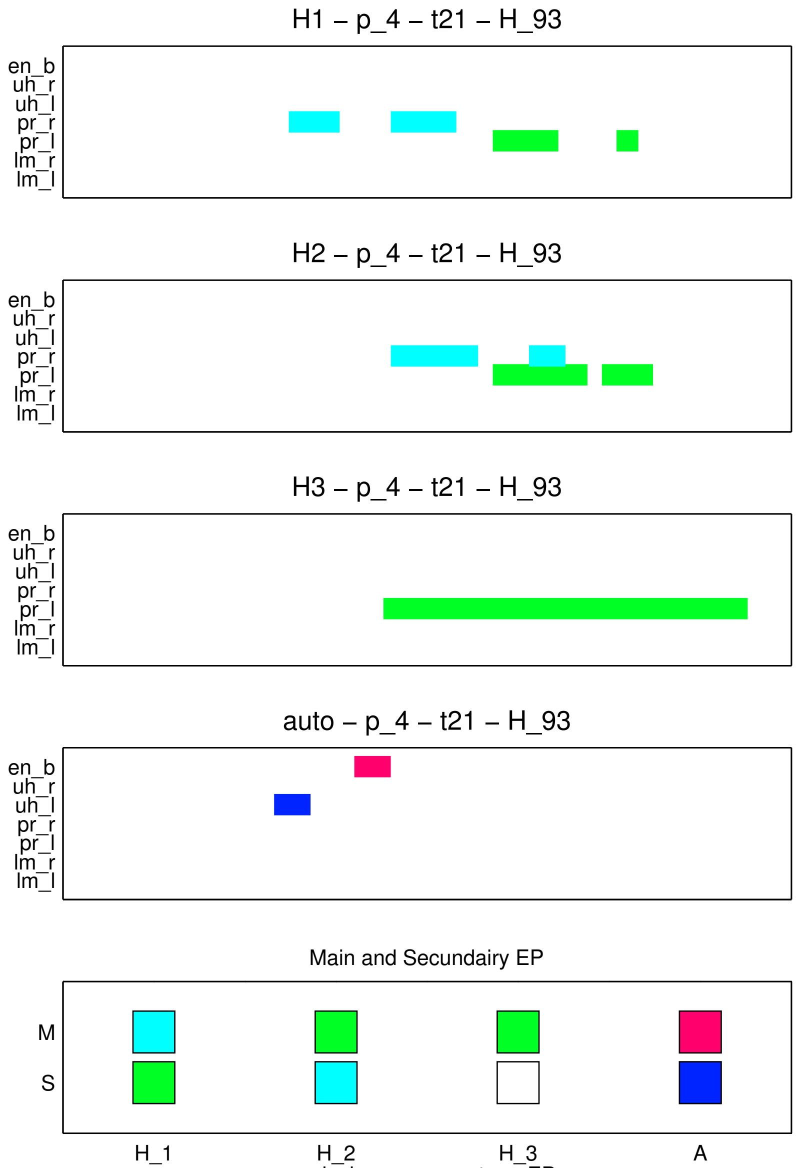

pairwise agreement per EP

	L–l	L–r	P–l	P–r	U–l	U–r	E–b		all
H_1–H_2	100	100	92	83	100	100	100		76
H_1–H_3	100	100	63	82	100	100	100		55
H_2–H_3	100	100	71	81	100	100	100		65
A–H_1	100	100	86	82	94	100	94		61
A–H_2	100	100	78	81	94	100	94		54
A–H_3	100	100	49	100	94	100	94		39
