# Supplementary material for: Haptic Exploratory Behavior During Object Discrimination: A Novel Automatic Annotation Method
Source: PLoS One. 2015 Feb 6;10(2):e0117017. doi: 10.1371/journal.pone.0117017 (PMC4319767; doi:10.1371/journal.pone.0117017)
Supplement: S2 Annotation Output — (PDF) [file pone.0117017.s002.pdf]

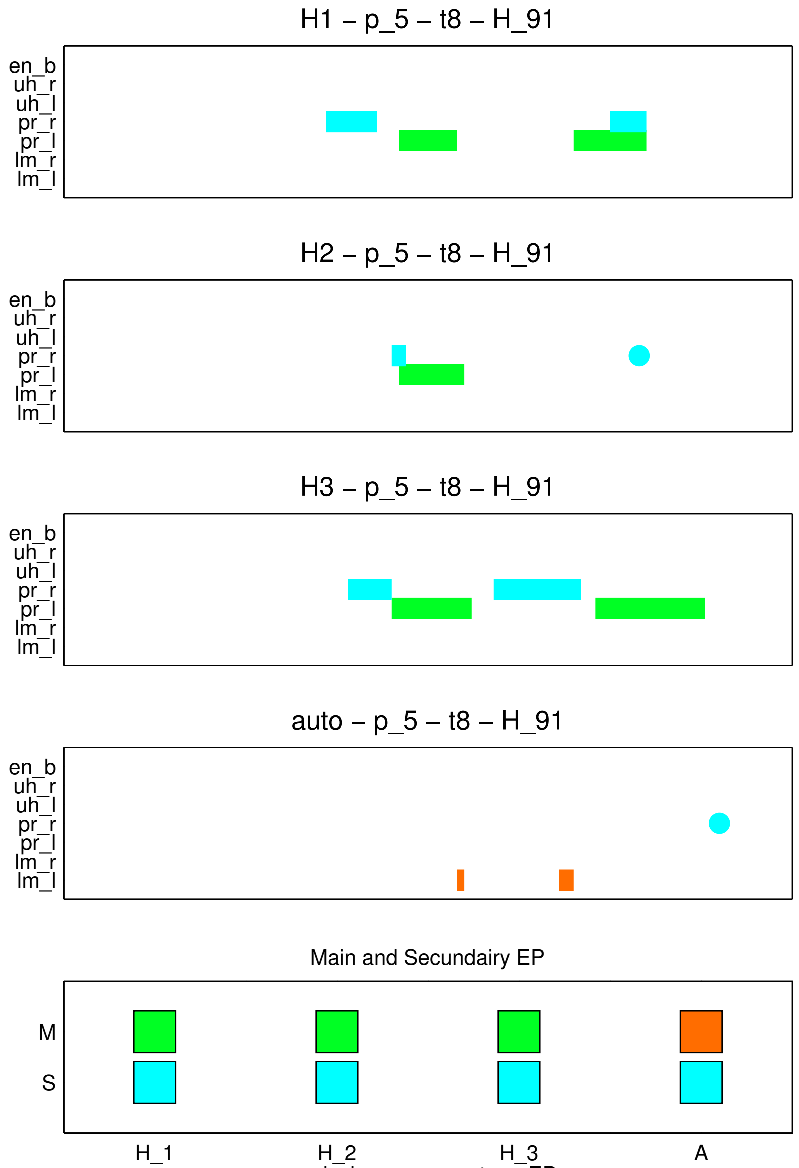

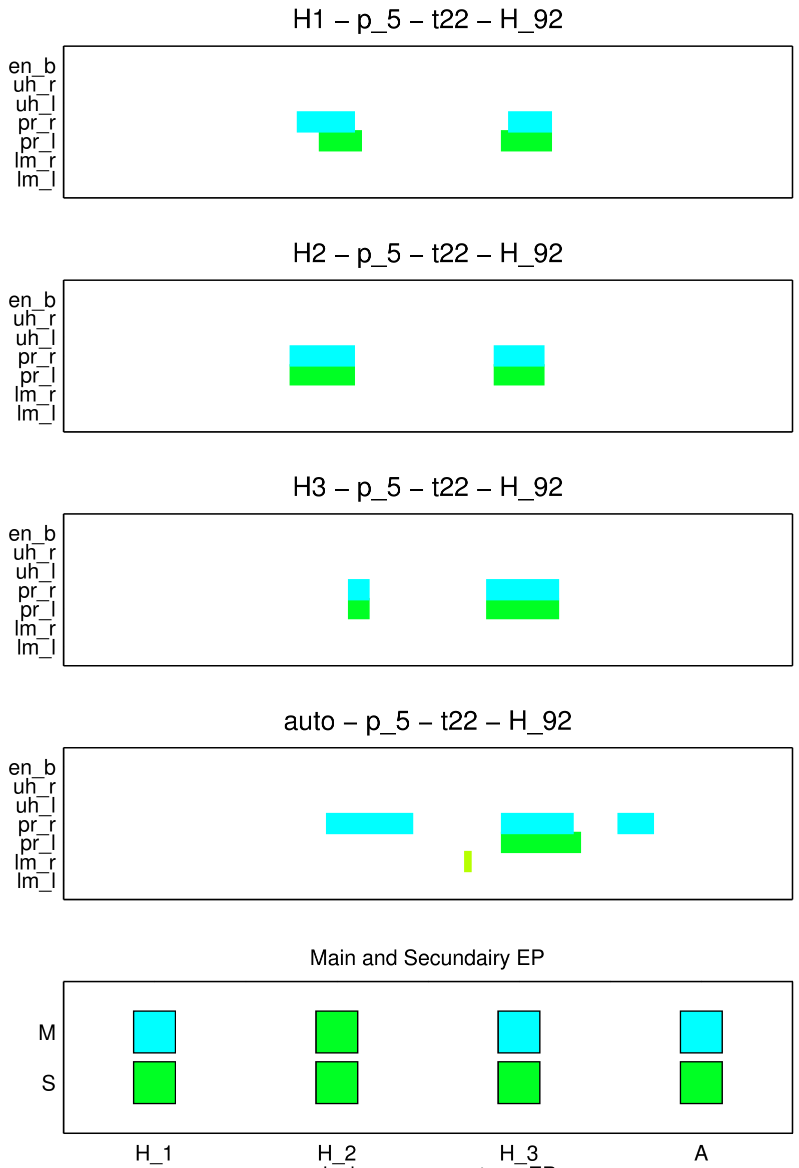

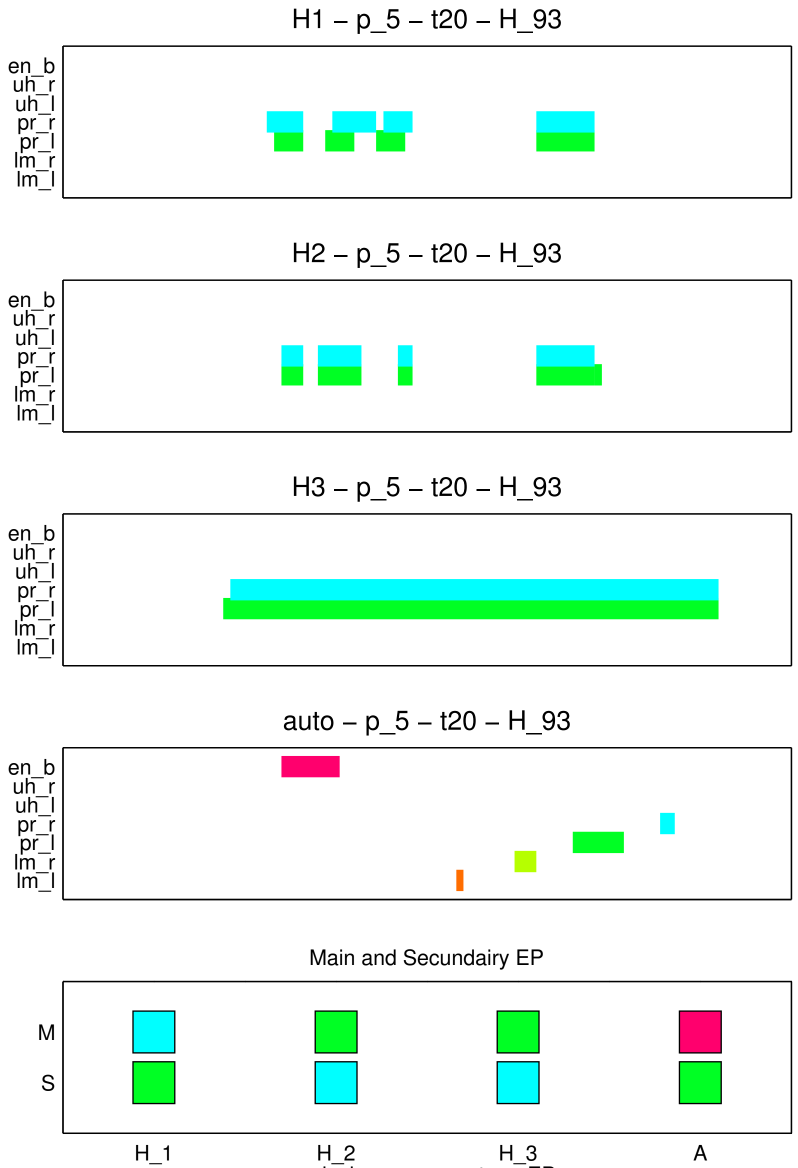

pairwise agreement per EP

	L–l	L–r	P–l	P–r	U–l	U–r	E–b		all
H_1–H_2	100	100	88	84	100	100	100		77
H_1–H_3	100	100	86	76	100	100	100		65
H_2–H_3	100	100	82	78	100	100	100		61
A–H_1	95	100	80	85	100	100	100		68
A–H_2	95	100	90	95	100	100	100		84
A–H_3	95	100	72	79	100	100	100		52

pairwise agreement per EP

	L–l	L–r	P–l	P–r	U–l	U–r	E–b		all
H_1–H_2	100	100	93	96	100	100	100		92
H_1–H_3	100	100	92	87	100	100	100		87
H_2–H_3	100	100	87	87	100	100	100		87
A–H_1	100	98	89	78	100	100	100		70
A–H_2	100	98	84	76	100	100	100		68
A–H_3	100	98	91	81	100	100	100		74

pairwise agreement per EP

	L–l	L–r	P–l	P–r	U–l	U–r	E–b		all
H_1–H_2	100	100	92	92	100	100	100		89
H_1–H_3	100	100	55	59	100	100	100		54
H_2–H_3	100	100	55	55	100	100	100		54
A–H_1	98	96	76	70	100	100	91		58
A–H_2	98	96	78	74	100	100	91		65
A–H_3	98	96	39	35	100	100	91		31

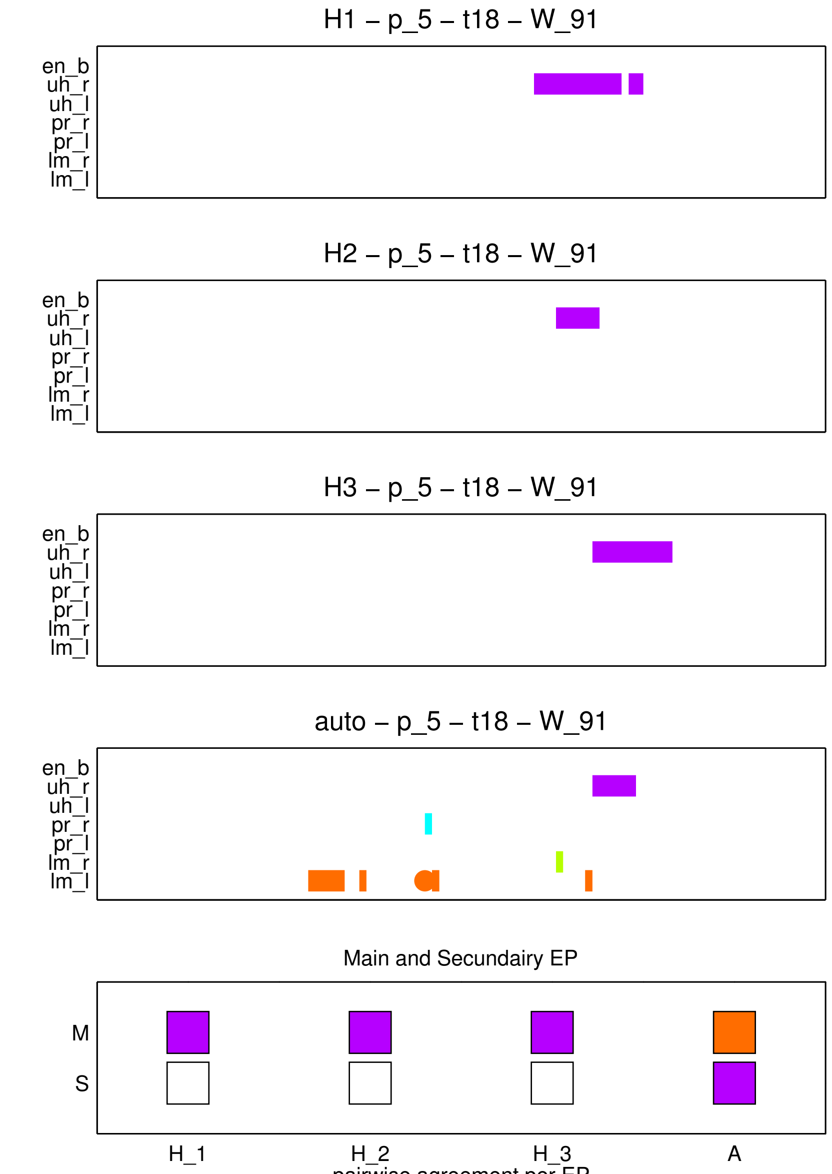

pairwise agreement per EP

	L–l	L–r	P–l	P–r	U–l	U–r	E–b		all
H_1–H_2	100	100	100	100	100	91	100		91
H_1–H_3	100	100	100	100	100	88	100		88
H_2–H_3	100	100	100	100	100	85	100		85
A–H_1	87	98	100	98	100	91	100		79
A–H_2	87	98	100	98	100	90	100		78
A–H_3	87	98	100	98	100	95	100		80

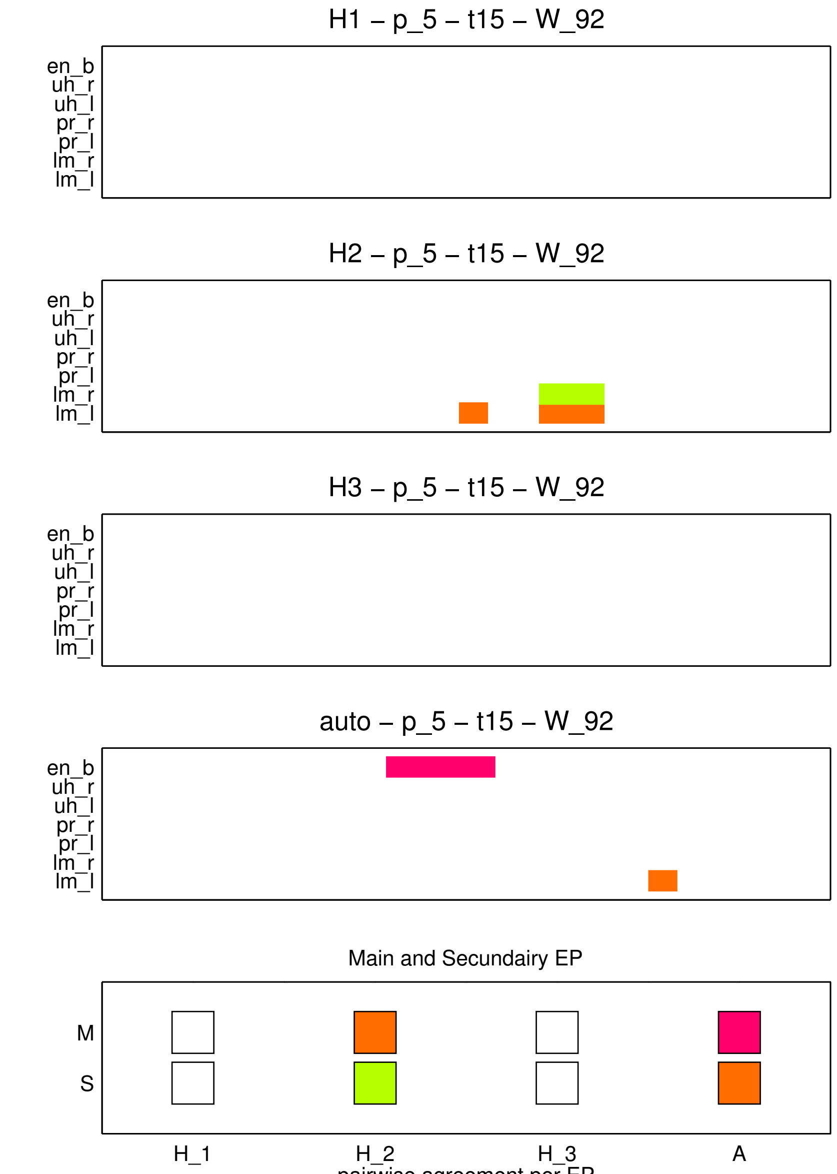

pairwise agreement per EP

	L–l	L–r	P–l	P–r	U–l	U–r	E–b		all
H_1–H_2	85	90	100	100	100	100	100		85
H_1–H_3	100	100	100	100	100	100	100		100
H_2–H_3	85	90	100	100	100	100	100		85
A–H_1	95	100	100	100	100	100	84		79
A–H_2	80	90	100	100	100	100	84		69
A–H_3	95	100	100	100	100	100	84		79

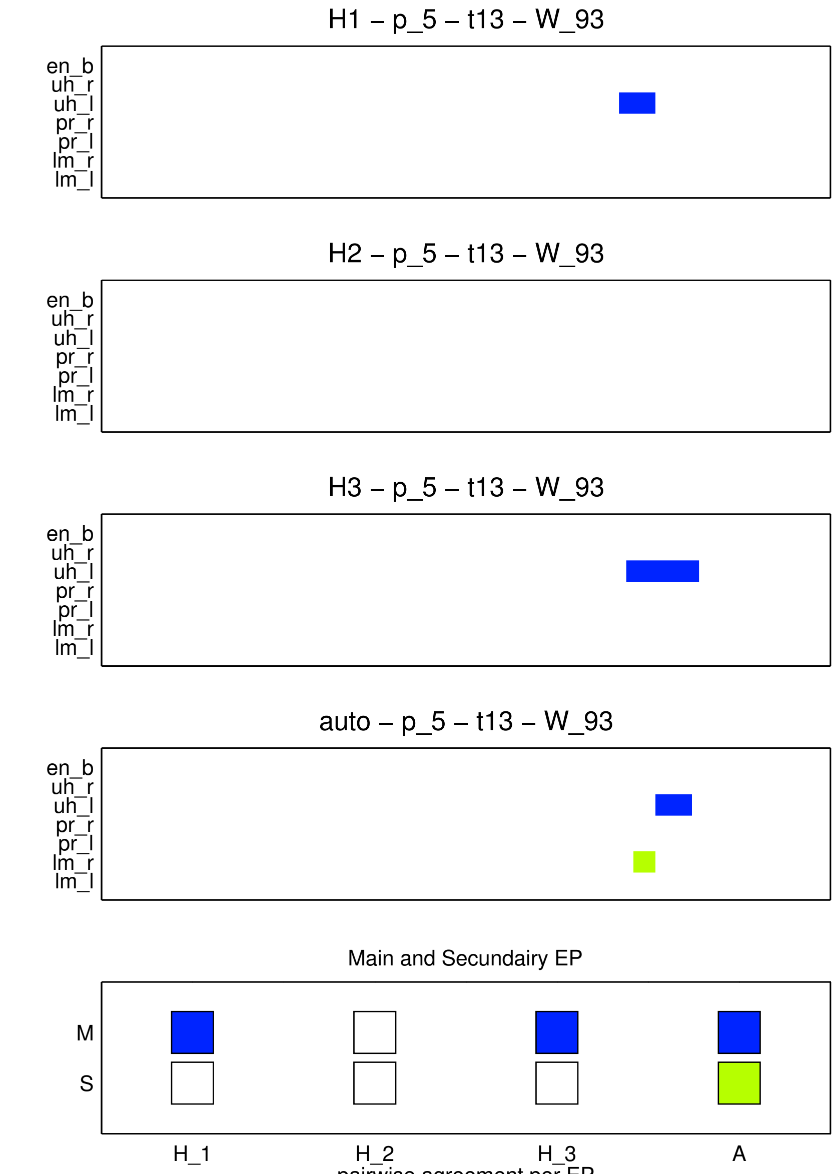

pairwise agreement per EP

	L–l	L–r	P–l	P–r	U–l	U–r	E–b		all
H_1–H_2	100	100	100	100	94	100	100		94
H_1–H_3	100	100	100	100	93	100	100		93
H_2–H_3	100	100	100	100	89	100	100		89
A–H_1	100	96	100	100	90	100	100		89
A–H_2	100	96	100	100	94	100	100		91
A–H_3	100	96	100	100	95	100	100		94

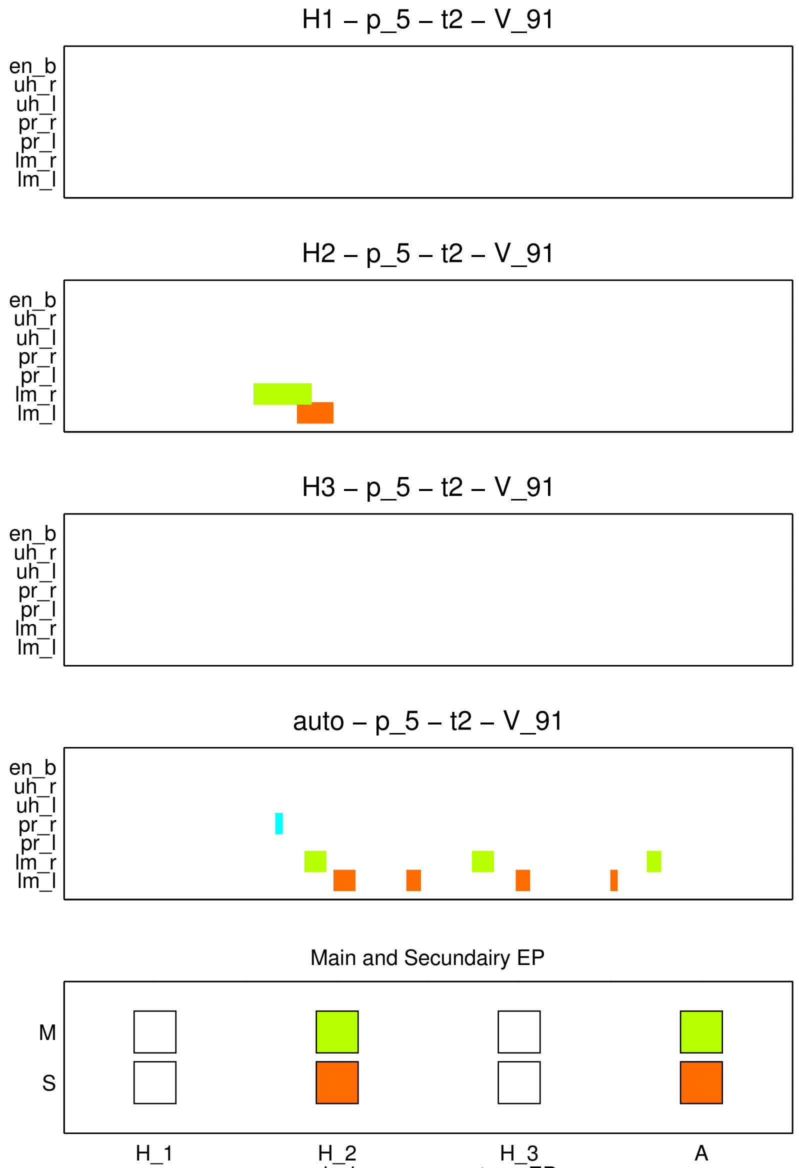

pairwise agreement per EP

	L–l	L–r	P–l	P–r	U–l	U–r	E–b		all
H_1–H_2	94	91	100	100	100	100	100		88
H_1–H_3	100	100	100	100	100	100	100		100
H_2–H_3	94	91	100	100	100	100	100		88
A–H_1	88	89	100	98	100	100	100		75
A–H_2	84	84	100	98	100	100	100		71
A–H_3	88	89	100	98	100	100	100		75

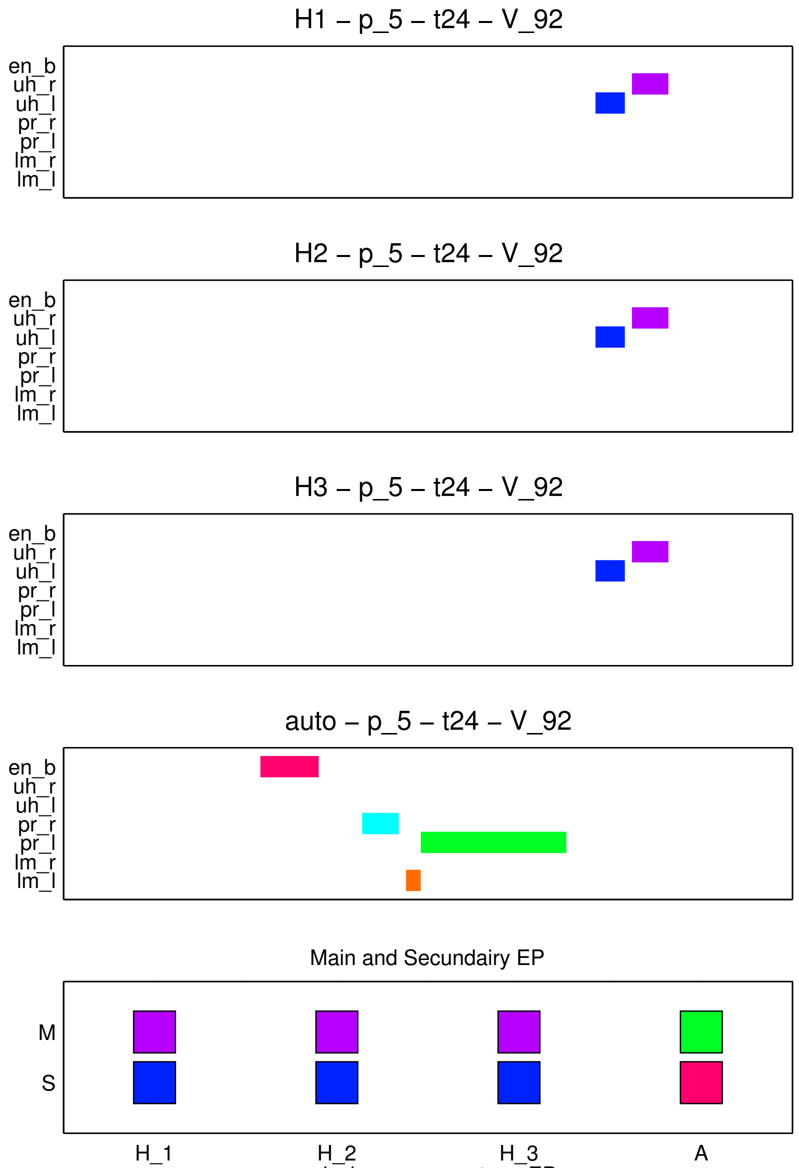

pairwise agreement per EP

	L–l	L–r	P–l	P–r	U–l	U–r	E–b		all
H_1–H_2	100	100	100	100	100	100	100		100
H_1–H_3	100	100	100	100	100	100	100		100
H_2–H_3	100	100	100	100	100	100	100		100
A–H_1	97	100	79	94	95	94	91		51
A–H_2	97	100	79	94	95	94	91		51
A–H_3	97	100	79	94	95	94	91		51

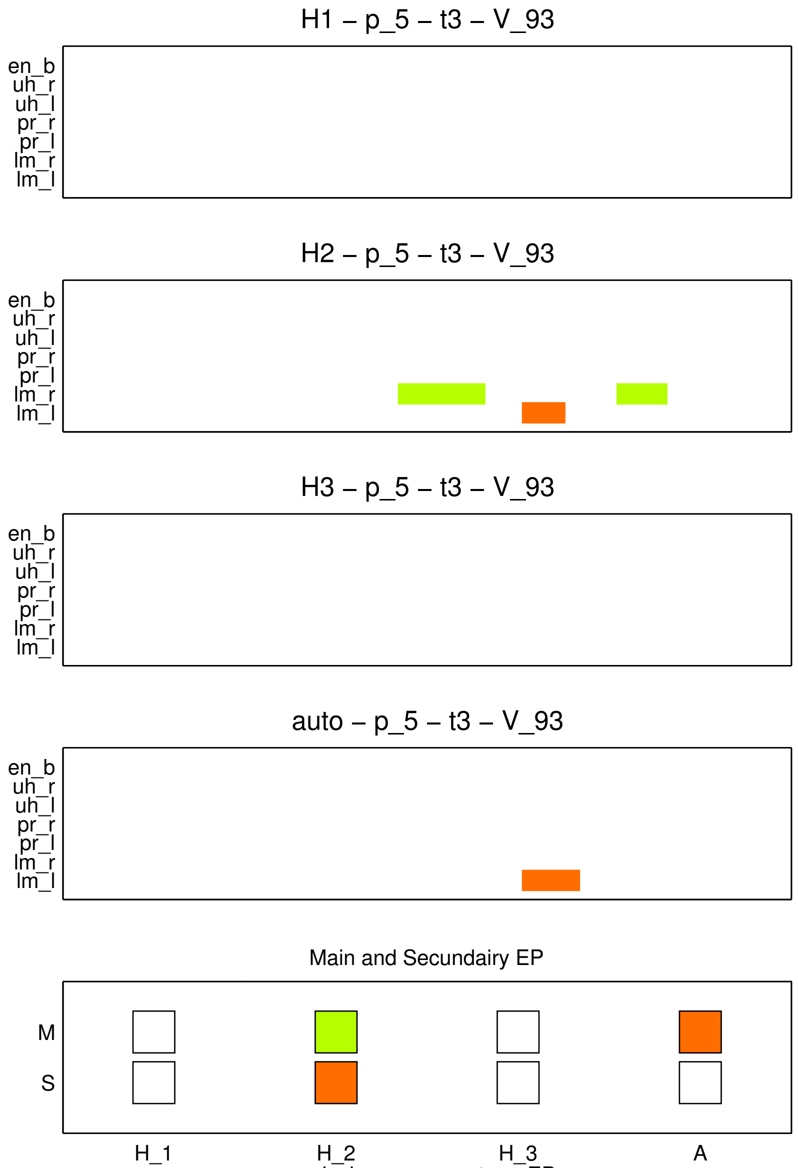

pairwise agreement per EP

	L–l	L–r	P–l	P–r	U–l	U–r	E–b		all
H_1–H_2	93	79	100	100	100	100	100		72
H_1–H_3	100	100	100	100	100	100	100		100
H_2–H_3	93	79	100	100	100	100	100		72
A–H_1	91	100	100	100	100	100	100		91
A–H_2	98	79	100	100	100	100	100		77
A–H_3	91	100	100	100	100	100	100		91

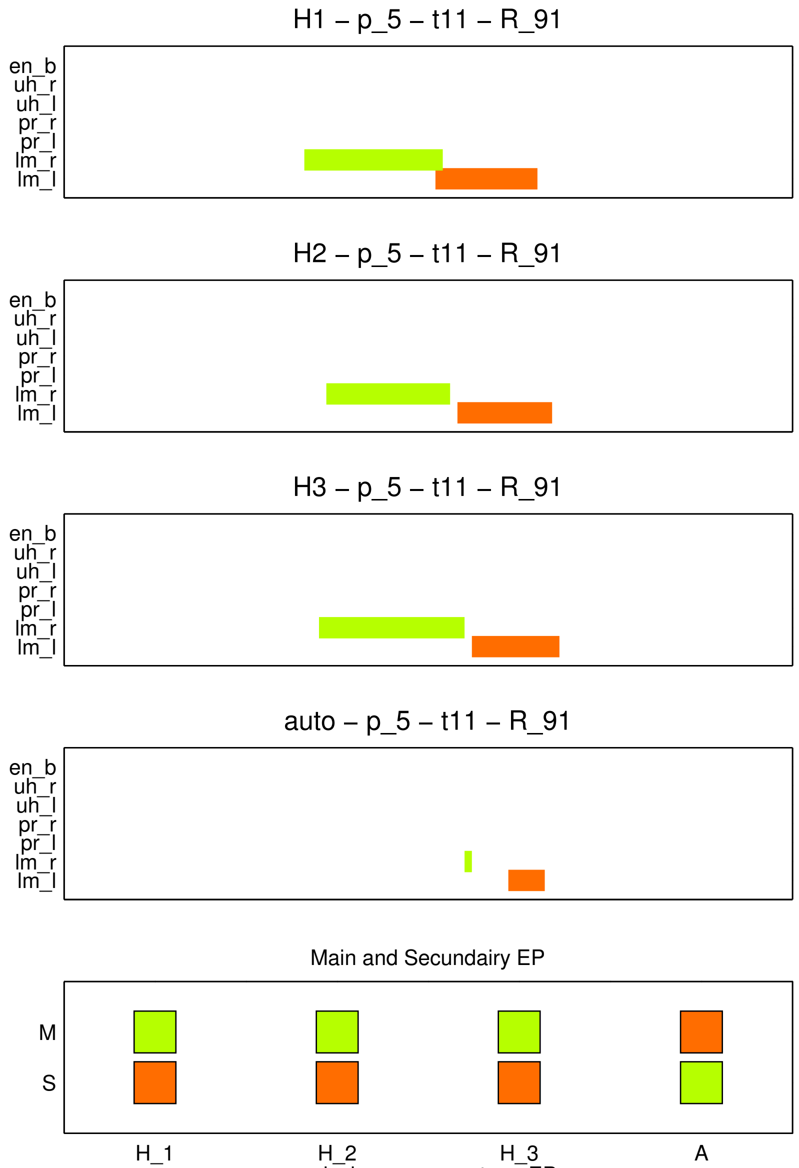

pairwise agreement per EP

	L–l	L–r	P–l	P–r	U–l	U–r	E–b		all
H_1–H_2	95	96	100	100	100	100	100		92
H_1–H_3	92	95	100	100	100	100	100		90
H_2–H_3	97	97	100	100	100	100	100		96
A–H_1	89	78	100	100	100	100	100		71
A–H_2	92	80	100	100	100	100	100		74
A–H_3	93	79	100	100	100	100	100		73

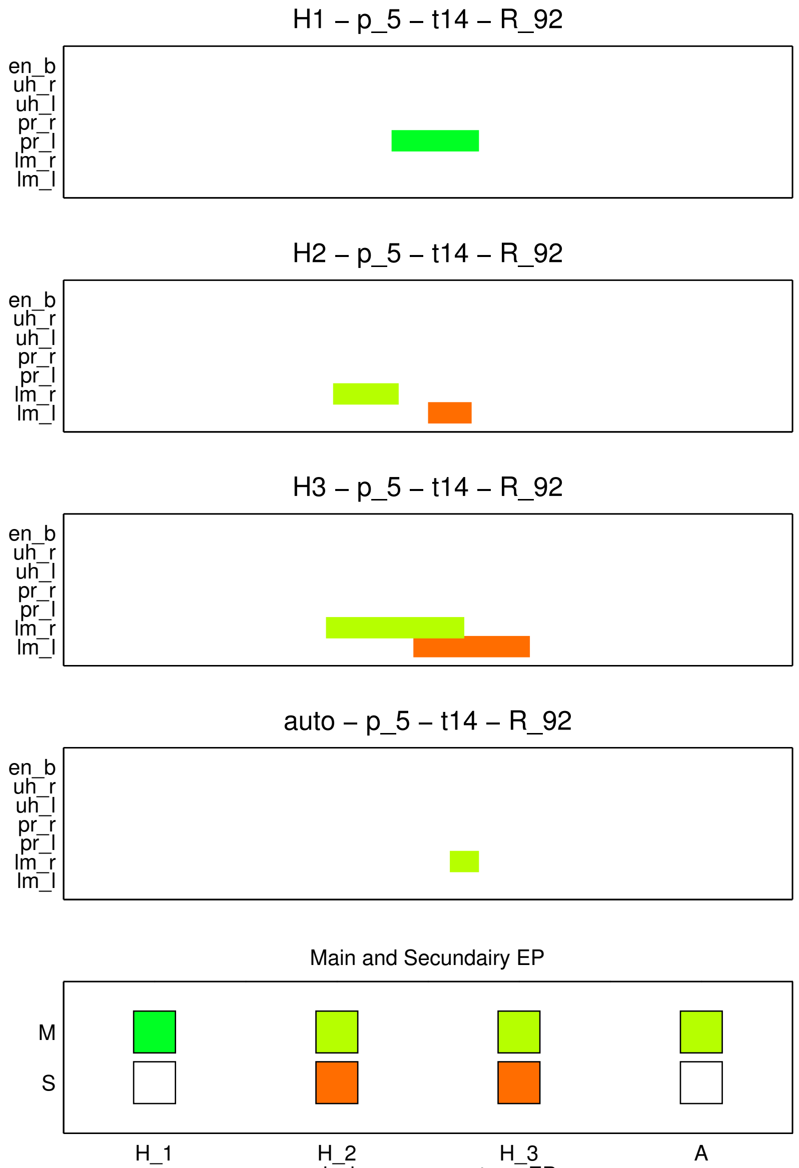

pairwise agreement per EP

	L–l	L–r	P–l	P–r	U–l	U–r	E–b		all
H_1–H_2	93	90	87	100	100	100	100		79
H_1–H_3	83	80	87	100	100	100	100		71
H_2–H_3	90	90	100	100	100	100	100		82
A–H_1	100	95	87	100	100	100	100		87
A–H_2	93	85	100	100	100	100	100		82
A–H_3	83	81	100	100	100	100	100		71

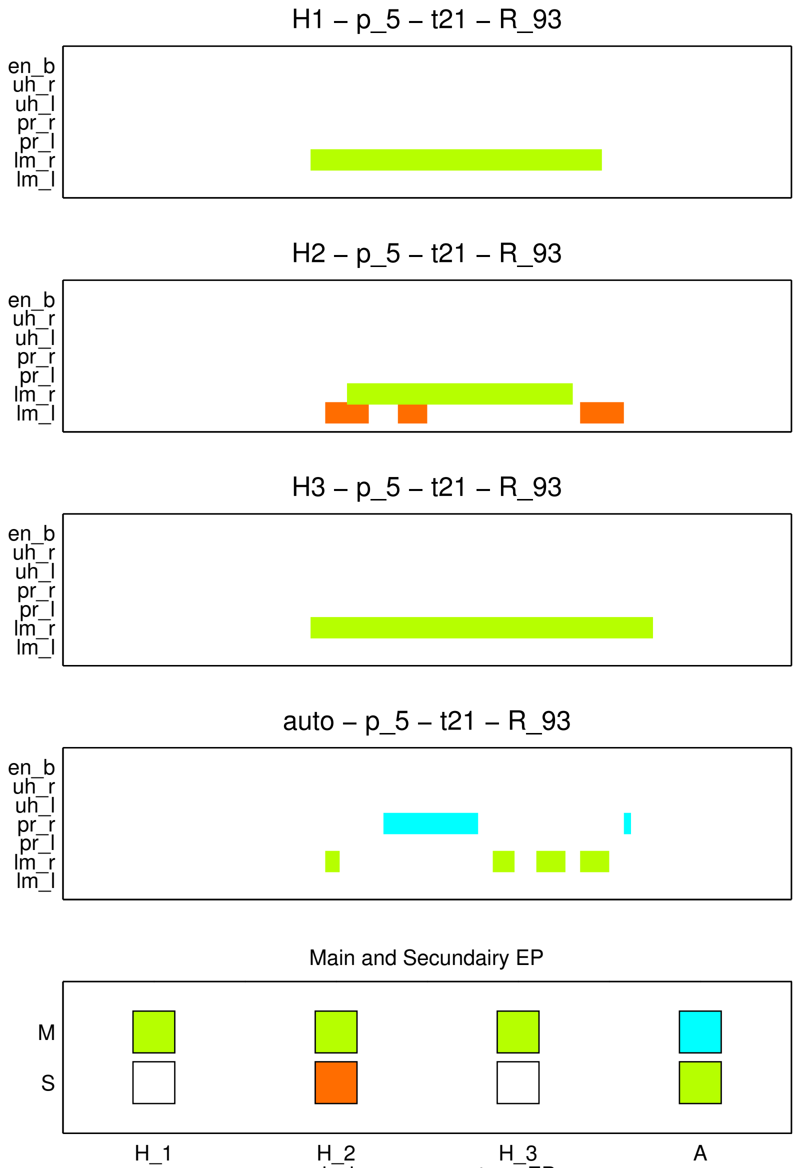

pairwise agreement per EP

	L–l	L–r	P–l	P–r	U–l	U–r	E–b		all
H_1–H_2	81	91	100	100	100	100	100		79
H_1–H_3	100	93	100	100	100	100	100		93
H_2–H_3	81	84	100	100	100	100	100		75
A–H_1	100	74	100	84	100	100	100		72
A–H_2	81	69	100	84	100	100	100		66
A–H_3	100	69	100	84	100	100	100		69
