# Supplementary material for: Haptic Exploratory Behavior During Object Discrimination: A Novel Automatic Annotation Method
Source: PLoS One. 2015 Feb 6;10(2):e0117017. doi: 10.1371/journal.pone.0117017 (PMC4319767; doi:10.1371/journal.pone.0117017)
Supplement: S3 Annotation Output — (PDF) [file pone.0117017.s003.pdf]

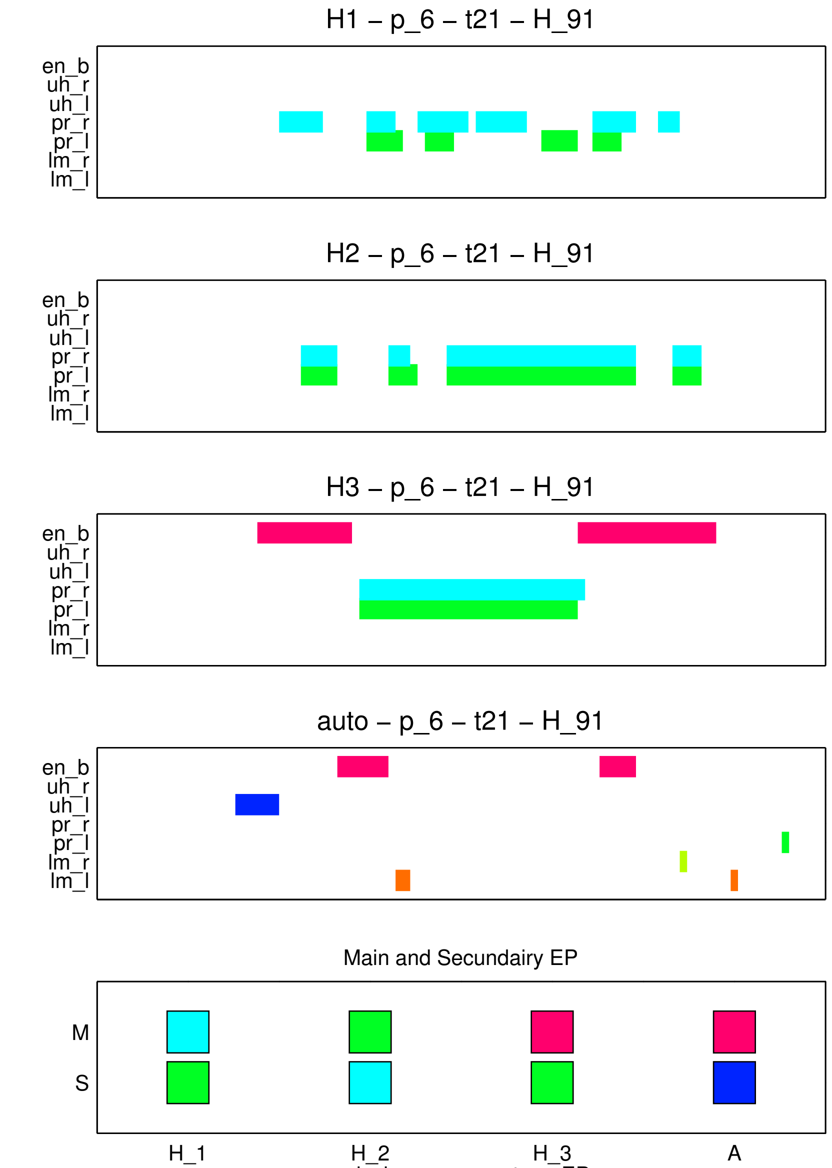

pairwise agreement per EP

	L–l	L–r	P–l	P–r	U–l	U–r	E–b		all
H_1–H_2	100	100	67	73	100	100	100		55
H_1–H_3	100	100	81	71	100	100	66		46
H_2–H_3	100	100	74	74	100	100	66		58
A–H_1	95	98	76	61	93	100	86		38
A–H_2	95	98	55	58	93	100	86		40
A–H_3	95	98	67	68	93	100	70		38

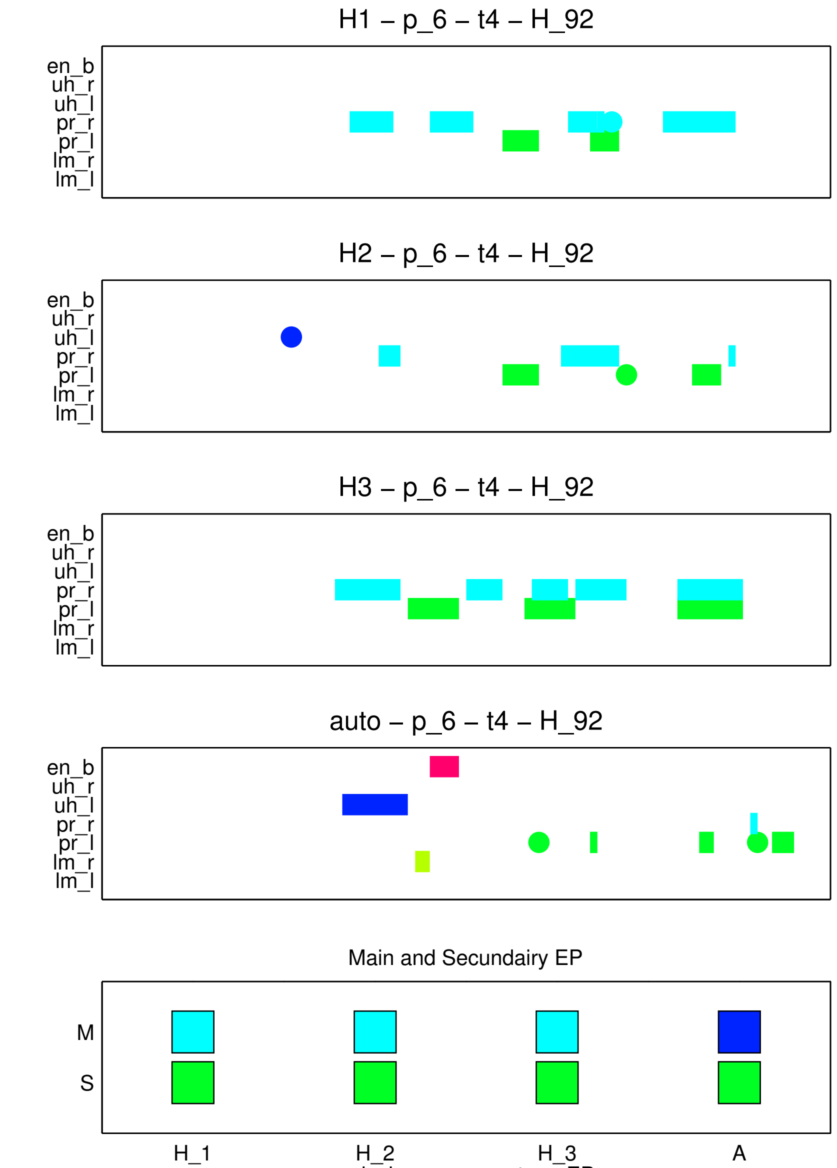

pairwise agreement per EP

	L–l	L–r	P–l	P–r	U–l	U–r	E–b		all
H_1–H_2	100	100	89	77	99	100	100		71
H_1–H_3	100	100	69	78	100	100	100		58
H_2–H_3	100	100	78	75	99	100	100		59
A–H_1	100	97	84	66	90	100	95		51
A–H_2	100	97	85	83	89	100	95		57
A–H_3	100	97	71	58	90	100	95		45

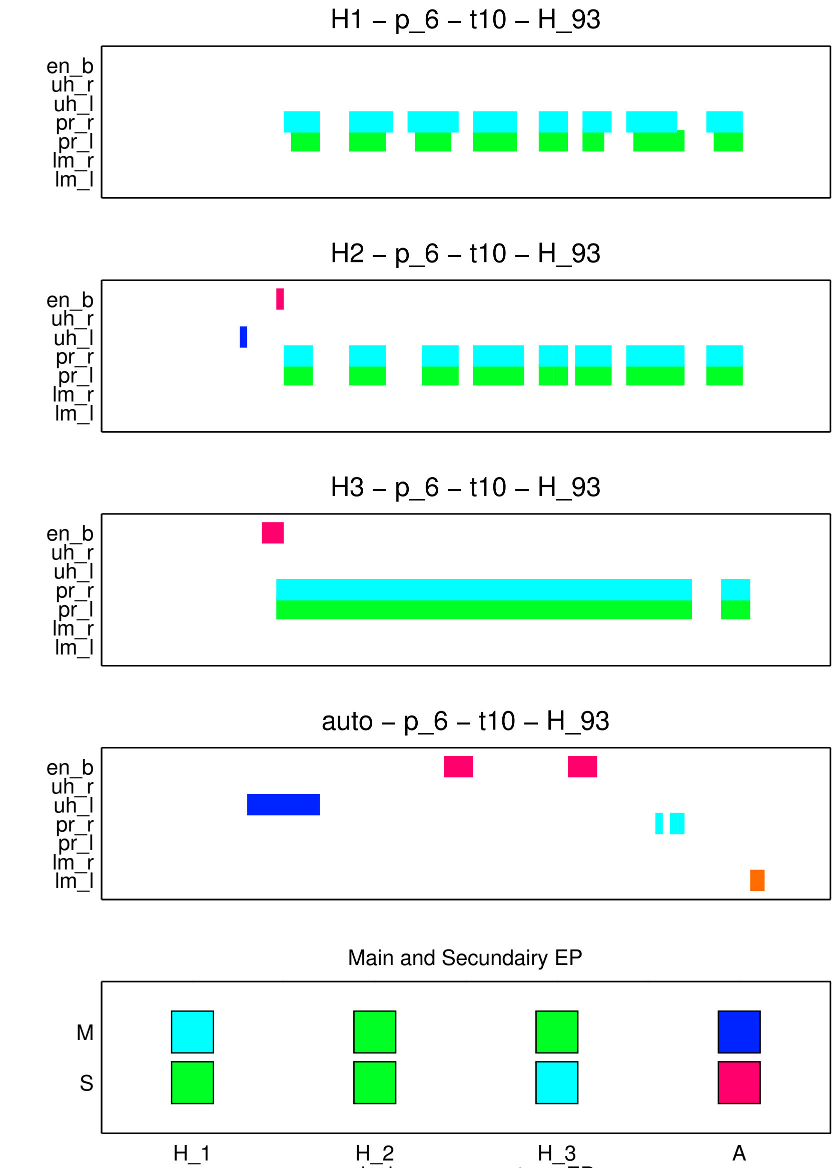

pairwise agreement per EP

	L–l	L–r	P–l	P–r	U–l	U–r	E–b		all
H_1–H_2	100	100	91	93	98	100	98		85
H_1–H_3	100	100	81	85	100	100	96		77
H_2–H_3	100	100	84	84	98	100	98		80
A–H_1	97	100	54	51	89	100	90		37
A–H_2	97	100	49	54	89	100	88		39
A–H_3	97	100	37	42	89	100	86		31

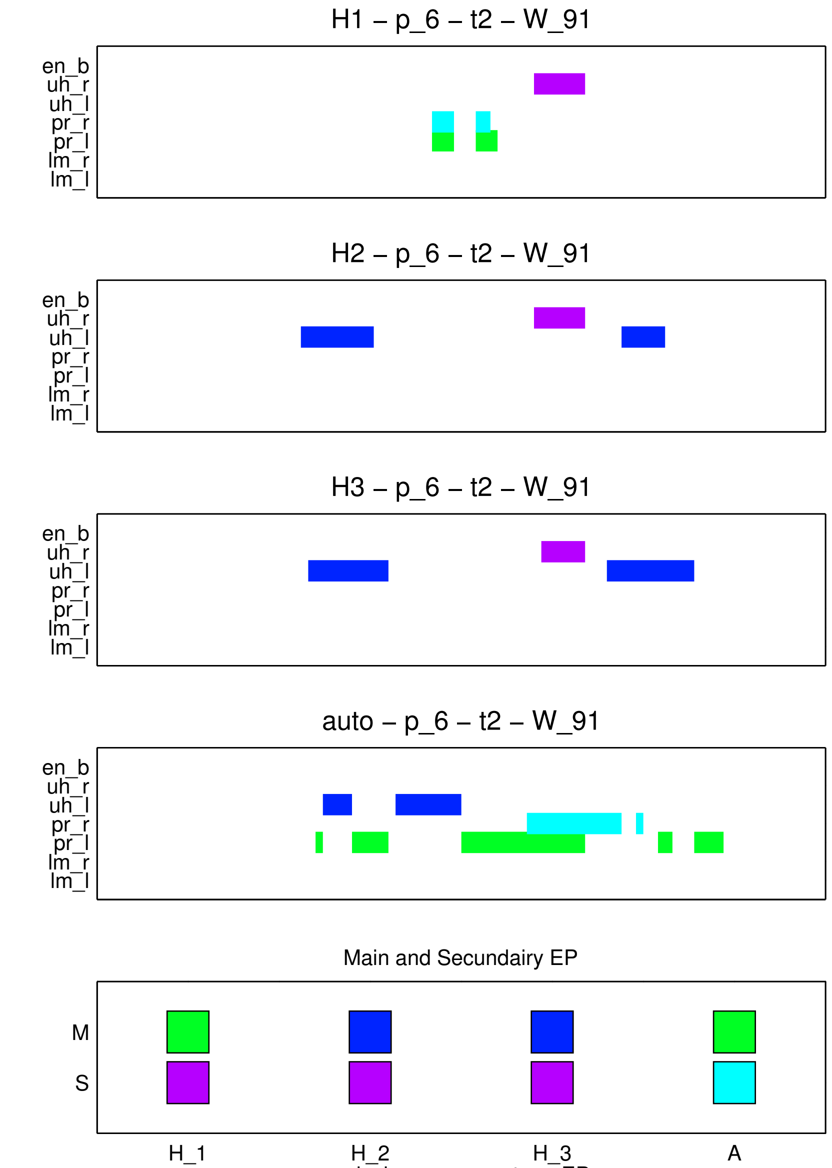

pairwise agreement per EP

	L–l	L–r	P–l	P–r	U–l	U–r	E–b		all
H_1–H_2	100	100	92	93	82	100	100		74
H_1–H_3	100	100	92	93	75	99	100		66
H_2–H_3	100	100	100	100	91	99	100		90
A–H_1	100	100	66	77	85	92	100		48
A–H_2	100	100	66	84	77	92	100		46
A–H_3	100	100	66	84	70	93	100		45

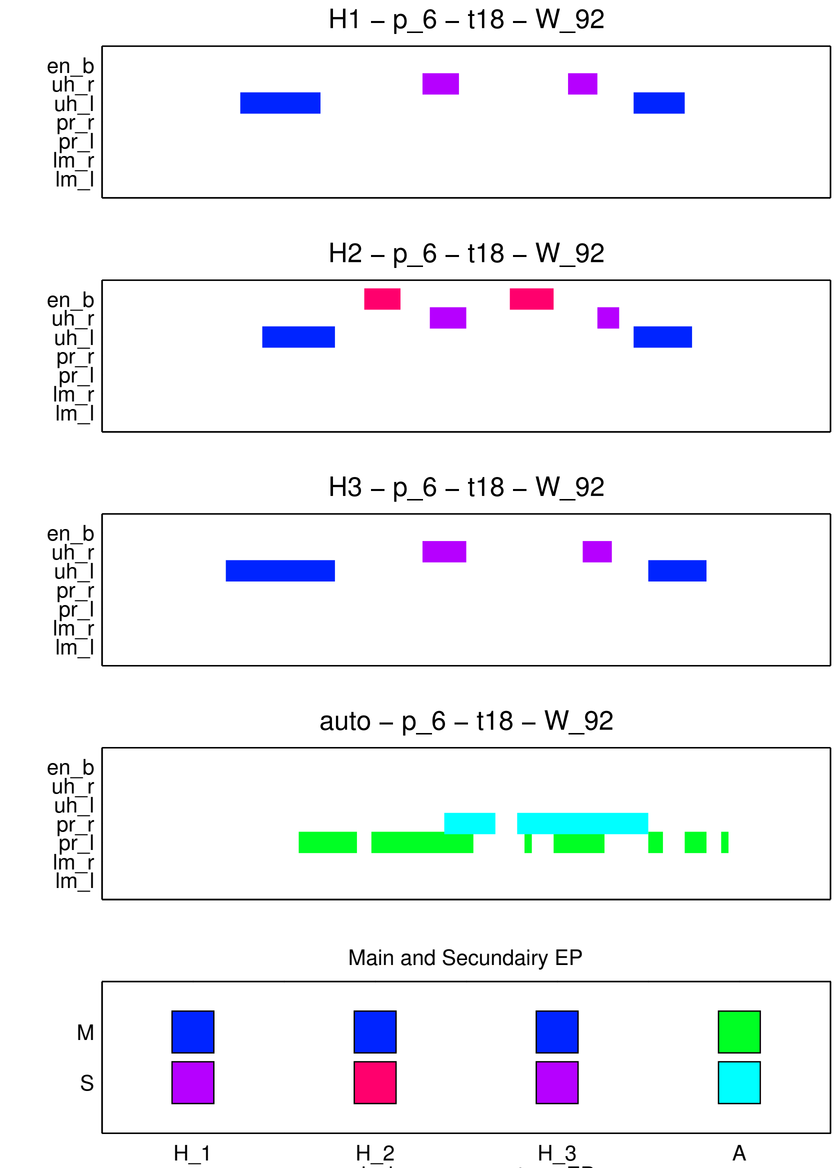

pairwise agreement per EP

	L–l	L–r	P–l	P–r	U–l	U–r	E–b		all
H_1–H_2	100	100	100	100	94	91	87		72
H_1–H_3	100	100	100	100	91	95	100		86
H_2–H_3	100	100	100	100	91	96	87		74
A–H_1	100	100	57	73	80	89	100		36
A–H_2	100	100	57	73	80	90	87		37
A–H_3	100	100	57	73	75	88	100		34

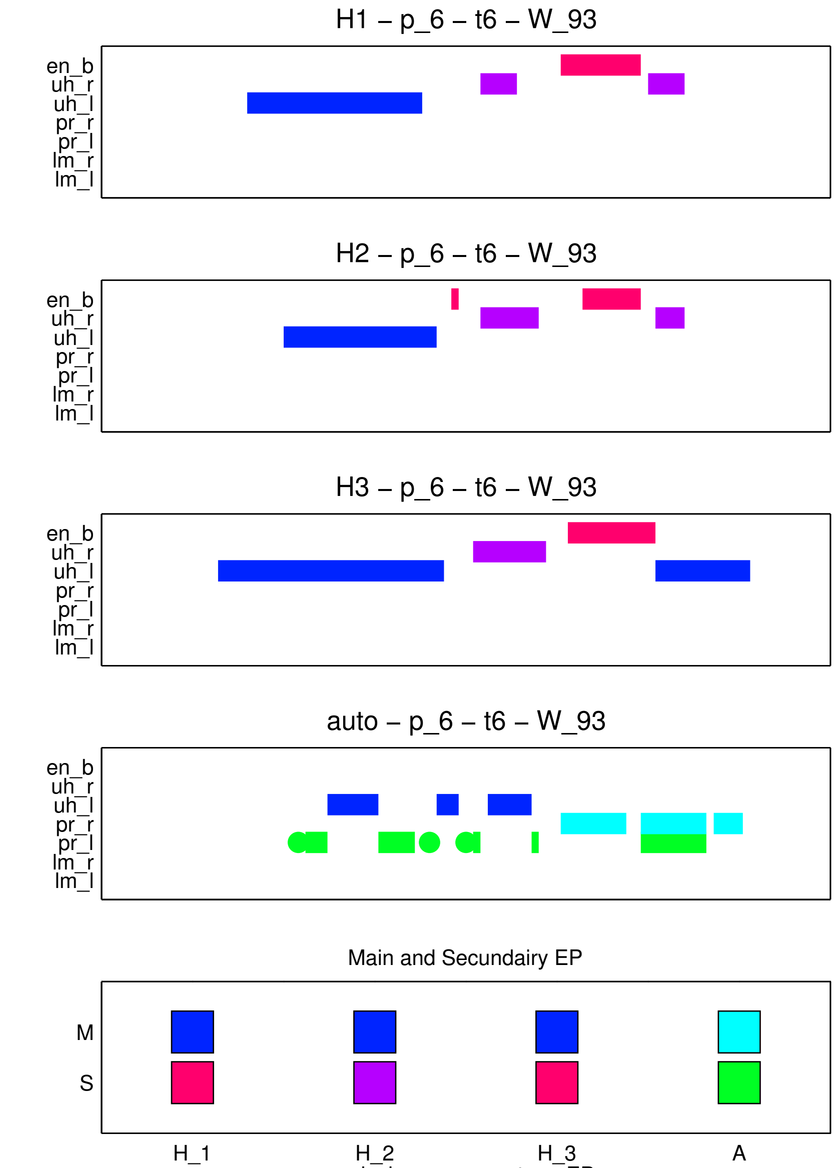

pairwise agreement per EP

	L–l	L–r	P–l	P–r	U–l	U–r	E–b		all
H_1–H_2	100	100	100	100	93	96	95		84
H_1–H_3	100	100	100	100	79	89	97		72
H_2–H_3	100	100	100	100	76	93	94		69
A–H_1	100	100	73	75	72	88	88		39
A–H_2	100	100	73	75	77	86	89		45
A–H_3	100	100	73	75	55	89	87		35

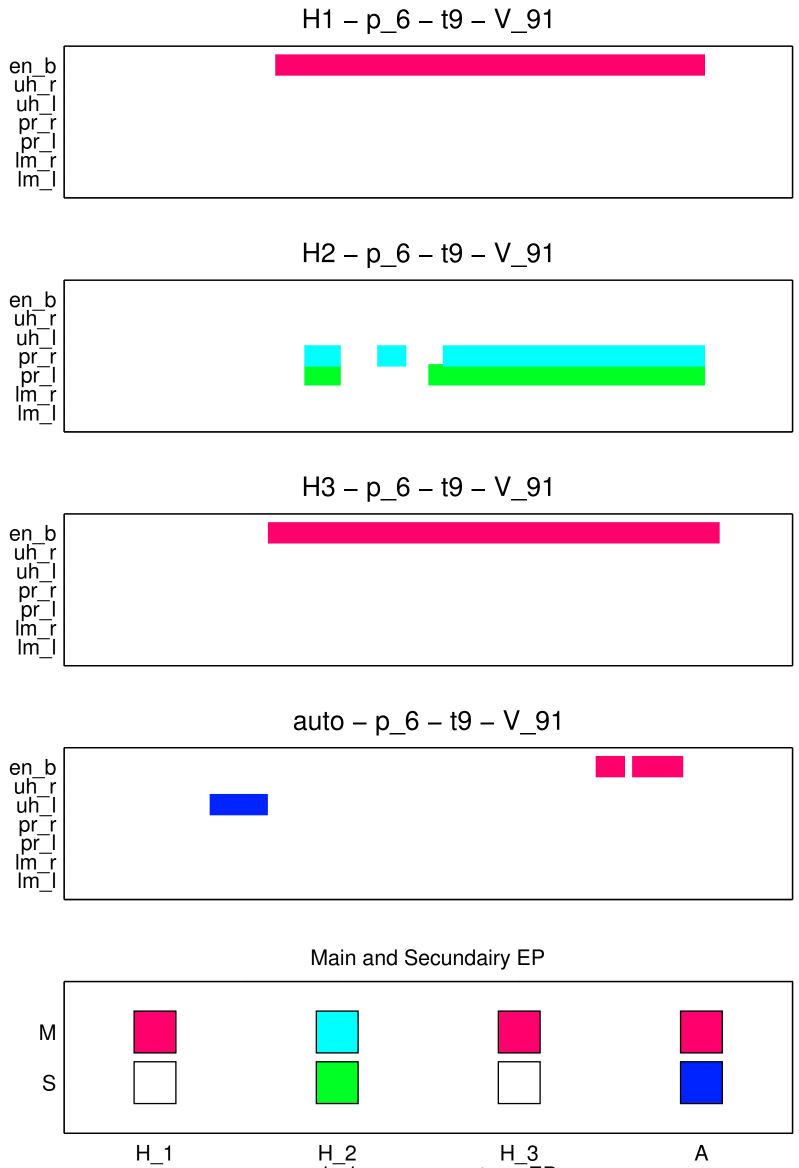

pairwise agreement per EP

	L–l	L–r	P–l	P–r	U–l	U–r	E–b		all
H_1–H_2	100	100	55	52	100	100	40		40
H_1–H_3	100	100	100	100	100	100	97		97
H_2–H_3	100	100	55	52	100	100	37		37
A–H_1	100	100	100	100	91	100	53		44
A–H_2	100	100	55	52	91	100	87		41
A–H_3	100	100	100	100	91	100	50		42

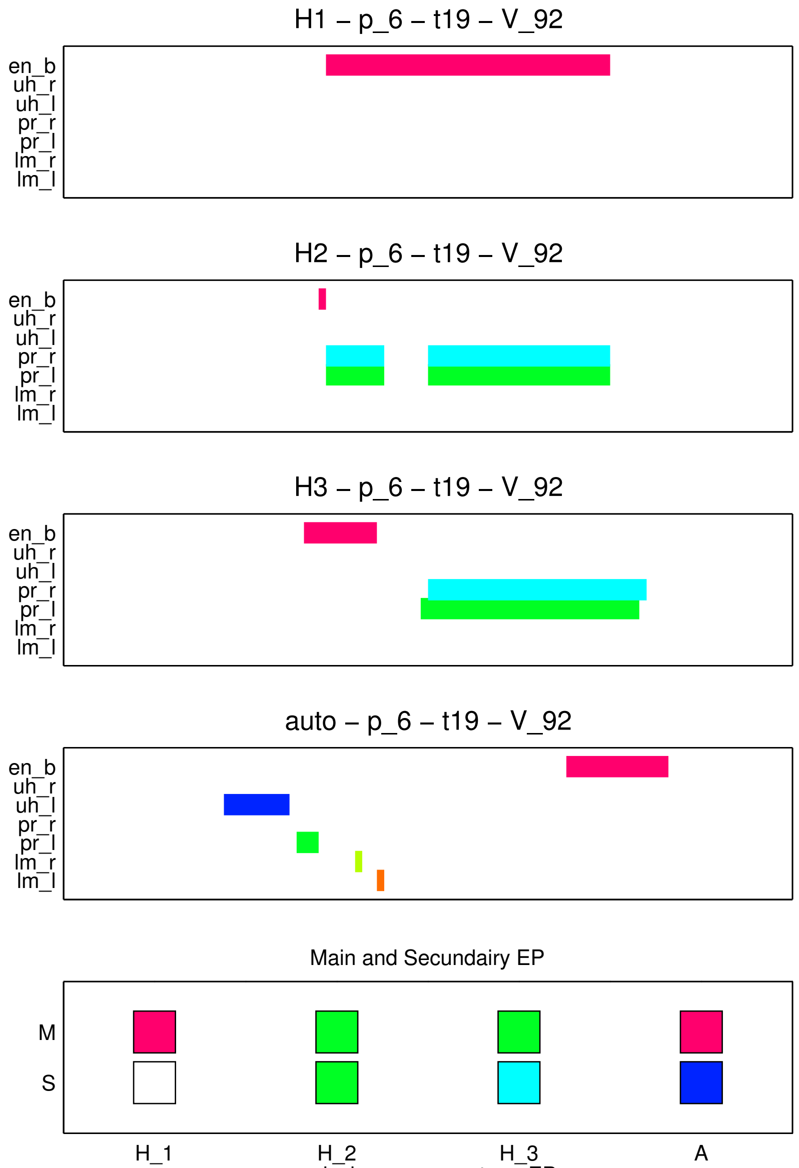

pairwise agreement per EP

	L–l	L–r	P–l	P–r	U–l	U–r	E–b		all
H_1–H_2	100	100	65	65	100	100	60		59
H_1–H_3	100	100	69	69	100	100	65		60
H_2–H_3	100	100	86	86	100	100	91		83
A–H_1	98	98	96	100	90	100	59		45
A–H_2	98	98	61	65	90	100	83		43
A–H_3	98	98	65	69	90	100	74		42

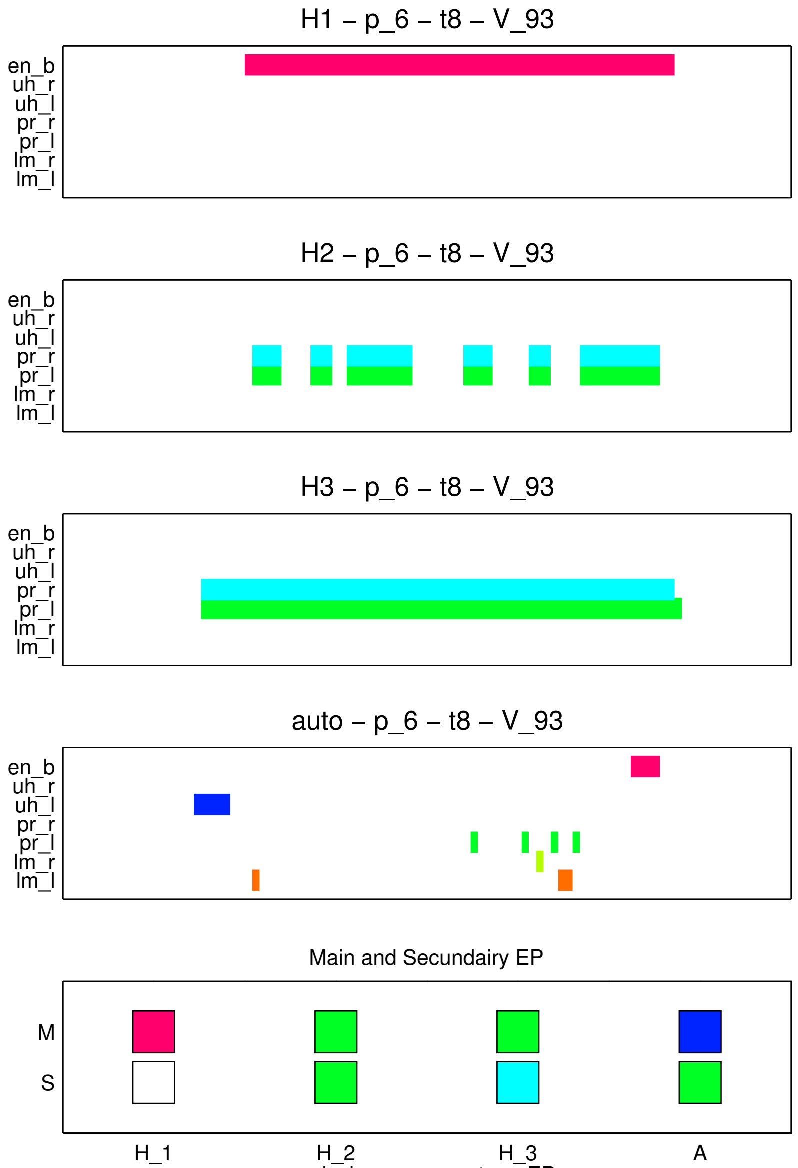

pairwise agreement per EP

	L–l	L–r	P–l	P–r	U–l	U–r	E–b		all
H_1–H_2	100	100	60	60	100	100	40		40
H_1–H_3	100	100	33	34	100	100	40		33
H_2–H_3	100	100	73	74	100	100	100		73
A–H_1	95	98	92	100	94	100	45		39
A–H_2	95	98	62	60	94	100	95		50
A–H_3	95	98	41	34	94	100	95		32

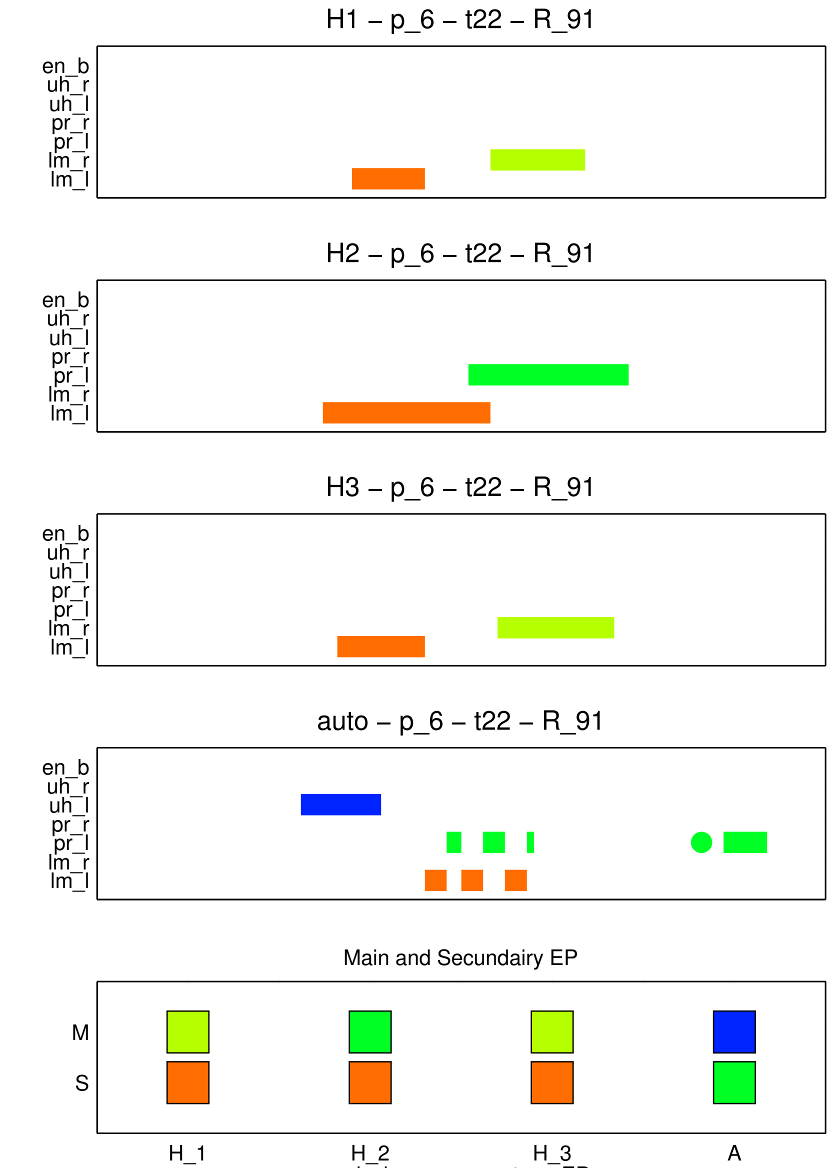

pairwise agreement per EP

	L–l	L–r	P–l	P–r	U–l	U–r	E–b		all
H_1–H_2	87	86	77	100	100	100	100		68
H_1–H_3	98	95	100	100	100	100	100		93
H_2–H_3	89	83	77	100	100	100	100		70
A–H_1	79	86	83	100	88	100	100		53
A–H_2	80	100	72	100	88	100	100		52
A–H_3	77	83	83	100	88	100	100		49

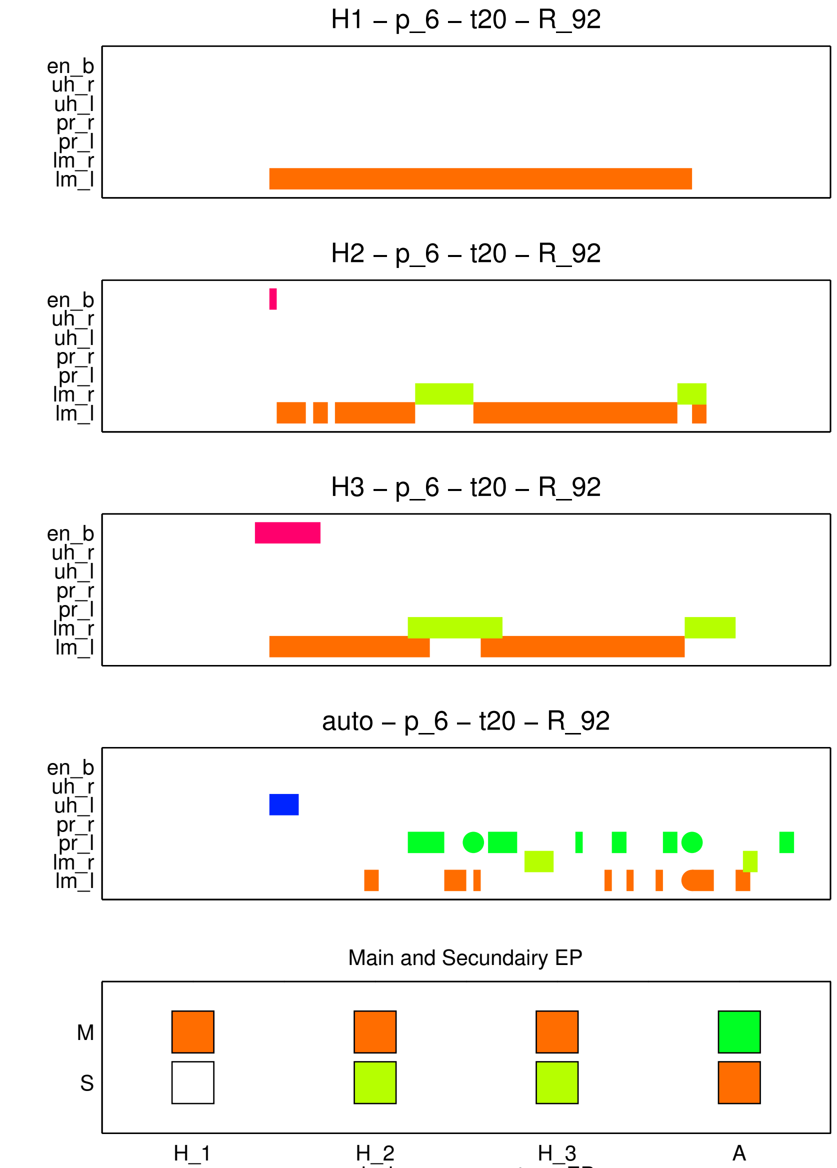

pairwise agreement per EP

	L–l	L–r	P–l	P–r	U–l	U–r	E–b		all
H_1–H_2	89	86	100	100	100	100	98		84
H_1–H_3	93	78	100	100	100	100	90		68
H_2–H_3	92	90	100	100	100	100	92		74
A–H_1	51	92	76	100	95	100	100		41
A–H_2	54	78	76	100	95	100	98		38
A–H_3	46	70	76	100	95	100	90		33

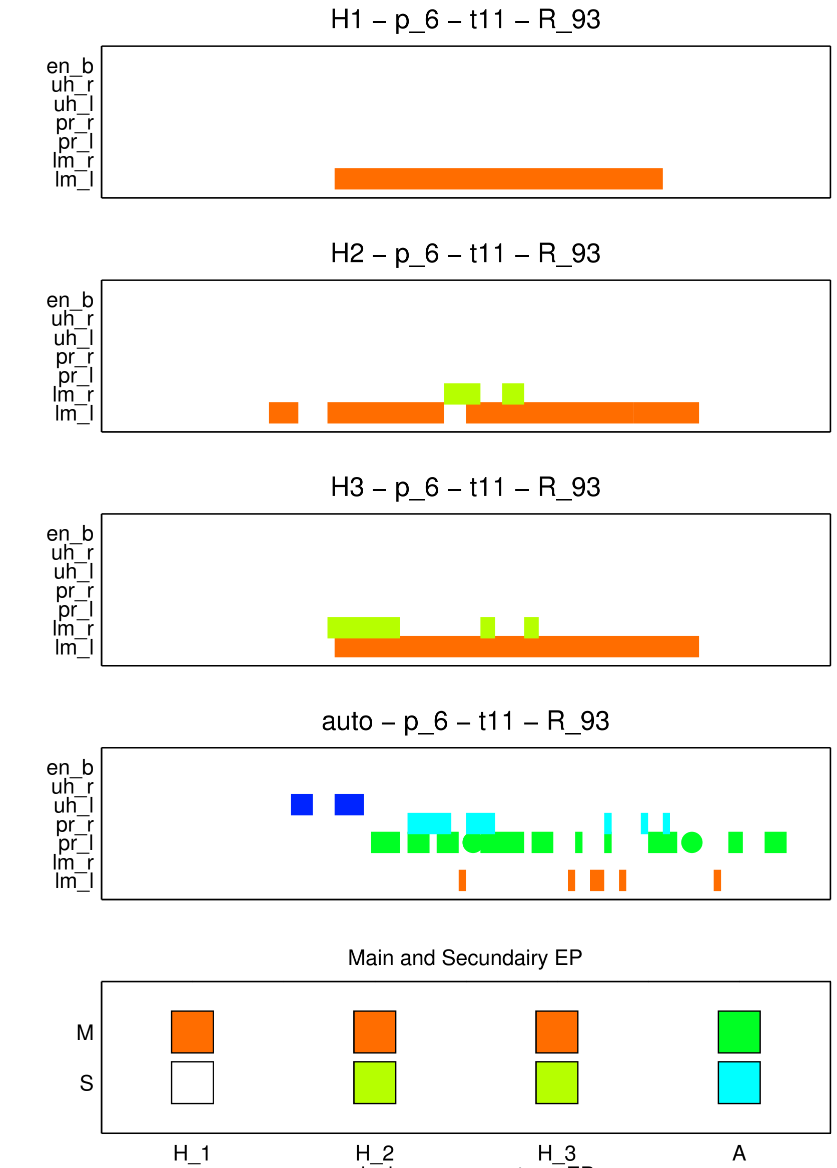

pairwise agreement per EP

	L–l	L–r	P–l	P–r	U–l	U–r	E–b		all
H_1–H_2	87	90	100	100	100	100	100		79
H_1–H_3	95	83	100	100	100	100	100		78
H_2–H_3	92	77	100	100	100	100	100		72
A–H_1	61	100	58	82	91	100	100		43
A–H_2	50	90	58	82	91	100	100		37
A–H_3	56	83	58	82	91	100	100		40
