# Supplementary material for: Haptic Exploratory Behavior During Object Discrimination: A Novel Automatic Annotation Method
Source: PLoS One. 2015 Feb 6;10(2):e0117017. doi: 10.1371/journal.pone.0117017 (PMC4319767; doi:10.1371/journal.pone.0117017)
Supplement: S4 Annotation Output — (PDF) [file pone.0117017.s004.pdf]

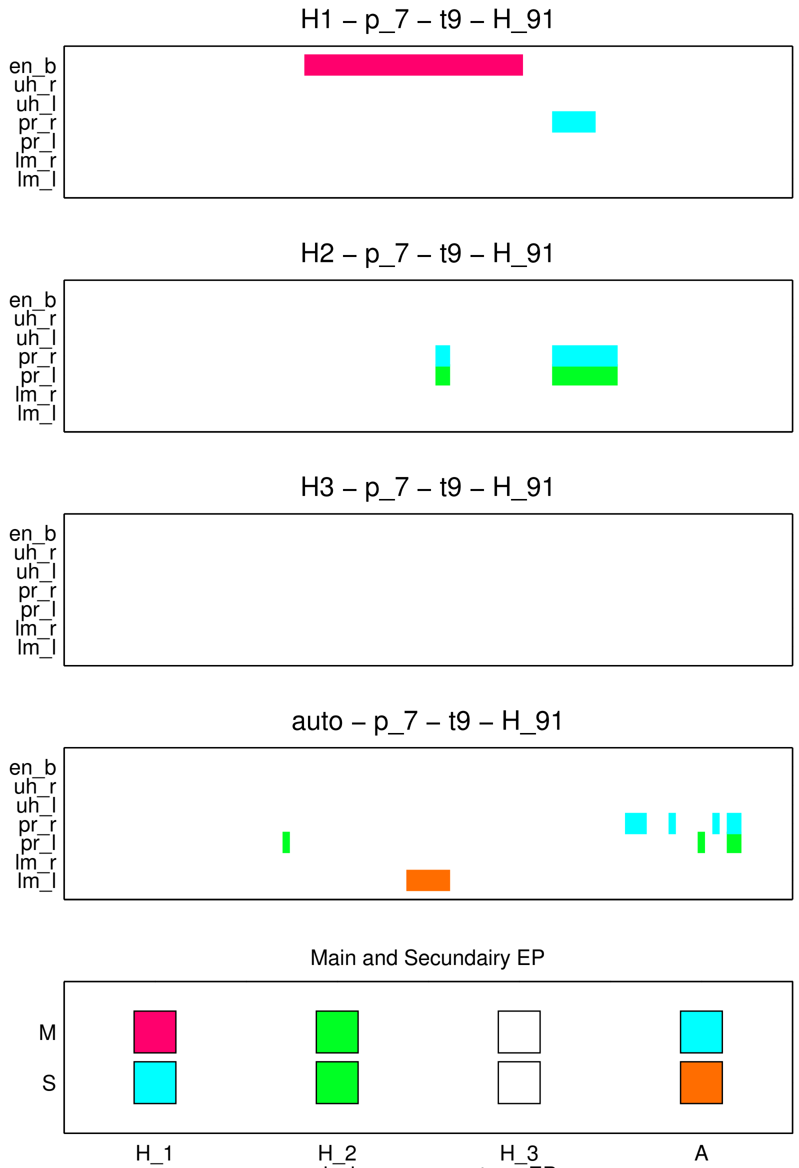

pairwise agreement per EP

	L–l	L–r	P–l	P–r	U–l	U–r	E–b		all
H_1–H_2	100	100	87	94	100	100	69		59
H_1–H_3	100	100	100	93	100	100	69		62
H_2–H_3	100	100	87	87	100	100	100		87
A–H_1	93	100	93	82	100	100	69		47
A–H_2	93	100	80	76	100	100	100		68
A–H_3	93	100	93	89	100	100	100		78

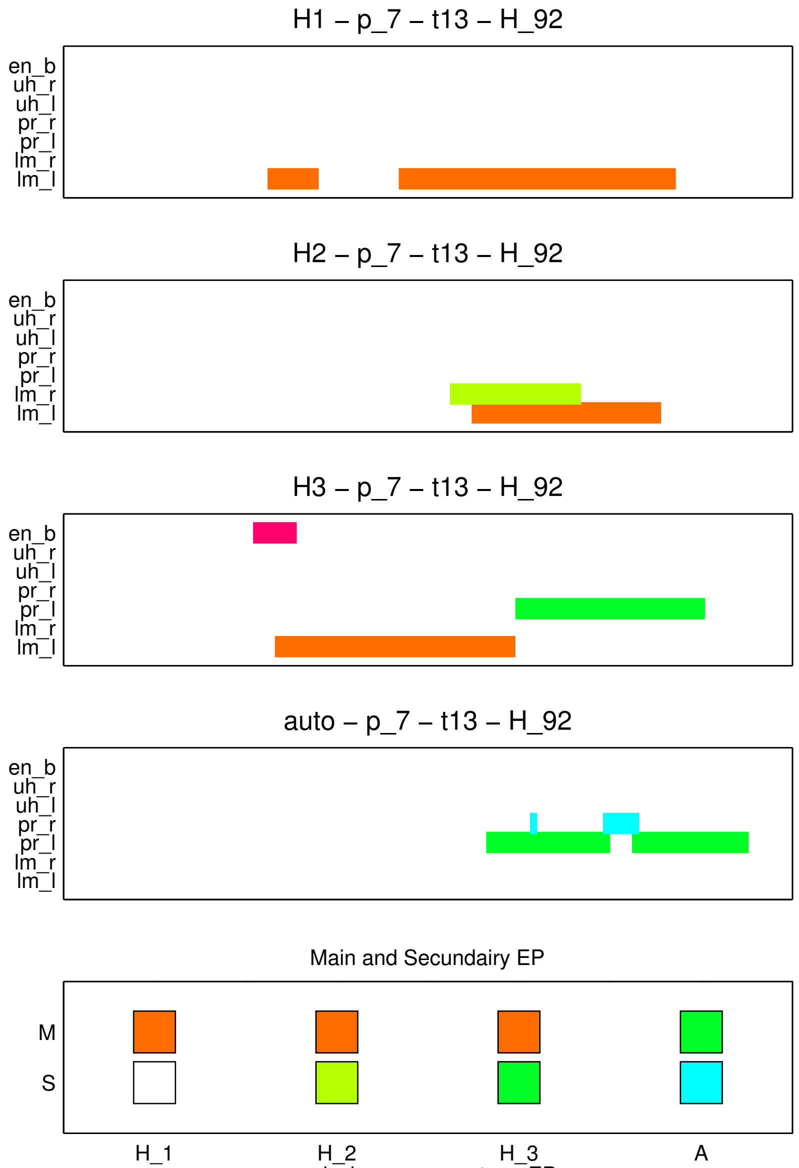

pairwise agreement per EP

	L–l	L–r	P–l	P–r	U–l	U–r	E–b		all
H_1–H_2	80	81	100	100	100	100	100		64
H_1–H_3	67	100	73	100	100	100	93		56
H_2–H_3	53	81	73	100	100	100	93		37
A–H_1	53	100	65	92	100	100	100		43
A–H_2	73	81	65	92	100	100	100		58
A–H_3	66	100	88	92	100	100	93		49

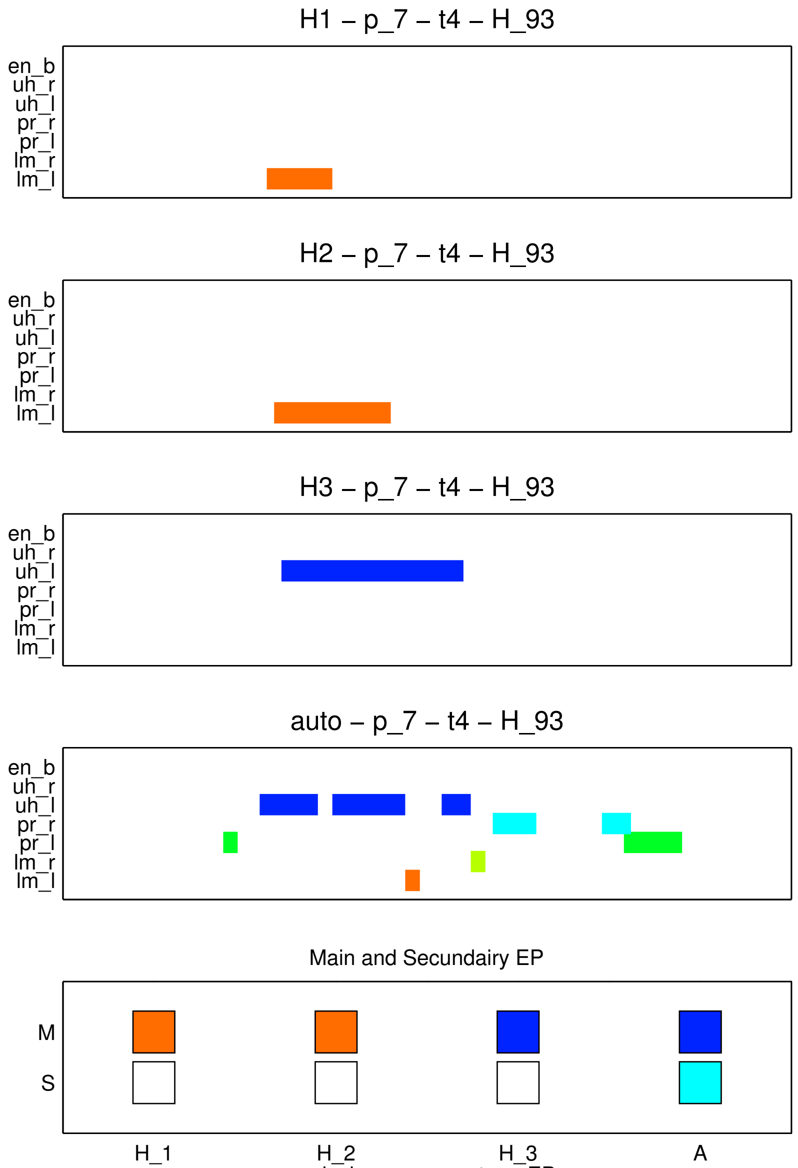

pairwise agreement per EP

	L–l	L–r	P–l	P–r	U–l	U–r	E–b		all
H_1–H_2	91	100	100	100	100	100	100		91
H_1–H_3	90	100	100	100	74	100	100		72
H_2–H_3	83	100	100	100	74	100	100		73
A–H_1	87	97	88	88	75	100	100		48
A–H_2	80	97	88	88	75	100	100		48
A–H_3	97	97	88	88	91	100	100		66

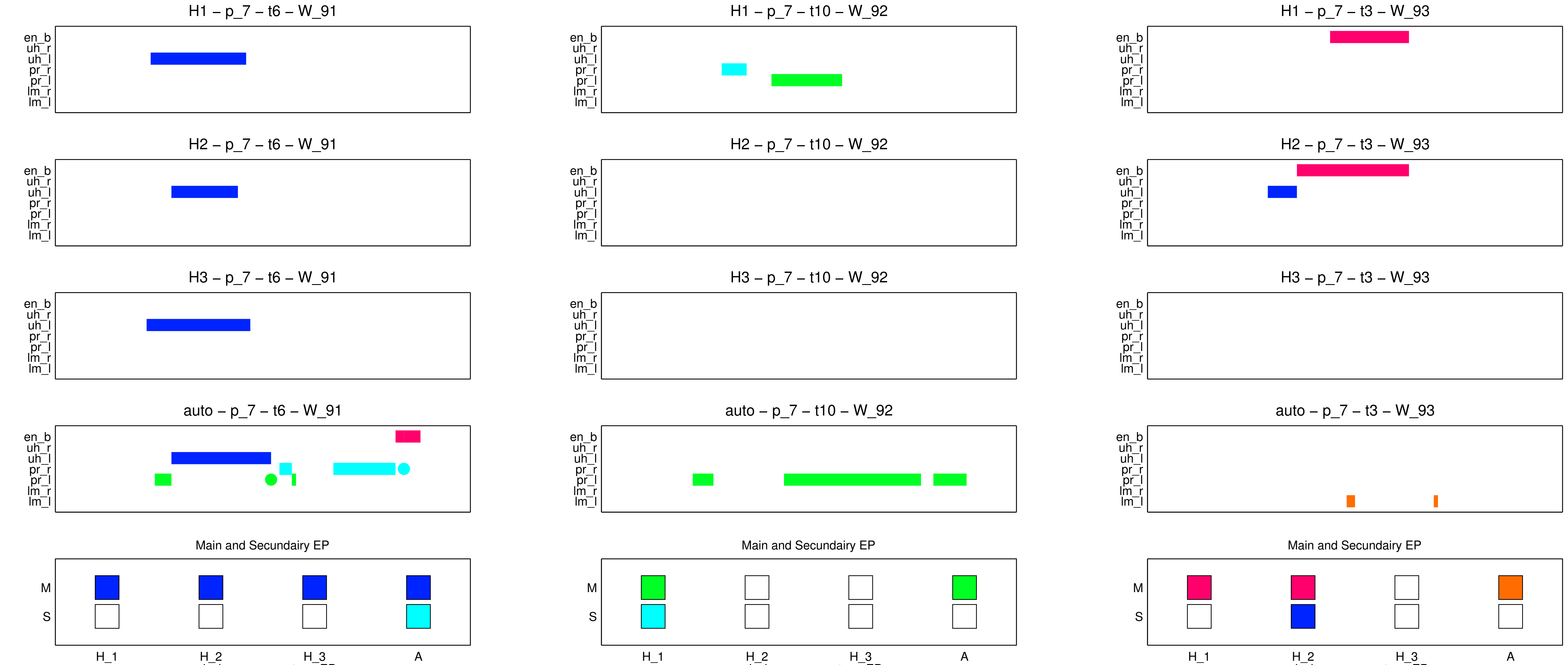

pairwise agreement per EP

	L–l	L–r	P–l	P–r	U–l	U–r	E–b		all
H_1–H_2	100	100	100	100	93	100	100		93
H_1–H_3	100	100	100	100	98	100	100		98
H_2–H_3	100	100	100	100	91	100	100		91
A–H_1	100	100	92	79	89	100	93		61
A–H_2	100	100	92	79	92	100	93		60
A–H_3	100	100	92	79	89	100	93		61

pairwise agreement per EP

	L–l	L–r	P–l	P–r	U–l	U–r	E–b		all
H_1–H_2	100	100	82	93	100	100	100		75
H_1–H_3	100	100	82	93	100	100	100		75
H_2–H_3	100	100	100	100	100	100	100		100
A–H_1	100	100	63	93	100	100	100		56
A–H_2	100	100	51	100	100	100	100		51
A–H_3	100	100	51	100	100	100	100		51

pairwise agreement per EP

	L–l	L–r	P–l	P–r	U–l	U–r	E–b		all
H_1–H_2	100	100	100	100	92	100	92		85
H_1–H_3	100	100	100	100	100	100	80		80
H_2–H_3	100	100	100	100	92	100	72		65
A–H_1	95	100	100	100	100	100	80		78
A–H_2	95	100	100	100	92	100	72		63
A–H_3	95	100	100	100	100	100	100		95

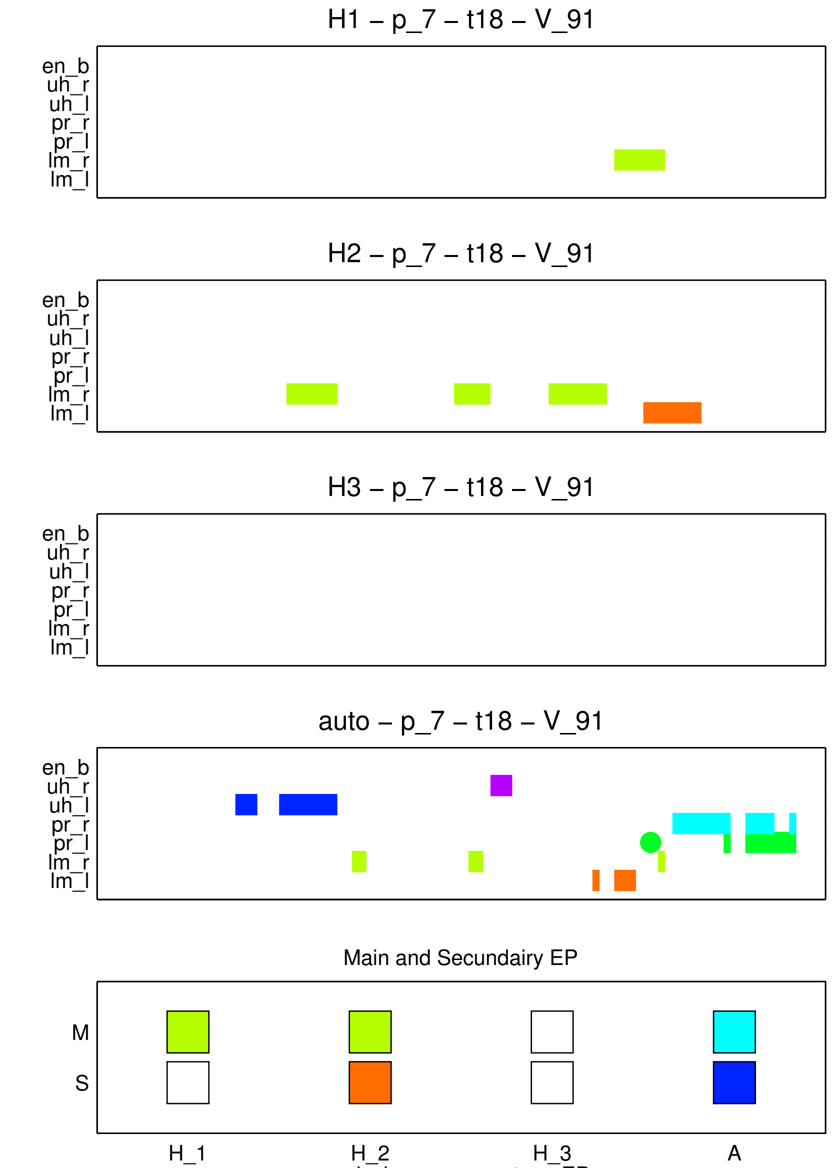

pairwise agreement per EP

	L–l	L–r	P–l	P–r	U–l	U–r	E–b		all
H_1–H_2	91	69	100	100	100	100	100		64
H_1–H_3	100	92	100	100	100	100	100		92
H_2–H_3	91	77	100	100	100	100	100		68
A–H_1	94	88	89	84	87	96	100		52
A–H_2	85	75	89	84	87	96	100		44
A–H_3	94	92	89	84	87	96	100		51

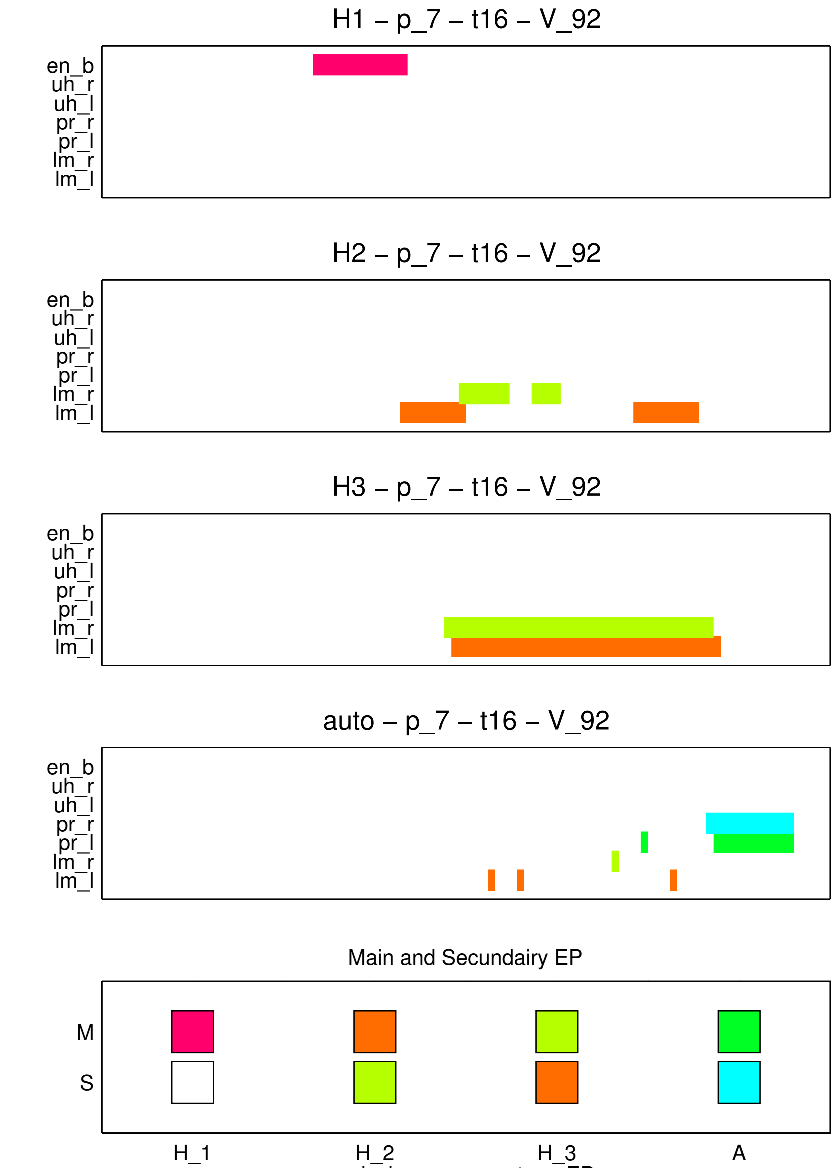

pairwise agreement per EP

	L–l	L–r	P–l	P–r	U–l	U–r	E–b		all
H_1–H_2	80	87	100	100	100	100	86		57
H_1–H_3	62	62	100	100	100	100	86		47
H_2–H_3	68	75	100	100	100	100	100		57
A–H_1	94	98	86	87	100	100	86		63
A–H_2	78	85	86	87	100	100	100		54
A–H_3	68	64	86	87	100	100	100		51

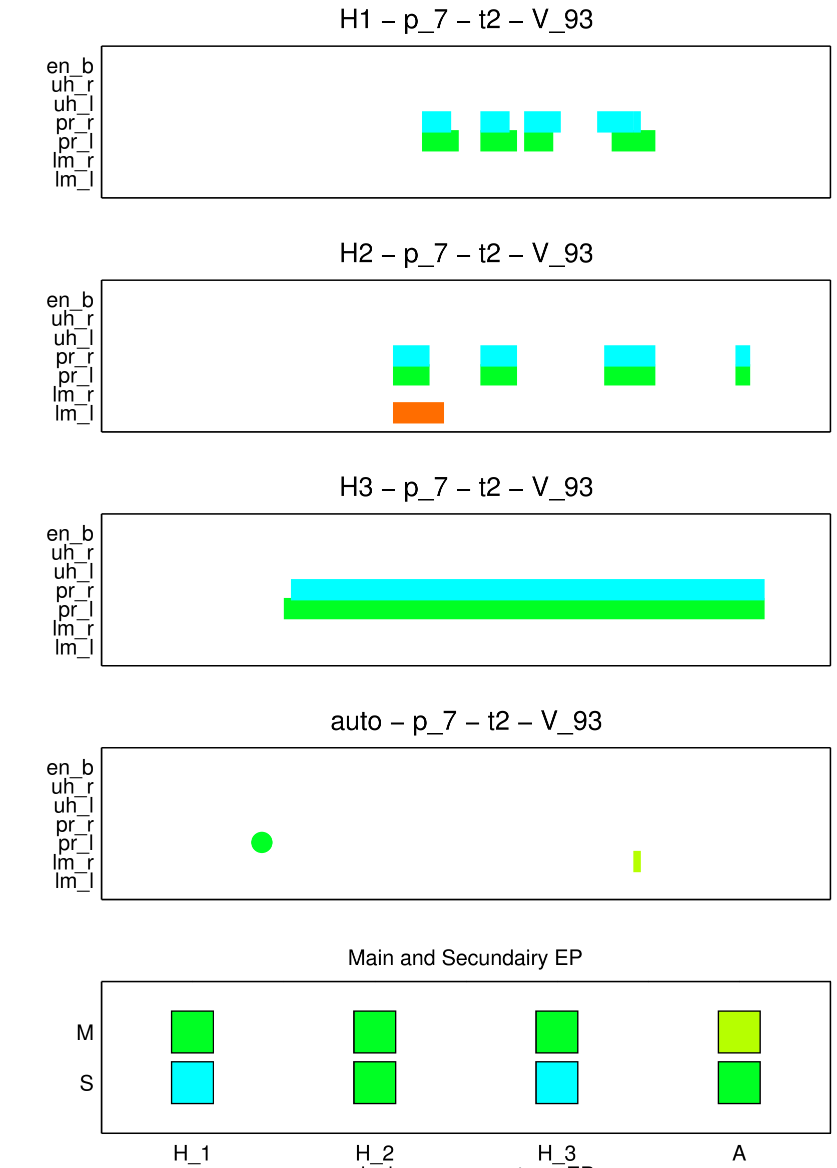

pairwise agreement per EP

	L–l	L–r	P–l	P–r	U–l	U–r	E–b		all
H_1–H_2	92	100	83	80	100	100	100		76
H_1–H_3	100	100	57	57	100	100	100		53
H_2–H_3	92	100	56	57	100	100	100		50
A–H_1	100	98	75	77	100	100	100		72
A–H_2	92	98	76	77	100	100	100		74
A–H_3	100	98	32	34	100	100	100		32

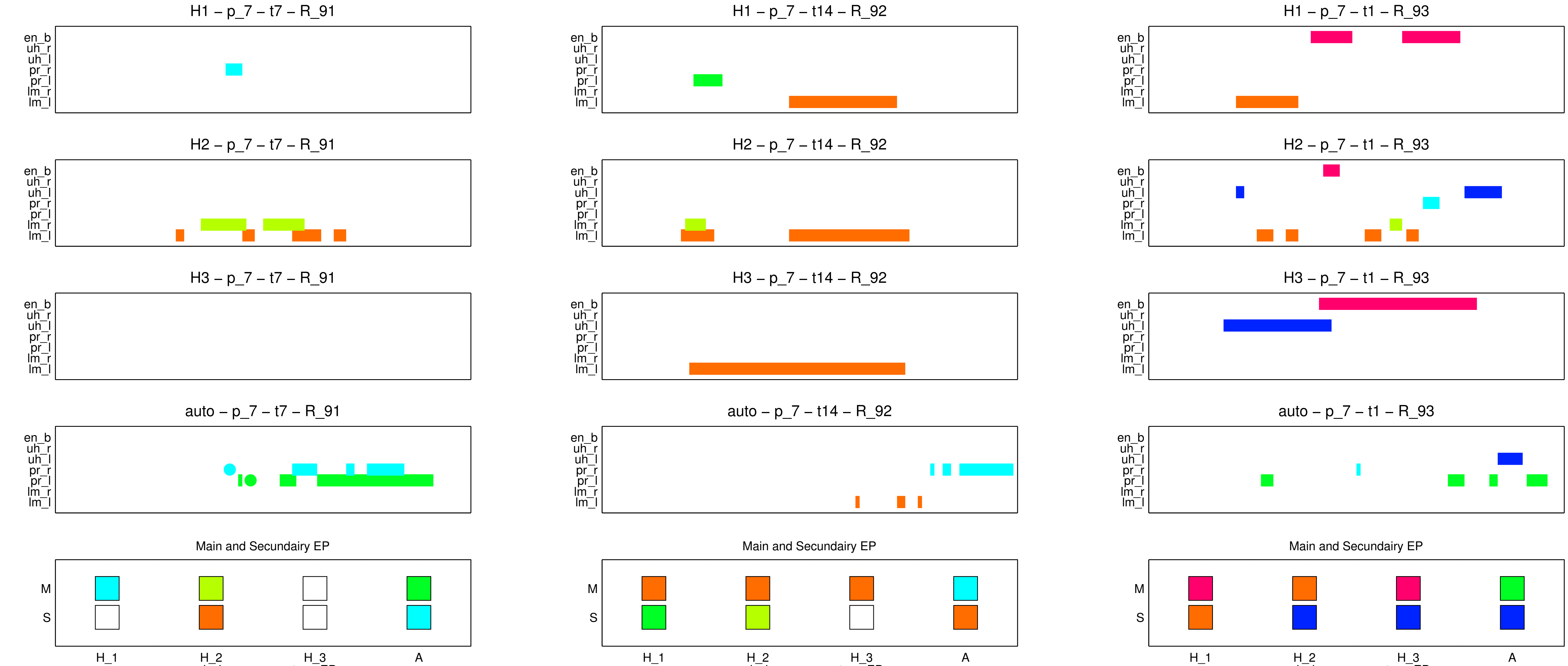

pairwise agreement per EP

	L–l	L–r	P–l	P–r	U–l	U–r	E–b		all
H_1–H_2	81	77	100	95	100	100	100		64
H_1–H_3	100	100	100	95	100	100	100		95
H_2–H_3	81	77	100	100	100	100	100		64
A–H_1	100	100	63	76	100	100	100		57
A–H_2	81	77	63	79	100	100	100		41
A–H_3	100	100	63	79	100	100	100		58

pairwise agreement per EP

	L–l	L–r	P–l	P–r	U–l	U–r	E–b		all
H_1–H_2	88	94	92	100	100	100	100		86
H_1–H_3	74	100	92	100	100	100	100		74
H_2–H_3	80	94	100	100	100	100	100		75
A–H_1	72	100	92	81	100	100	100		45
A–H_2	64	94	100	81	100	100	100		45
A–H_3	50	100	100	81	100	100	100		31

pairwise agreement per EP

	L–l	L–r	P–l	P–r	U–l	U–r	E–b		all
H_1–H_2	84	96	100	95	87	100	79		54
H_1–H_3	84	100	100	100	73	100	83		58
H_2–H_3	82	96	100	95	66	100	66		37
A–H_1	84	100	82	98	93	100	74		40
A–H_2	82	96	82	93	84	100	95		39
A–H_3	100	100	82	98	66	100	61		23
