# Supplementary material for: Haptic Exploratory Behavior During Object Discrimination: A Novel Automatic Annotation Method
Source: PLoS One. 2015 Feb 6;10(2):e0117017. doi: 10.1371/journal.pone.0117017 (PMC4319767; doi:10.1371/journal.pone.0117017)
Supplement: S5 Annotation Output — (PDF) [file pone.0117017.s005.pdf]

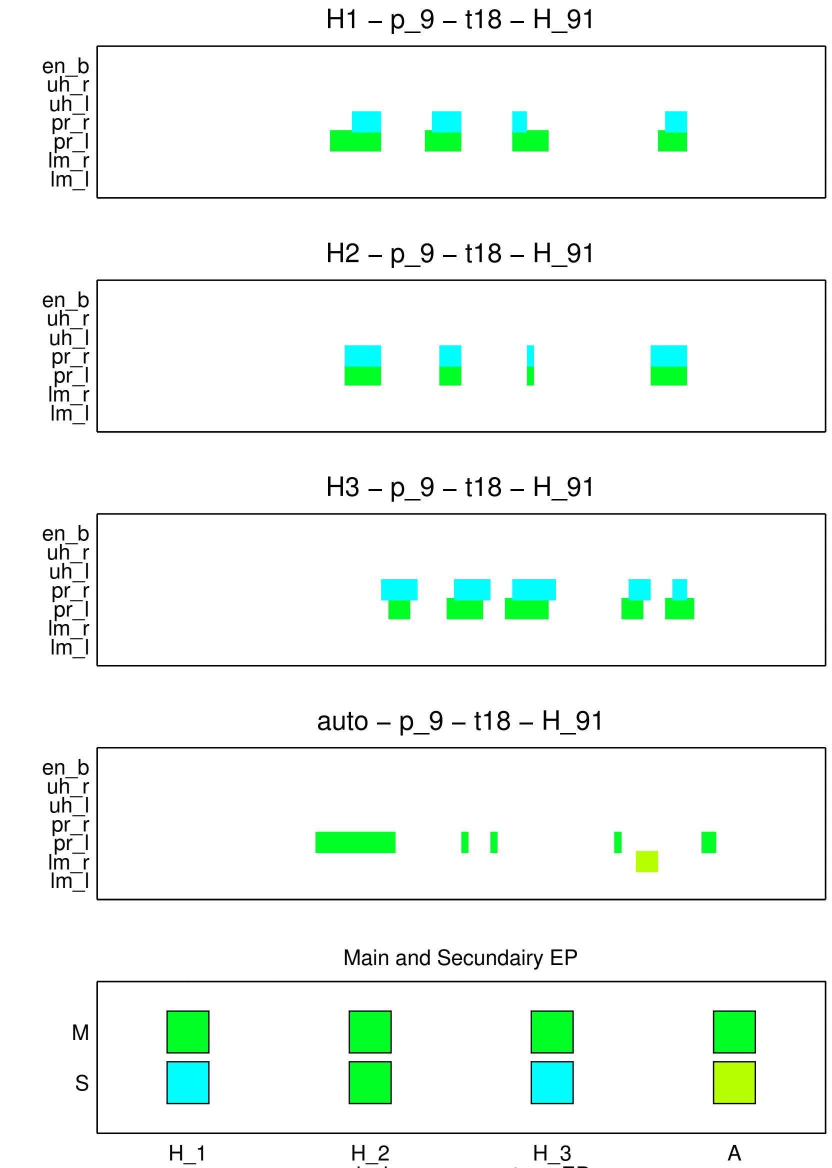

pairwise agreement per EP

	L–l	L–r	P–l	P–r	U–l	U–r	E–b		all
H_1–H_2	100	100	91	93	100	100	100		88
H_1–H_3	100	100	75	75	100	100	100		66
H_2–H_3	100	100	74	74	100	100	100		69
A–H_1	100	96	72	83	100	100	100		63
A–H_2	100	96	75	82	100	100	100		66
A–H_3	100	96	63	74	100	100	100		55

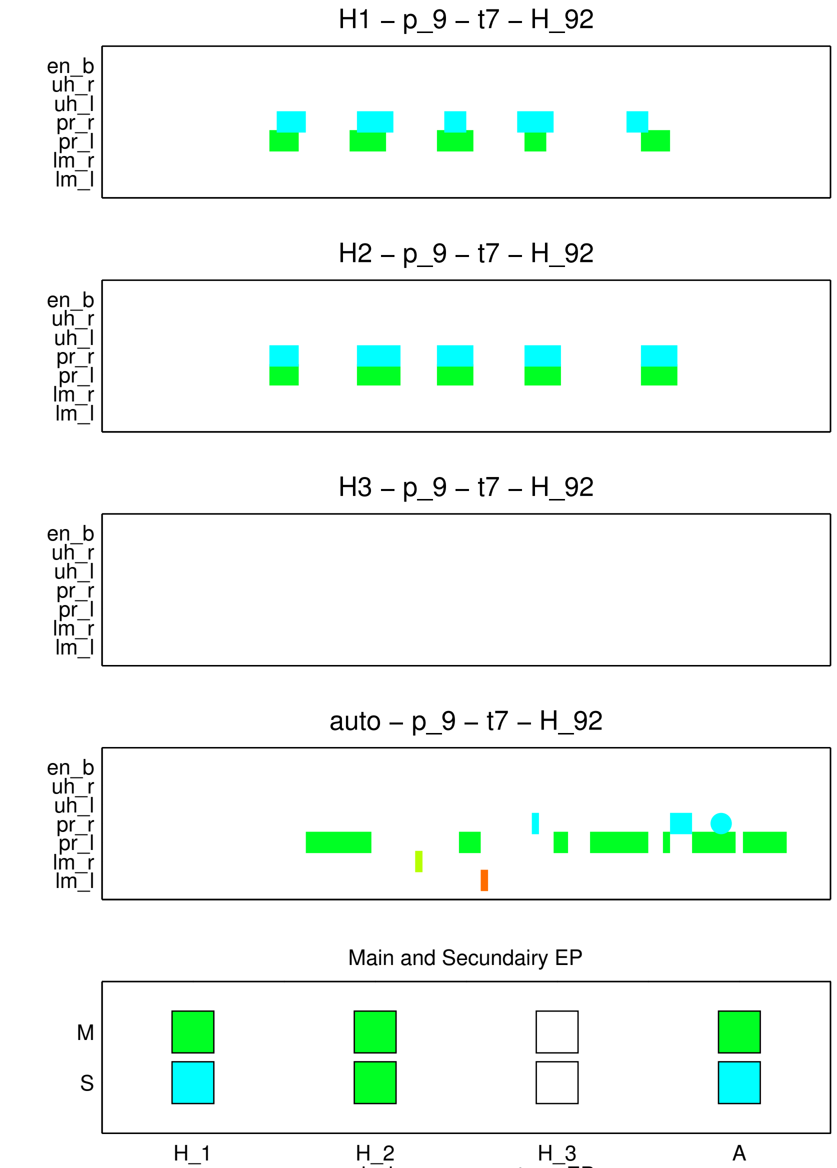

pairwise agreement per EP

	L–l	L–r	P–l	P–r	U–l	U–r	E–b		all
H_1–H_2	100	100	94	87	100	100	100		84
H_1–H_3	100	100	74	75	100	100	100		68
H_2–H_3	100	100	70	70	100	100	100		70
A–H_1	98	98	54	72	100	100	100		39
A–H_2	98	98	52	71	100	100	100		37
A–H_3	98	98	58	93	100	100	100		51

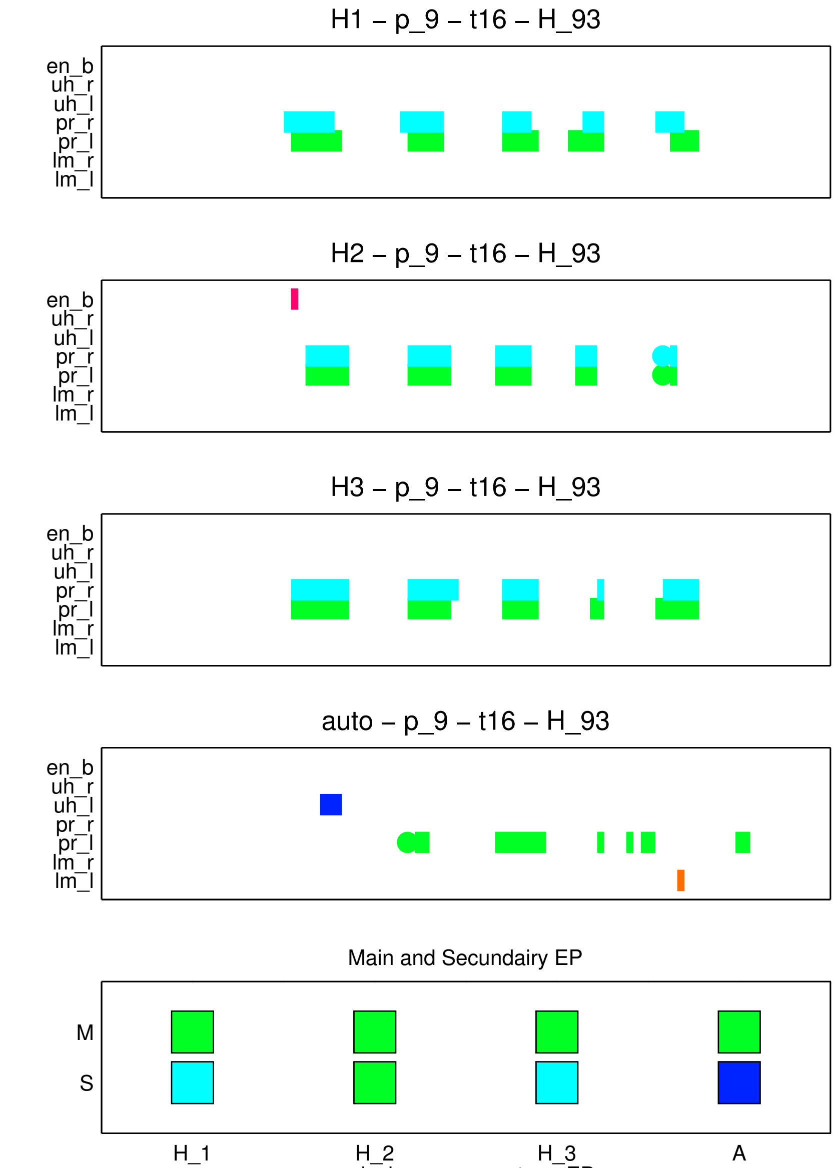

pairwise agreement per EP

	L–l	L–r	P–l	P–r	U–l	U–r	E–b		all
H_1–H_2	100	100	88	88	100	100	98		83
H_1–H_3	100	100	93	88	100	100	100		85
H_2–H_3	100	100	89	88	100	100	98		87
A–H_1	98	100	71	71	96	100	100		57
A–H_2	98	100	73	73	96	100	98		59
A–H_3	98	100	72	69	96	100	100		59

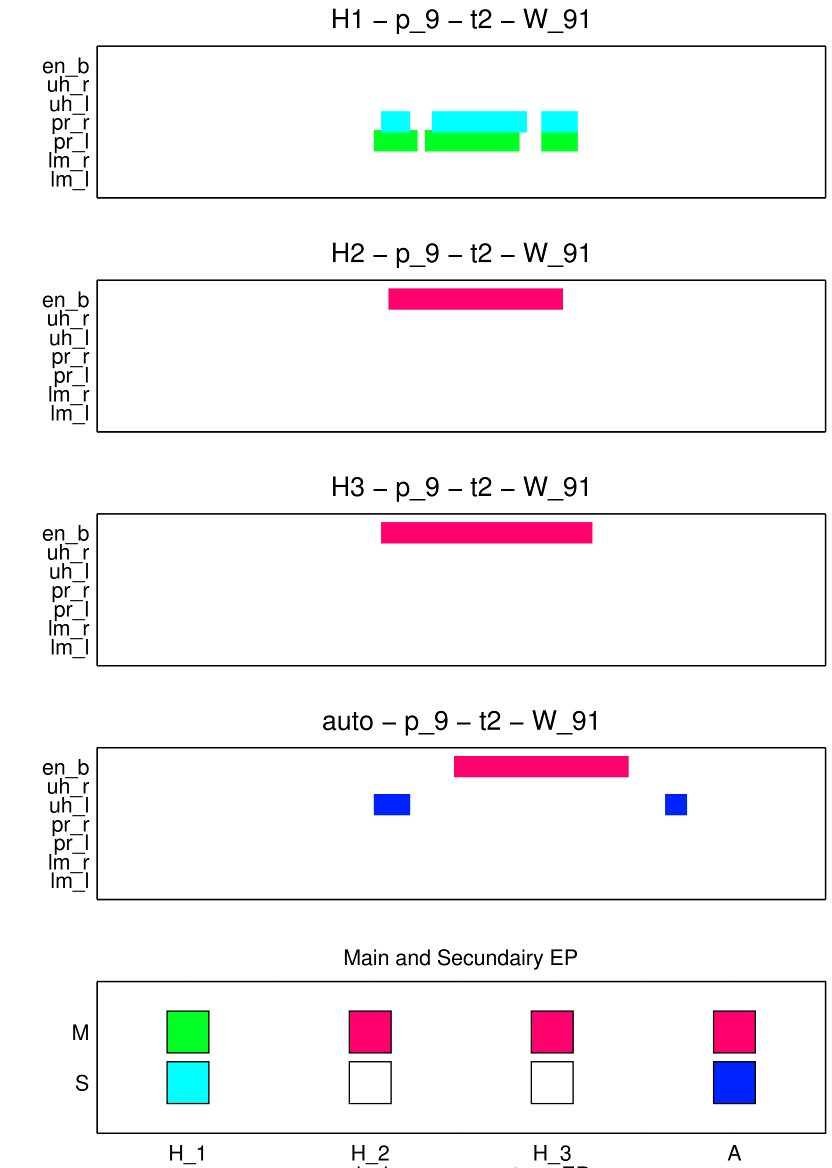

pairwise agreement per EP

	L–l	L–r	P–l	P–r	U–l	U–r	E–b		all
H_1–H_2	100	100	73	75	100	100	75		71
H_1–H_3	100	100	73	75	100	100	70		69
H_2–H_3	100	100	100	100	100	100	95		95
A–H_1	100	100	73	75	90	100	75		60
A–H_2	100	100	100	100	90	100	82		76
A–H_3	100	100	100	100	90	100	85		80

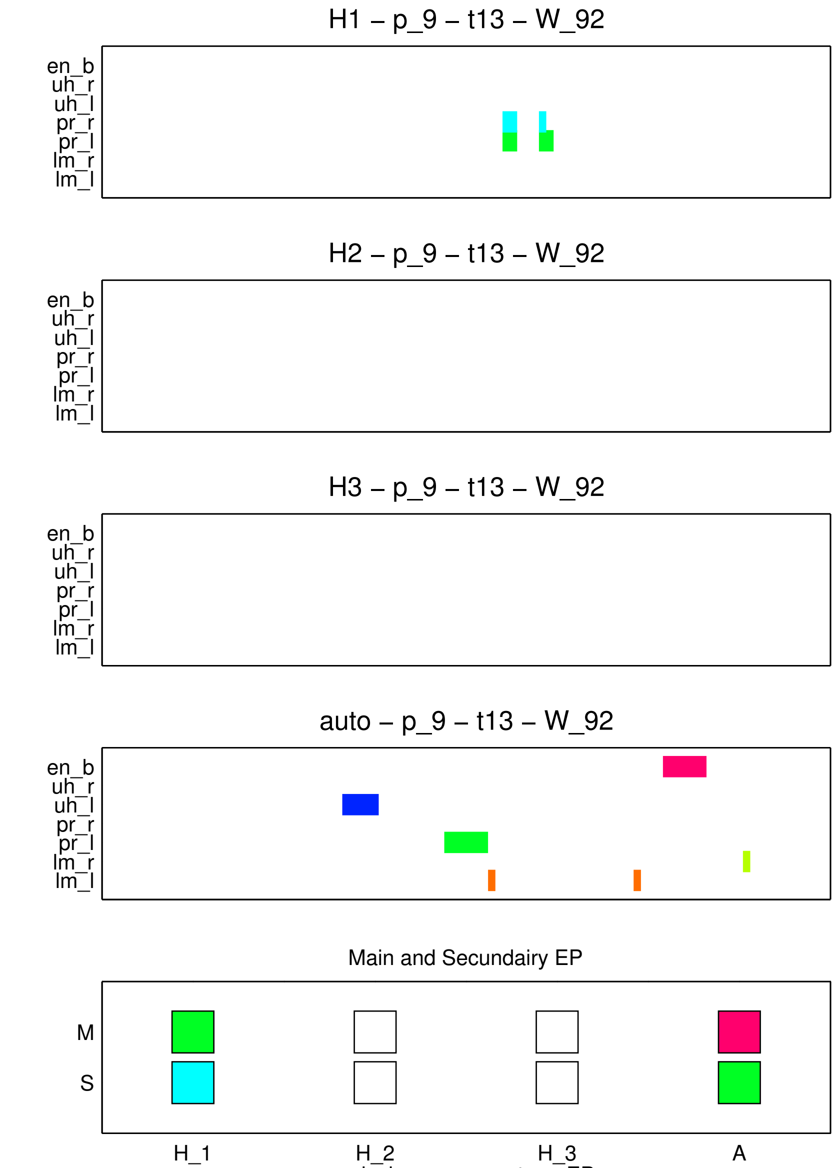

pairwise agreement per EP

	L–l	L–r	P–l	P–r	U–l	U–r	E–b		all
H_1–H_2	100	100	94	95	100	100	100		94
H_1–H_3	100	100	94	95	100	100	100		94
H_2–H_3	100	100	100	100	100	100	100		100
A–H_1	96	98	87	95	94	100	93		69
A–H_2	96	98	93	100	94	100	93		75
A–H_3	96	98	93	100	94	100	93		75

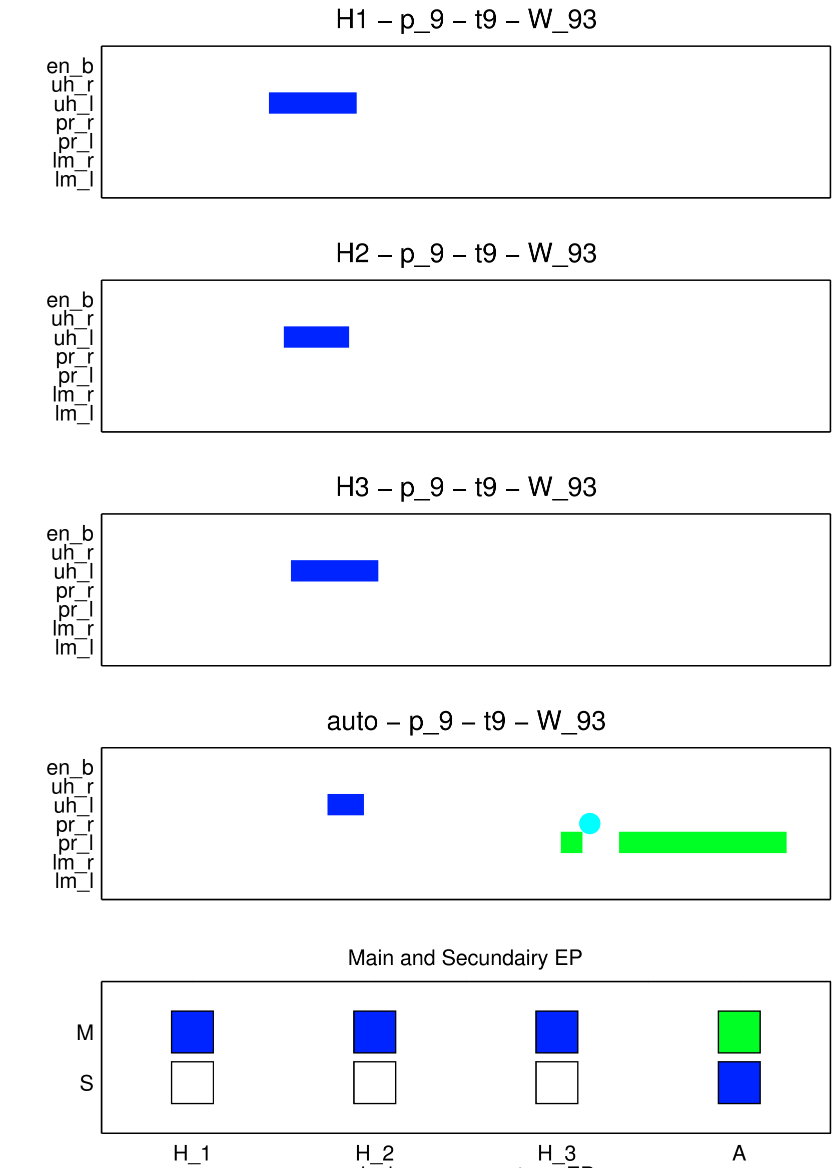

pairwise agreement per EP

	L–l	L–r	P–l	P–r	U–l	U–r	E–b		all
H_1–H_2	100	100	100	100	97	100	100		97
H_1–H_3	100	100	100	100	94	100	100		94
H_2–H_3	100	100	100	100	95	100	100		95
A–H_1	100	100	72	99	91	100	100		62
A–H_2	100	100	72	99	92	100	100		63
A–H_3	100	100	72	99	93	100	100		64

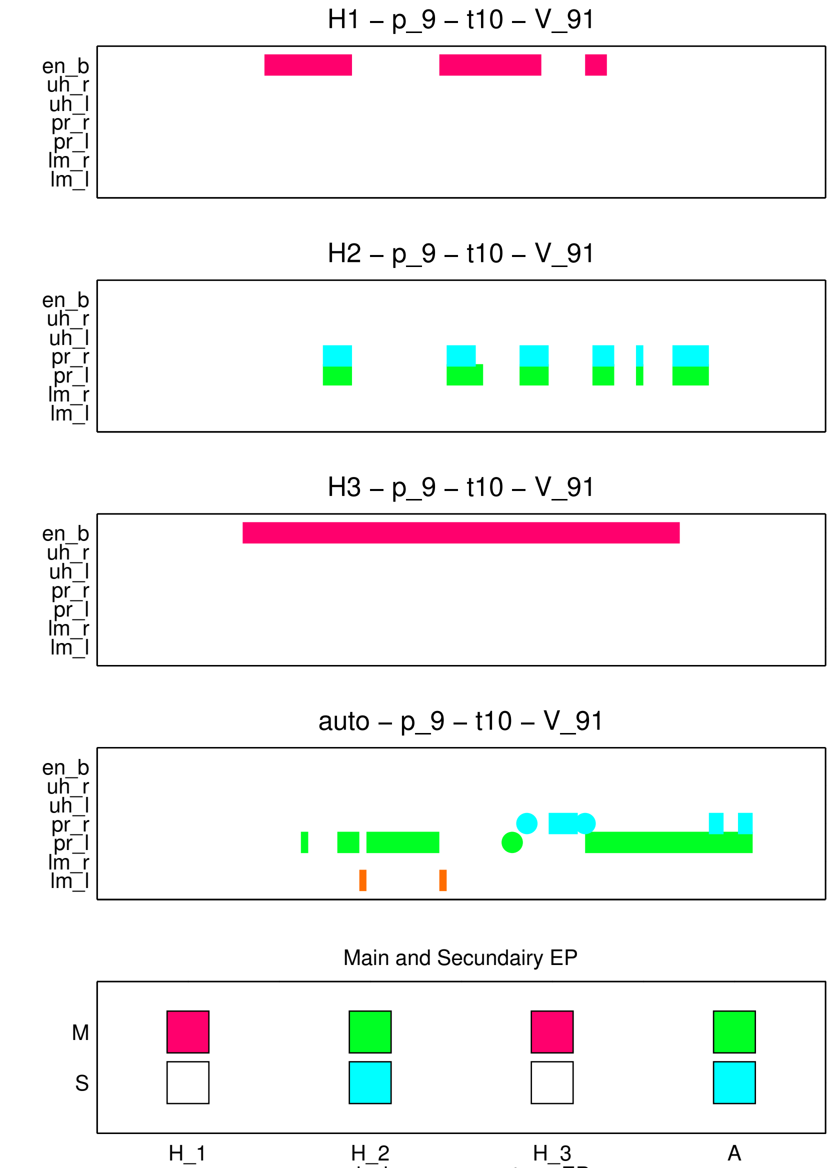

pairwise agreement per EP

	L–l	L–r	P–l	P–r	U–l	U–r	E–b		all
H_1–H_2	100	100	72	73	100	100	68		58
H_1–H_3	100	100	100	100	100	100	71		71
H_2–H_3	100	100	72	73	100	100	39		35
A–H_1	96	100	58	87	100	100	68		32
A–H_2	96	100	60	66	100	100	100		42
A–H_3	96	100	58	87	100	100	39		29

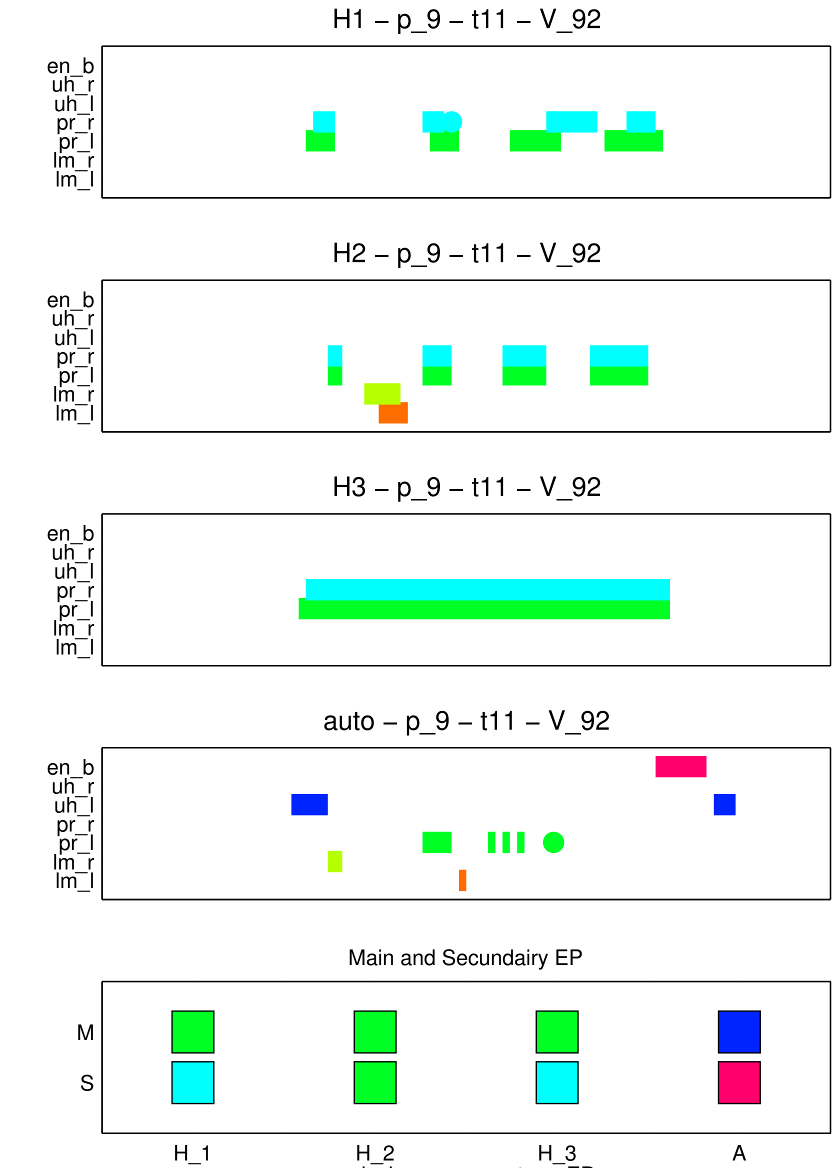

pairwise agreement per EP

	L–l	L–r	P–l	P–r	U–l	U–r	E–b		all
H_1–H_2	95	94	87	82	100	100	100		69
H_1–H_3	100	100	75	71	100	100	100		64
H_2–H_3	95	94	72	73	100	100	100		72
A–H_1	98	97	77	78	90	100	92		53
A–H_2	93	91	82	76	90	100	92		47
A–H_3	98	97	60	49	90	100	92		38

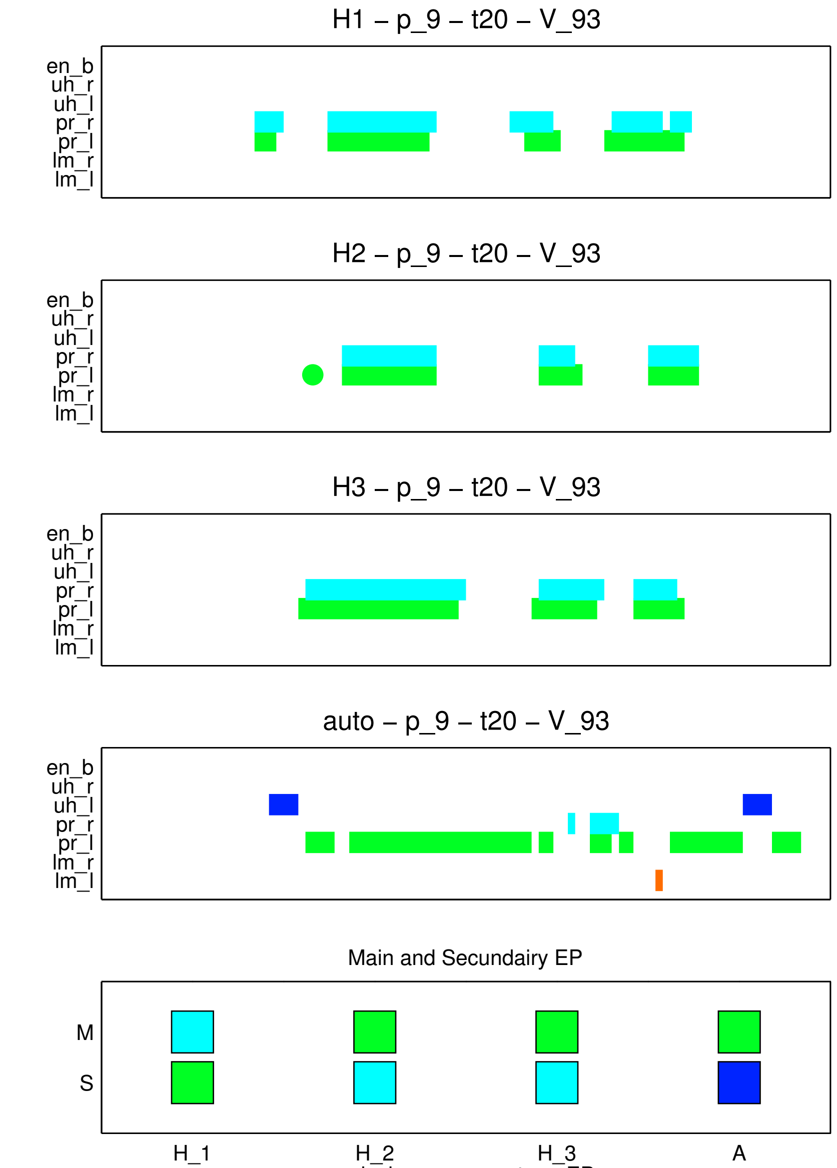

pairwise agreement per EP

	L–l	L–r	P–l	P–r	U–l	U–r	E–b		all
H_1–H_2	100	100	79	80	100	100	100		75
H_1–H_3	100	100	78	72	100	100	100		70
H_2–H_3	100	100	85	82	100	100	100		80
A–H_1	98	100	60	57	90	100	100		27
A–H_2	98	100	57	69	90	100	100		28
A–H_3	98	100	64	63	90	100	100		30

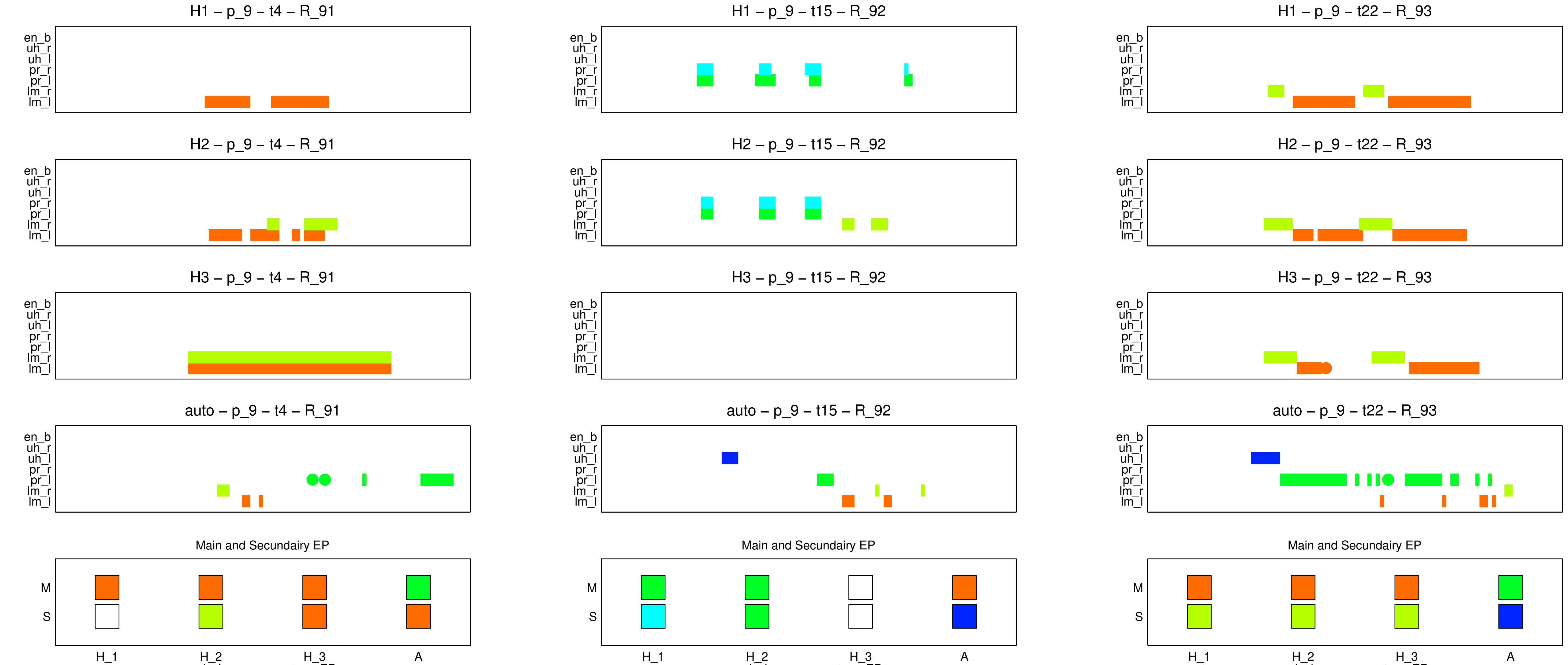

pairwise agreement per EP (t4 – R_91)

	L–l	L–r	P–l	P–r	U–l	U–r	E–b		all
H_1–H_2	91	87	100	100	100	100	100		80
H_1–H_3	77	50	100	100	100	100	100		50
H_2–H_3	76	63	100	100	100	100	100		60
A–H_1	74	96	87	100	100	100	100		63
A–H_2	77	83	87	100	100	100	100		63
A–H_3	55	54	87	100	100	100	100		41

pairwise agreement per EP (t15 – R_92)

	L–l	L–r	P–l	P–r	U–l	U–r	E–b		all
H_1–H_2	100	91	94	96	100	100	100		84
H_1–H_3	100	100	82	84	100	100	100		81
H_2–H_3	100	91	86	86	100	100	100		77
A–H_1	93	96	81	84	95	100	100		62
A–H_2	93	91	85	86	95	100	100		68
A–H_3	93	96	95	100	95	100	100		79

pairwise agreement per EP (t22 – R_93)

	L–l	L–r	P–l	P–r	U–l	U–r	E–b		all
H_1–H_2	96	94	100	100	100	100	100		92
H_1–H_3	85	89	100	100	100	100	100		80
H_2–H_3	83	93	100	100	100	100	100		81
A–H_1	58	86	59	100	92	100	100		38
A–H_2	58	80	59	100	92	100	100		39
A–H_3	71	79	59	100	92	100	100		40
